# Evaluation of Inbred Maize (*Zea mays* L.) for Tolerance to Low Phosphorus at the Seedling Stage

**DOI:** 10.3390/plants12132520

**Published:** 2023-06-30

**Authors:** Md. Shalim Uddin, Farzana Akter, Md. Golam Azam, Shamim Ara Bagum, Neelima Hossain, Masum Billah, Priya Lal Biswas, Abu Sayeed Md. Hasibuzzaman, Abul Bashar Mohammad Khaldun, Amnah Mohammed Alsuhaibani, Ahmed Gaber, Akbar Hossain

**Affiliations:** 1 Institute of Crop Sciences, Graduate School of Chinese Academy of Agricultural Sciences (GSCAAS), Haidian District, Beijing 100081, China; 2 Department of Genetics and Plant Breeding, Bangabandhu Sheikh Mujibur Rahman Agricultural University, Gazipur 1706, Bangladesh; 3 Bangladesh Agricultural Research Institute, Gazipur 1701, Bangladesh; 4 Institute of Cotton Research, Chinese Academy of Agricultural Sciences, Anyang 455000, China; 5 Bangladesh Rice Research Institute (BRRI), Gazipur 1701, Bangladesh; 6 Planning and Evaluation Division, Bangladesh Agricultural Research Council (BARC), Farmgate, Airport Road, Dhaka 1215, Bangladesh; 7 Department of Physical Sport Science, College of Education, Princess Nourah bint Abdulrahman University, P.O. Box 84428, Riyadh 11671, Saudi Arabia; 8 Department of Biology, College of Science, Taif University, P.O. Box 11099, Taif 21944, Saudi Arabia; 9 Division of Soil Science, Bangladesh Wheat and Maize Research Institute, Dinajpur 5200, Bangladesh

**Keywords:** genetic variability, seedling traits, hydroponic, multi-trait selection, maize genotypes

## Abstract

In underdeveloped nations where low-input agriculture is practiced, low phosphorus (LP) in the soil reduces the production of maize. In the present study, a total of 550 inbred maize lines were assessed for seedling traits under LP (2.5 × 10^−6^ mol L^−1^ of KH_2_PO_4_) and NP (2.5 × 10^−4^ mol L^−1^ of KH_2_PO_4_) hydroponic conditions. The purpose of this study was to quantify the amount of variation present in the measured traits, estimate the genetic involvement of these characteristics, examine the phenotypic correlation coefficients between traits, and to integrate this information to prepare a multi-trait selection index for LP tolerance in maize. A great deal of variability in the maize genotype panel was confirmed by descriptive statistics and analysis of variance (ANOVA). Estimated broad-sense heritability (h^2^) ranged from 0.7 to 0.91, indicating intermediate to high heritability values for the measured traits. A substantial connection between MSL and other root traits suggested that the direct selection of MSL (maximum shoot length) could be beneficial for the enhancement of other traits. The principal component analysis (PCA) of the first two main component axes explained approximately 81.27% of the variation between lines for the eight maize seedling variables. TDM (total dry matter), SDW (shoot dry weight), RDW (root dry weight), SFW (shoot fresh weight), RFW (root fresh weight), MRL (maximum root length), and MSL measurements accounted for the majority of the first principal component (59.35%). The multi-trait indices were calculated based on PCA using all the measured traits, and 30 genotypes were selected. These selected lines might be considered as the potential source for the improvement of LP tolerance in maize.

## 1. Introduction

Maize is an extensively grown crop for fodder, food, and fuel across humid and temperate regions of the world [1]. In low- and middle-income nations around the world, maize, wheat, and rice account for 55–70% of calories [2]. In the Americas, Africa, and Asia combined, maize and its products account for 30% of the total food source [3]. Several natural risks, including floods, water scarcity, salt, and low phosphorus levels, hinder crop yield [4]. A lack of phosphorus (P) is regarded as one of the factors limiting agricultural production [5]. Low soil P affects more than 30% of the world’s maize production [6]. P is essential for the plant’s capacity to carry out several functions, including energy production, amino acid synthesis, photosynthesis, glycolysis, respiration, membrane formation and stability, enzyme activation/inactivation, redox processes, signaling, carbohydrate metabolism, and nitrogen fixation [6]. Phosphorus is regarded as a crucial, non-renewable chemical for agricultural output. The phosphorus comes from rock phosphate, which is also a finite source [7]. Western Sahara, Morocco, and China account for more than 58% of all phosphate that has been reported on Earth [8]. Due to its role in plant development, phosphorus (P) is often an important resource that must be carefully managed. The demand for P fertilizer has been rapidly increasing because of its evident importance for crop yield [9]. Due to its role in various essential components, such as DNA and RNA, P is essential to all life. In fact, organisms require higher concentrations of P to grow rapidly. However, the production of crops requires more P than is available in the environment [10]. It has been estimated that the low availability of P affects more than two billion hectares of agricultural land worldwide and P deficiency directly leads to a significant decrease (5–15%) in crop yields [11]. Foliar redness and necrosis are two indicators of phosphorus deficiency that plants exhibit when they have low levels. For developing countries and rich countries alike, crops that flourish in low-phosphorus conditions would improve food security [12]. Food production and economic growth in low-input agriculture are hindered by a lack of P availability in poor nations [13]. Phosphorus uptake by roots is influenced by a variety of parameters, including soil colloidal chemistry, soil pH, and microbial activity, all of which are important factors for the reduced movement of P in soil. Phosphorus deficiency induces numerous morphological, physiological, and architectural changes in plant root systems. However, the genetic change traits of plants enhance phosphorus uptake under low P availability, and this change can increase the efficacy of phosphorus consumption in maize [14,15,16,17,18].

The use of best linear unbiased predictors (BLUPs) instead of arithmetic means is utilized to analyze spatial variance between environments, imbalanced data, and pedigree linkages. To breed for a complex trait like low-P tolerance or avoidance in plants, one must consider several elements, such as understanding the physiological, genetic, and molecular underpinnings of the phenotype, as well as the interactions of genotypes with their environment. Recently, researchers studied maize grown in a hydroponic culture under low-phosphorus conditions at the seedling stage [19]. The purpose of this research was to recognize the low-P-tolerant and low-P-sensitive inbred maize from the collection of 550 inbred lines (Supplementary Table S1). This will be useful for further genetic studies and to include P tolerance as a universally significant trait into maize improvement through the use of a multi-trait selection index for LP tolerance.

## 2. Results

### 2.1. Analysis of Variance (ANOVA) and Frequency Distribution of Traits

The genotype pool comprised 550 genotypes (Supplementary Table S1) and was tested for tolerance to LP. The ANOVA revealed a significant effect of genotype and treatment as well as a significant genotype by treatment interaction (Table 1).

**Figure 1:**
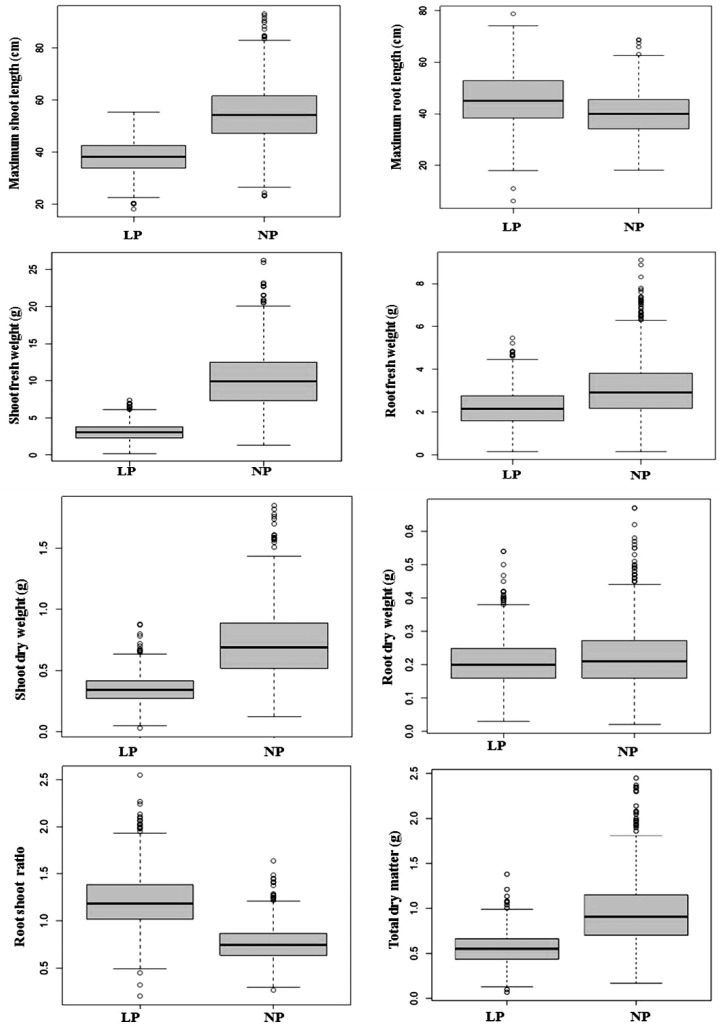
Effect of LP and NP treatments on maize genotypes for measured traits. The box’s upper and lower quantiles are represented by the box’s edges, while the median is shown in the box’s center. MSL = maximum shoot length, MRL = maximum root length, SFW = shoot fresh weight, RFW = root fresh weight, SDW = shoot dry weight, RDW = root dry weight, RSR = root shoot ratio, TDM = total dry matter, NP = normal phosphorus, LP = low phosphorus.

**Figure 2:**
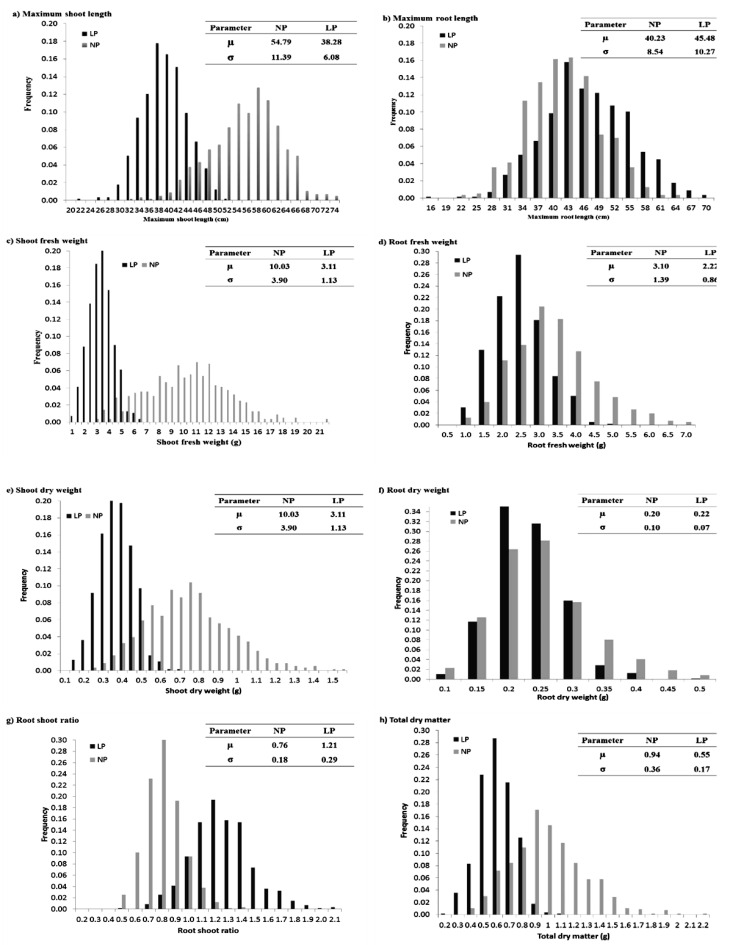
Frequency distribution for traits measured under LP and NP conditions. MSL = maximum shoot length, MRL= maximum root length, SFW = shoot fresh weight, RFW = root fresh weight, SDW = shoot dry weight, RDW = root dry weight, RSR = root shoot ratio, TDM = total dry matter, NP = normal phosphorus, LP = low phosphorus.

In the box plot, the upper and lower quantile is indicated by the box edges, and the median is displayed in the middle of the box. The individuals that fall outside of the rank of whisker are shown as circles (Figure 1). Normal distribution was observed for all the traits for both LP and NP conditions, except a few traits skewed left and some skewed right (Figure 2).

### 2.2. Estimated Heritabilities 

**Figure 3:**
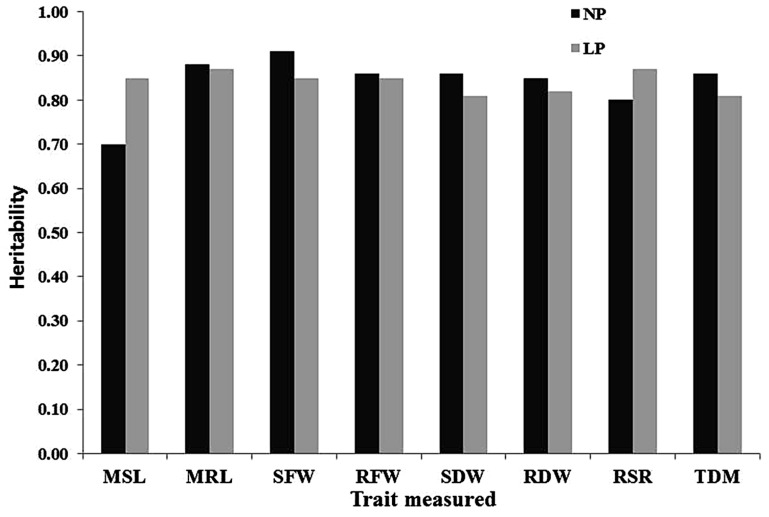
Estimated heritabilities for eight traits. MSL = maximum shoot length, MRL= maximum root length, SFW = shoot fresh weight, RFW = root fresh weight, SDW = shoot dry weight, RDW = root dry weight, RSR = root shoot ratio, TDM = total dry matter, NP = normal phosphorus, LP = low phosphorus.

Under NP conditions, the estimated broad-sense heritabilities (h^2^) ranged from 0.7 to 0.9, while under LP, they ranged from 0.81 to 0.87. SFW, MRL, and TDM had consistently higher h^2^ estimates than other traits under NP conditions. On the other hand, RSR and MRL had consistently higher h^2^ estimates than other traits under LP conditions (Figure 3).

### 2.3. Phenotypic Correlation Coefficients under Low-Phosphorus (LP) Conditions

Under LP conditions, the pairwise phenotypic (lower diagonal) correlation coefficients for all traits are presented in Table 2.

In terms of phenotypic levels, TDM possessed the maximum positive phenotypic (r_p_) correlation coefficients with SDW (r_p_ = 0.96 **), RDW r_p_ = 0.88 **), SFW (r_p_ = 0.85 **), and RFW (r_p_ = 0.81 **). MSL had a moderately significant and positive correlation with SFW (r_p_ = 0.71 **), SDW (r_p_ = 0.76 **), RDW (r_p_ = 0.73 **), and TDM (r_p_ = 0.64 **). MRL had a moderately positive significant correlation coefficient with RSR (r_p_ = 0.75 **). A significant negative correlation was found for RSR with MSL (r_p_ = −0.42 **). Other correlations were neither strong nor significant.

### 2.4. Phenotypic Correlation Coefficients under NP Conditions

Under NP conditions, the pairwise phenotypic (lower diagonal) correlation coefficients for all traits are presented in Table 3. Regarding phenotypic levels, TDM resulted in the maximum positive r_p_ with SDW (r_p_ = 0.99 **), RDW (r_p_ = 0.88 **), SFW (r_p_ = 0.88 **), and RFW (r_p_ = 0.78 **). MSL had a moderately positive significant correlation with SFW (r_p_ = 0.70 **), SDW (r_p_ = 0.67 **), RDW (r_p_ = 0.49 **), and TDM (r_p_ = 0.65 **) MRL also had a moderately significant and positive correlation with RSR (r_p_ = 0.75 **). A significant negative correlation was found for RSR with MSL (r_p_= −0.46 **). Other correlations were neither strong nor significant. For improvement of P use efficiency in maize, selection can be made mainly based on shoot length and shoot dry weight.

### 2.5. Multivariate Analysis

**Figure 4:**
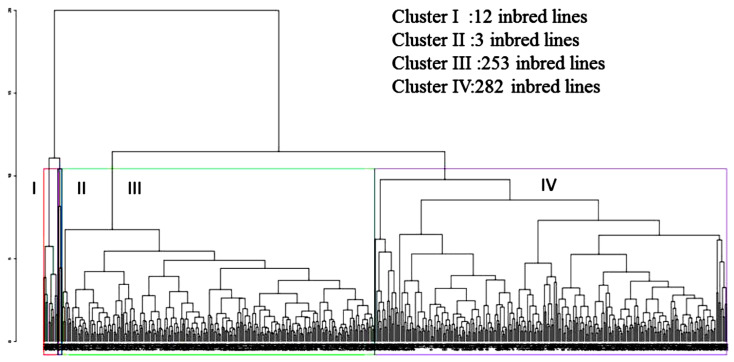
Dendrogram from UPGMA clustering for 550 inbred maize lines using Euclidean genetic distances based on all seedling traits measured.

Multivariate analysis was performed with relative trait values. The first three PCs described 89.78% of the entire variability among the lines for the studied traits (Table 4). The first PC accounted for 59.35% of the entire variation, which was mostly explained by TDM, SDW, RDW, SFW, RFW, MRL, and MSL measurements. RSR, MRL, and MSL were the most predominant characters for the second and third PC, which contributed 21.92% and 8.51% of the total variation, respectively. Seedling trait means were used to enumerate the Euclidian distance coefficients for all inbred maize lines (Figure 4).

Two main groups for the 550 inbred lines (Supplementary Table S1) were obtained through cluster analysis. The first cluster accommodated 535 lines with the lowest MSL, and an arrested root system considering MRL, SDW, and RDW, while the second cluster comprised all of the other lines with large-sized roots with SDW, RDW, and RSR (Figure 4).

The first cluster had 12 inbred lines (i.e., C5 RIL 139, C5 RIL 207, 218 [CML395/CML440//[LPSC3H144-1-2-2-2-4-#-BB/SC/ZM605#b-19-2-X]-1-2-X-1-1-BB]-1-2- 219 1-1-B]-3-2-1-1-BBB-B-B-B, CML114, CML115, CML118, CML380xMBR/MDR C3 BC F21-1- 220 1-2-B-B-B-B-3-1-B-B-B, CML402, CML430, CML433, CML99, and CML412, of which, dis- 221 had the highest MSL, MRL, SDW, and RDW). In contrast, the second cluster group contained only three lines (i.e., C5 RIL 217, [CML444/ZSR923S4BULK-2-2-X-X-X-X-1-BB]-1-1-1-2/CML441]-1-1-1- 2232-BBB-B-B-B, and CML96), with extreme values of MSL, MRL, SDW, and RDW. The third cluster was split into two subgroups, in which the first consisted of 253 lines that displayed the lowest MSL, MRL, SDW, and RDW. In contrast, the fourth cluster group contained 282 lines with moderate values of MSL, MRL, SDW, and RDW. The average genetic distance was 3.53, varying from 0.25 to 20.01. The maximum genetic distance (20.01) was obtained between inbred lines 184 (C6 RIL 79) and 347 ([CML444/ZSR923S4BULK-2-2-X-X-X-X-1-BB]-1-1-1-2/CML441]-1-1-1-2-BBB-B-B-B).

### 2.6. Selection of Genotypes with a Multi-Trait Index

**Figure 5:**
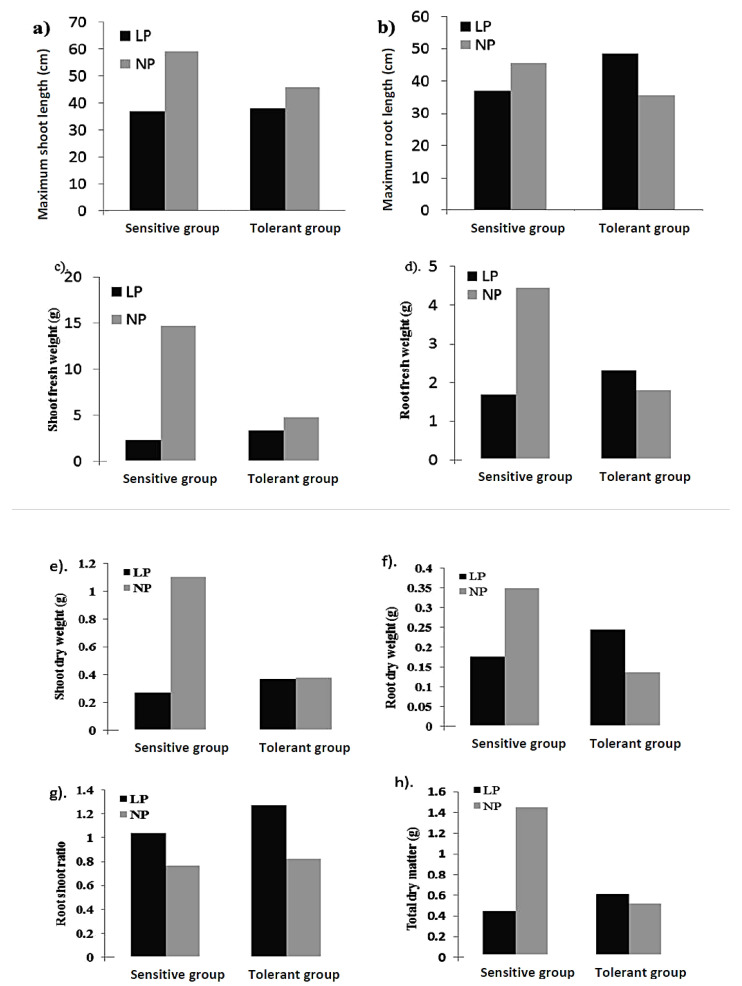
Seedlings traits of sensitive and tolerant group of inbred lines under different phosphorus regimes. Note: (**a**) Shoot and (**b**) Root length; (**c**) Shoot and (**d**) Root fresh weight; (**e**) Shoot and (**f**) Root dry weight; (**g**) Root shoot ratio and (**h**) Total dry weight. NP = normal phosphorus, LP = low phosphorus.

A total of 30 LP-tolerant lines were selected based on LPTI_all, which comprised 9 from the C5 population, 8 from the C6 population, 12 from the tropical population, and 1 from the temperate population. On the other hand, there were 30 selected sensitive lines; 29 from the C6 population and 1 from the tropical population. To confirm the selection based on LPTI_all, selected sensitive and tolerant groups were compared for all of the traits analyzed (Figure 5).

In the sensitive group, MSL was reduced by 37.31% under LP compared with NP, while it was reduced by 17.03% in the tolerant group. MRL increased by 26.55% under LP conditions in the tolerant group, while in the sensitive group, it was reduced by 18.89%. This means that our selection procedure was on the right track. Similarly, other traits like SFW, SDW, and TDM reduced by 84.61, 75.06, and 69.17%, respectively, in the sensitive group under LP, while other related traits, namely RFW, RDW, and RSR, increased by 21.34, 44.16, and 38.85%, respectively, in the tolerant group.

### 2.7. LP Tolerance and P Responsiveness

**Figure 6:**
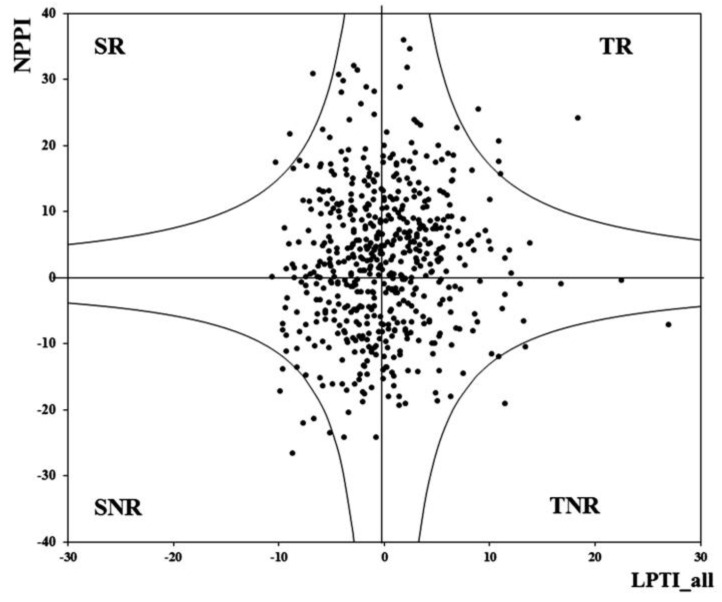
Classification of the genotypes based on LP tolerance and P responsiveness. TR, tolerant and responsive; SR, sensitive and responsive; TNR, tolerant and non-responsive; and SNR, sensitive and non-responsive.

Based on both selection traits, LPTI_all (LP tolerance indicator) and NPPI (NP performance indicator), at 5% of the threshold level, we selected five, four, three, and six inbred lines from the group’s TR (tolerant and responsive), TNR (tolerant and non-responsive, SR (sensitive and responsive), and SNR (sensitive and non-responsive) groups, respectively (Figure 6).

**Figure 7:**
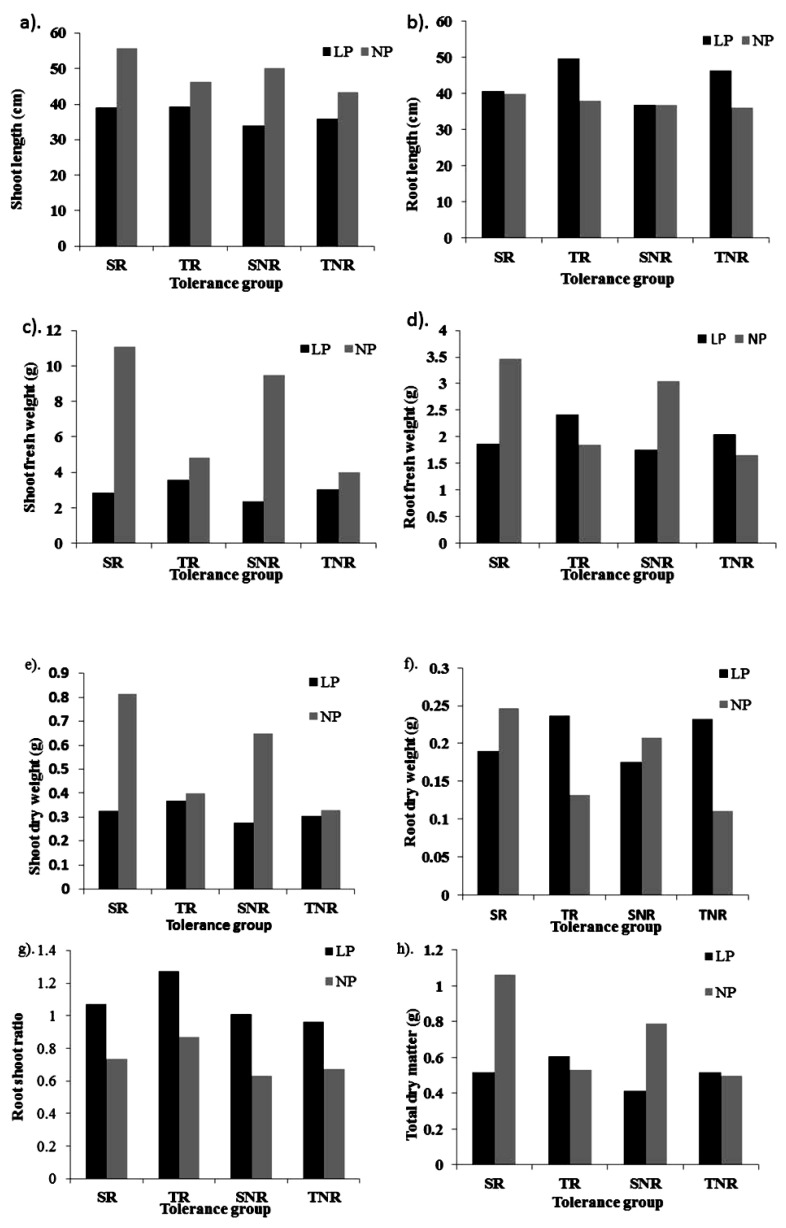
Classification of inbred lines based on both selection traits of LPTI_all (low-phosphorus tolerance indicator) and NPPI (normal phosphorus performance indicator). Note: (**a**) Shoot and (**b**) Root length; (**c**) Shoot and (**d**) Root fresh weight; (**e**) Shoot and (**f**) Root dry weight; (**g**) Root shoot ratio and (**h**) Total dry weight. TR, tolerant and responsive; SR, sensitive and responsive; TNR, tolerant and non-responsive; and SNR, sensitive and non-responsive.

The genotypes in the TR group not only performed well under LP conditions but also responded to P fertilizer application (Figure 7). We selected five lines comprising one from tropical, two from C6, one from C5, and one from temperate populations (Table 5).

The contrasting group, SNR, performed worse under LP conditions and did not respond to NP treatment. The selected SNR group included six lines from the C6 population. From the TNR group, four lines were selected with one from C5, one from C6, and two from tropical populations. In the SR group, three lines were selected from the C6 population. From this result, it is clear that the C6 population is more diverse than other populations. MSL was reduced by 29.7, 15.4, 32.2, and 17.2% for SR, TR, SNR, and TNR groups, respectively, under LP conditions (Figure 7). A similar trend was also observed for shoot-related traits. For root-related traits, an increase in phenotypic values was observed under LP conditions compared with NP conditions for the tolerance groups. Maximum MRL, RDW, and RSR were found to be increased in the TR group compared with the other groups.

## 3. Discussion

In a high-light plant development room, morphological variation in the maize genotype panel investigated was shown to be substantial in terms of seedling traits evaluated under LP and NP conditions. The lengthy and wide MSL, MRL, and RSR genotypes found in this study are ideal for fostering LP tolerance. Maize inbred lines have also been found to have a wide range of adaptability based on low shoot dry weight, a strong root system, and the prominent root-to-shoot ratio at the seedling stage, leading to an indication of LP-tolerant genotypes [12,20,21,22,23,24]. For the uptake of both mobile nutrients such as NO_3_ and immobile nutrients, a robust root and shoot with a long and well-distributed root system is necessary, particularly under deficient nutrient conditions [25]. While our work has found maize inbred lines with a long and enlarged root system under LP conditions, it has also identified genetic areas that influence these traits.

The hydroponic evaluation of maize genotypes provides a satisfactory method for identifying maize lines with superior morphological traits [26]. Genetic analysis of LP tolerance has been conducted using bi-parental populations in many plants [27,28,29,30]. The estimated genetic components of variance for the majority of traits were modest to moderate. Due to extensive selection, the genotype panel includes cultivars and breeding lines with low levels of genetic diversity. Even more so, a lack of variation in trait expression could indicate a complex genetic inheritance and/or a high environmental influence [31]. Generally, advanced quantitative trait selection methods lead to helping the estimation of heredity and variance components more precisely to determine the selection of genetic gain. Potentially, phenotypic selection of seedling traits such as increasing the plant height and root length of mature plants could lead to assistance for the selection of greater LP-tolerant genotypes [32,33].

Principal component analysis (PCA) showed that seedling traits such as MSL, RDW, and RSR are responsible for the majority of the phenotypic variance seen across the investigated maize lines. Focusing on MSL, RLS, and RFW appeared to be sufficient for future studies on the divergence of maize seedlings and the evaluation of LP tolerance. Due to its ease of measurement, MSL can be used as an indirect trait for depicting TDM. Classifying LP tolerance based on the relative feature is the most consistent in screening experiments [22]. The results revealed that SFW, MRL, and TDM under NP conditions, and RSR and MRL under LP conditions had higher heritability compared to others. Some researchers observed that the root dry weight, shoot dry weight, maximum shoot length, and total dry weight of maize were highly heritable in low-phosphorus conditions [19].

Cluster analysis can be used to classify genotypes and determine which cluster is best. Cluster analysis is a multivariate analysis that minimizes differences within clusters while maximizing differences between clusters [34]. The inbred lines utilized in this study did not form clusters according to their genetic background and origin, which means a high level of variation is present among and within different backgrounds. Cluster analysis had been carried out in various studies based on quantitative traits for the selection of LP-tolerant maize [35,36,37]. In this experiment, it was found that the data were normally distributed through boxplot analysis. Azam al. [19] also observed the normality of data by boxplot analysis while screening maize genotypes under low-phosphorus conditions.

Based on LPTI, 30 LP-tolerant lines were chosen, with 9 from the C5 population, 8 from the C6 population, 12 from the tropical population, and 1 from the temperate population.

## 4. Materials and Methods

### 4.1. Experimental Site

The experiment was carried out in a high-lux plant growth room at the Institute of Crop Sciences, Graduate School of Chinese Academy of Agricultural Sciences (GSCAAS), Haidian District, Beijing 100081, China.

### 4.2. Plant Materials

The genotype panel included 550 maize genotypes (Supplementary Table S1) chosen to symbolize a variety of variability, including inbred lines and two recombinant inbred lines (RILs). C5 (Ac7643Ac7729/TZSRW, accommodating 123 lines) and C6 (CML444Malawi, accommodating 215 lines) RIL populations were used. The RIL populations were produced at the International Maize and Wheat Improvement Center from tropical inbred lines (CIMMYT). Among the inbred lines, 143 tropical/subtropical lines were collected from CIMMYT and 69 temperate inbred lines were collected from the Chinese Academy of Agricultural Sciences (CAAS).

### 4.3. Plant Growth Conditions

For phenotyping, a high-lux hydroponic plant developing compartment was employed, with day-to-day temperatures maintained at 28 °C in the light for 14 h and 22 °C in the dark for 10 h. The growth room’s light intensity was 657 μmol m^−2^ s^−1^, and the relative humidity was 50%.

The experiment was accompanied by randomized completely block design (RCBD). Two P treatments were used: LP (2.5 × 10^−6^ mol L^−1^ KH_2_PO_4_) and NP (2.5 × 10^−4^ mol L^−1^ KH_2_PO_4_), both replicated twice with several blocks (2522). Uddin et al. [38] determined the chemical composition of nutrients for both the LP and NP groups, which were used in this experiment.

Twenty-five (25) seedlings were surface sterilized for ten minutes with 3% NaOCl, followed by three rounds of washing with deionized water. After surface disinfection, kernels were positioned underneath a “water-saturated” quartz particle and enclosed with a black plastic sheet for four days in the development hollow to stimulate sprouting, followed by an additional four days beneath a light. The endosperm was removed from the sprouts before planting. Then, they were concealed in a sponge before being placed in a hole in the covering. In each of the six openings in the covering, six plants were planted. Following transplantation, a nutritious solution was refilled every two days with continuous air. The pot was then brushed clean to prevent fungal growth. The cultures grew in the nutrient solution for a total of 15 days [38].

After culturing on the 15th day in the nutrient solution, the seedlings were removed from the culture for the measurement of the traits listed below.

Recorded data included:

Maximum root length (cm) (MRL): from the coleoptile node to the last tip of the primary root.

Maximum shoot length (cm) (MSL): from coleoptile node to highest tip of the leaf.

Root fresh weight (g) (RFW): root weight after removing the water from roots.

Shoot fresh weight (g) (SFW): shoot weight after removing the water from the shoot.

Root dry weight (g) (RDW): after oven drying at 65 °C for 7 day.

Shoot dry weight (g) (SDW): after oven drying at 65 °C for 7 days.

Root length to shoot length ratio (RSR): root length divided by shoot length.

Total dry matter (g) (TDW): root dry weight + shoot dry weight.

### 4.4. Statistical Analysis

For both the individual and combined analyses, a linear model with RCBD was used with two replications. The statistical analyses were conducted using the R-statistics software version 3.0.2 for Windows [39]. For each trait, components of phenotypic variation were estimated with constrained maximum likelihood approaches using analysis of variance. The variance components were estimated using the lme4 package’s linear mixed effect “lmer” command [40].

#### 4.4.1. Multivariate Analysis

The Euclidean distance coefficients were calculated for all pairs of genotypes using the Statistical Tool for Agricultural Research (STAR) version 2.0.1 software [41]. The seedling mean data were used to generate a Euclidean distance matrix, which was then used as the input for cluster analysis using the unweighted pair-group method of arithmetic average (UPGMA) and principal component analysis (PCA). This identified the major traits causing variation among the tested inbred lines. A UPGMA dendrogram was created by calculating the range of similarity between the lines using Euclidean genetic distances.

#### 4.4.2. Calculation of the Multi-Trait Selection Index

The relative values of the trait were standardized and subjected to PCA using SAS [42]. The PCs whose eigenvalues were larger than 1 were retained [42] and exploited to estimate the LP tolerance indicator (LPTI_all) using the following formula:

$$\mathrm{LPTI}=\overset{n}{\underset{i=1}{\sum }}\left(\mathrm{PCi}\;\times \mathrm{CRi}\right)$$

Here, n indicates the number of PCs whose eigenvalues are larger than 1 [43], and CR (contribution rate) highlights the rate for trait variation. Based on LPTI_all at a 5% selection intensity, we selected the 30 most tolerant and 30 most susceptible lines.

Inbred lines with improved phenotypic performance under increased P-level conditions were referred to as P responders, whereas those with the opposite performance were referred to as non-responders [43]. The NPPI (NP performance indicator) was defined in this study as the trait performance under NP conditions. It was calculated as follows: the standardized values of each trait were subjected to PC analysis, and the PCs with eigenvalues greater than 1 were utilized to calculate the NPPI as follows:

$$\mathrm{NPPI}=\overset{n}{\underset{i=1}{\sum }}\left(\mathrm{PCi}\;\times \mathrm{CRi}\right)$$

where n denotes the number of PCs with an eigenvalue greater than 1 and CR denotes the rate of trait variation. Based on the two criteria for P responsiveness and LP tolerance, the examined genotypes were categorized into four groups: tolerant and responsive (TR), sensitive and responsive (SR), tolerant and non-responsive (TNR), and sensitive and non-responsive (SNR). To choose the extreme lines from each of the four groups, the top 5% of NPPI and LPTI_all values were used.

## 5. Conclusions

To determine the LP tolerance of maize genotypes, a total of 550 maize genotypes were assessed for the seedling traits under both LP and NP hydroponic conditions. Based on the descriptive statistics, analysis of variance, estimation of broad-sense heritability, genotype × traits biplot analysis, average genetic distance, cluster analysis, and PCA using all of the measured traits, 30 genotypes were selected as the potential source for the improvement of LP tolerance in maize. Higher heritability was found for SFW, MRL, and TDM under NP conditions, and for RSR and MRL under LP conditions. The present study offers guidelines for plant breeders to develop low-phosphorus-tolerant maize cultivars for the sustainability of maize production.

## Figures and Tables

**Table 1:** Combined analysis of variance of the tested traits.

Source of Variation	Mean Square								
	Df	MSL	MRL	SFWP	RFW	SDW	RDW	RSR	TDM
Replication	1	3185.98	18.17	4.57	0.79	0.002	0.001	0.52	0.002
Treatment	1	151,909.92	15,335.49	26,685.54	430.54	78.381	0.148	113.40	85.344
Genotype	549	176.87	222.85	18.14	3.13	0.094	0.015	0.13	0.168
Genotype × Treatment	549	77.25	94.15	11.87	1.52	0.064	0.009	0.07	0.107
Error	1098	33.67	20.14	1.46	0.34	0.011	0.002	0.01	0.020
Significance level		***	***	***	***	***	***	***	***

MSL = maximum shoot length, MRL = maximum root length, SFW = shoot fresh weight, RFW = root fresh weight, SDW = shoot dry weight, RDW = root dry weight, RSR = root shoot ratio, TDM = total dry matter, NP = normal phosphorus, LP= low phosphorus, *** F test for genotype, treatment, and genotype × treatment interaction significant at the 0.001 level of probability.

**Table 2:** Phenotypic (lower diagonal) correlations under LP conditions.

	MSL	MRL	SFW	RFW	SDW	RDW	RSR	TDM
MSL		0.26 *	0.74 **	0.49 **	0.65 **	0.51 **	−0.42 **	0.64 **
MRL	0.27 *		0.41 **	0.59 **	0.43 **	0.44 **	0.76 **	0.47 **
SFW	0.71 **	0.40 **		0.73 **	0.87 **	0.66 **	−0.11	0.85 **
RFW	0.71 **	0.57 **	0.74 **		0.76 **	0.73 **	0.23	0.81 **
SDW	0.76 **	0.41 **	0.85 **	0.74 **		0.71 **	−0.02	0.96 **
RDW	0.73 **	0.43 **	0.66 **	0.73 **	0.71 **		0.07	0.88 **
RSR	−0.41 **	0.75 **	−0.11	0.20	−0.04	0.06		0.01
TDM	0.64 **	0.45 **	0.84 **	0.79 **	0.96 **	0.89 **	0.01	

MSL = maximum shoot length, MRL = maximum root length, SFW = shoot fresh weight, RFW = root fresh weight, SDW = shoot dry weight, RDW = root dry weight, RSR = root shoot ratio, TDM = total dry matter, NP = normal phosphorus, LP = low phosphorus, *, ** indicate significance at 5% and 1% levels of probability, respectively.

**Table 3:** Phenotypic (lower diagonal) correlation under NP conditions.

	MSL	MRL	SFW	RFW	SDW	RDW	RSR	TDM
MSL		0.41 **	0.81 **	0.63 **	0.78 **	0.57 **	−0.46 **	0.76 **
MRL	0.36 *		0.47 **	0.41 **	0.39 *	0.33 *	0.60 **	0.39 *
SFW	0.70 **	0.45 **		0.76 **	0.87 **	0.74 **	−0.23	0.88 **
RFW	0.55 **	0.41 **	0.75 **		0.73 **	0.81 **	−0.13	0.78 **
SDW	0.67 **	0.38 *	0.87 **	0.72 **		0.79 **	−0.28 *	0.99 **
RDW	0.49 **	0.32 *	0.74 **	0.80 **	0.79 **		−0.17	0.88 **
RSR	−0.51 **	0.58 **	−0.21	−0.11	−0.25	−0.15		−0.27 *
TDM	0.65 **	0.38 *	0.87 **	0.77 **	0.99 **	0.88 **	−0.24 *	

MSL = maximum shoot length, MRL= maximum root length, SFW = shoot fresh weight, RFW = root fresh weight, SDW = shoot dry weight, RDW = root dry weight, RSR = root shoot ratio, TDM = total dry matter, NP = normal phosphorus, LP = low phosphorus, *, ** indicate significance at 5% and 1% levels of probability, respectively.

**Table 4:** The first four PCs of trait eigenvectors in maize genotypes.

Parameter	PC1	PC2	PC3	PC4
Maximum shoot length (cm)	0.27	−0.40	−0.72	0.12
Maximum root length (cm)	0.26	0.51	−0.53	0.15
Shoot fresh weight (g)	0.42	−0.09	0.00	−0.25
Root fresh weight (g)	0.39	0.06	0.05	−0.82
Shoot dry weight (g)	0.42	−0.04	0.20	0.26
Root dry weight (g)	0.39	−0.02	0.33	0.28
Root shoot ratio	0.04	0.75	0.01	0.02
Total dry matter (g)	0.44	−0.03	0.24	0.29
Cumulative% of the total variance	59.35	81.27	89.78	94.23

**Table 5:** Selection of two types of extreme lines based on multi-trait criteria.

Tolerance Group		C5	C6	Tropical	Temperate	Total
LPTI all	Sensitive	0	29	1	0	30
	Tolerant	9	8	12	1	30
TR		1	2	1	1	5
TNR		1	1	2	0	4
SNR		0	6	0	0	6
SR		0	3	0	0	3

TR, tolerant and responsive; SR, sensitive and responsive; TNR, tolerant and non-responsive; and SNR, sensitive and non-responsive.

## Data Availability

Data recorded in the current study are available in all Tables and Figures of the manuscript and also in the Supplementary data file.

## Supplementary Materials

The following supporting information can be downloaded at: https://www.mdpi.com/article/10.3390/plants12132520/s1, Table S1: 550 maize inbred lines used in the current study.
