# Supplementary material for: Evaluation of Inbred Maize (Zea mays L.) for Tolerance to Low Phosphorus at the Seedling Stage
Source: Plants (Basel). 2023 Jun 30;12(13):2520. doi: 10.3390/plants12132520 (PMC10346422; doi:10.3390/plants12132520)
Supplement: Supplementary file 1 [file plants-12-02520-s001.zip › plants-2366351-supplementary.pdf]

Table S1. 550 maize inbreds used in the current study

Seed ID	Acc	Gen	trt.	rep	SL	RL	SFW	RFW	SDW	RDW	RSR	TDM
11JY1255	C5 RIL 2	1	LP	1	35.52	49.31	3.27	3.53	0.43	0.26	1.39	0.69
11JY1256	C5 RIL 3	2	LP	1	38.19	60.98	2.78	2.43	0.45	0.20	1.60	0.64
11JY1258	C5 RIL 5	3	LP	1	29.19	41.31	2.33	1.76	0.33	0.14	1.42	0.47
11JY1259	C5 RIL 6	4	LP	1	40.19	46.31	3.12	2.15	0.47	0.19	1.15	0.66
11JY1262	C5 RIL 10	5	LP	1	29.52	44.31	1.71	1.81	0.26	0.14	1.50	0.39
11JY1263	C5 RIL 11	6	LP	1	40.19	48.64	3.24	2.97	0.43	0.22	1.21	0.65
11JY1264	C5 RIL 12	7	LP	1	35.86	62.98	2.84	2.91	0.36	0.20	1.76	0.56
11JY1267	C5 RIL 17	8	LP	1	33.86	45.98	3.56	2.74	0.47	0.20	1.36	0.66
11JY1268	C5 RIL 18	9	LP	1	34.52	47.31	3.60	3.15	0.46	0.21	1.37	0.67
11JY1269	C5 RIL 19	10	LP	1	39.86	46.31	4.39	3.76	0.53	0.28	1.16	0.81
11JY1271	C5 RIL 21	11	LP	1	35.86	51.31	3.34	3.13	0.48	0.26	1.43	0.73
11JY1272	C5 RIL 24	12	LP	1	29.19	37.64	2.37	2.26	0.34	0.18	1.29	0.51
11JY1274	C5 RIL 26	13	LP	1	36.86	52.98	3.45	2.70	0.46	0.20	1.44	0.66
11JY1275	C5 RIL 27	14	LP	1	32.19	40.64	2.15	2.28	0.36	0.16	1.26	0.52
11JY1276	C5 RIL 28	15	LP	1	32.86	35.98	2.20	1.27	0.32	0.11	1.09	0.44
11JY1278	C5 RIL 30	16	LP	1	30.19	51.64	2.65	2.83	0.41	0.25	1.71	0.66
11JY1279	C5 RIL 31	17	LP	1	31.86	55.31	3.71	3.23	0.50	0.24	1.74	0.74
11JY1280	C5 RIL 32	18	LP	1	36.19	43.31	3.29	3.04	0.50	0.24	1.20	0.74
11JY1281	C5 RIL 34	19	LP	1	28.19	38.98	2.82	2.65	0.38	0.19	1.38	0.57
11JY1282	C5 RIL 36	20	LP	1	34.58	36.81	2.83	2.13	0.30	0.14	1.06	0.44
11JY1283	C5 RIL 38	21	LP	1	34.91	38.81	3.73	3.72	0.42	0.23	1.11	0.65
11JY1284	C5 RIL 39	22	LP	1	35.91	40.14	3.06	2.13	0.44	0.16	1.12	0.60
11JY1285	C5 RIL 40	23	LP	1	38.91	44.81	4.25	3.50	0.54	0.25	1.15	0.79
11JY1286	C5 RIL 41	24	LP	1	36.24	28.14	3.30	2.16	0.41	0.19	0.78	0.60
11JY1287	C5 RIL 42	25	LP	1	31.58	48.81	2.26	2.21	0.30	0.16	1.55	0.46
11JY1288	C5 RIL 44	26	LP	1	33.24	40.81	1.31	1.82	0.39	0.10	1.23	0.48
11JY1289	C5 RIL 45	27	LP	1	32.58	35.48	2.20	1.85	0.33	0.14	1.09	0.47
11JY1290	C5 RIL 46	28	LP	1	34.58	49.14	2.43	2.28	0.33	0.17	1.42	0.50
11JY1292	C5 RIL 48	29	LP	1	43.58	53.48	5.53	4.86	0.59	0.33	1.23	0.92
11JY1293	C5 RIL 49	30	LP	1	44.91	54.48	4.14	3.61	0.54	0.26	1.21	0.80
11JY1299	C5 RIL 57	31	LP	1	34.91	40.81	2.93	3.27	0.44	0.22	1.17	0.66
11JY1301	C5 RIL 59	32	LP	1	28.91	40.48	1.55	1.83	0.25	0.12	1.40	0.37
11JY1305	C5 RIL 64	33	LP	1	34.91	61.14	2.94	3.30	0.43	0.24	1.75	0.67
11JY1310	C5 RIL 68	34	LP	1	36.24	55.81	3.35	3.28	0.42	0.24	1.54	0.66
11JY1312	C5 RIL 70	35	LP	1	34.10	46.11	2.33	1.62	0.28	0.16	1.35	0.43

Seed ID	Acc	Gen	trt.	rep	SL	RL	SFW	RFW	SDW	RDW	RSR	TDM
11JY1315	C5 RIL 82	36	LP	1	34.10	36.44	3.16	1.54	0.35	0.16	1.07	0.52
11JY1316	C5 RIL 74	37	LP	1	26.44	44.11	1.87	2.02	0.28	0.20	1.67	0.48
11JY1318	C5 RIL 78	38	LP	1	37.44	51.44	3.66	2.99	0.40	0.22	1.37	0.62
11JY1319	C5 RIL 79	39	LP	1	32.10	50.78	1.51	1.46	0.21	0.13	1.58	0.34
11JY1320	C5 RIL 80	40	LP	1	32.77	50.11	1.71	2.38	0.25	0.19	1.53	0.43
11JY1323	C5 RIL 84	41	LP	1	36.10	46.78	2.89	2.13	0.37	0.21	1.30	0.58
11JY1324	C5 RIL 86	42	LP	1	37.44	52.11	4.22	3.01	0.41	0.25	1.39	0.66
11JY1326	C5 RIL 88	43	LP	1	31.44	50.78	1.90	2.29	0.29	0.20	1.62	0.48
11JY1327	C5 RIL 89	44	LP	1	33.44	42.11	1.58	1.12	0.31	0.17	1.26	0.48
11JY1328	C5 RIL 90	45	LP	1	30.10	37.11	1.77	1.29	0.28	0.16	1.23	0.44
11JY1329	C5 RIL 92	46	LP	1	37.77	48.28	3.88	2.17	0.43	0.21	1.28	0.64
11JY1333	C5 RIL 96	47	LP	1	43.10	53.44	4.45	3.64	0.54	0.31	1.24	0.85
11JY1334	C5 RIL 99	48	LP	1	39.10	60.78	5.40	4.01	0.61	0.32	1.55	0.93
11JY1336	C5 RIL 102	49	LP	1	31.44	40.44	1.85	1.77	0.30	0.18	1.29	0.48
11JY1337	C5 RIL 103	50	LP	1	33.77	46.78	3.89	2.35	0.46	0.21	1.39	0.67
11JY1339	C5 RIL 106	51	LP	1	37.10	44.78	4.28	3.32	0.49	0.29	1.21	0.78
11JY1341	C5 RIL 108	52	LP	1	30.24	52.23	1.93	2.88	0.27	0.18	1.73	0.45
11JY1349	C5 RIL 119	53	LP	1	33.74	41.73	1.91	1.88	0.23	0.11	1.24	0.35
11JY1350	C5 RIL 120	54	LP	1	30.74	45.23	2.43	2.05	0.27	0.14	1.47	0.41
11JY1351	C5 RIL 121	55	LP	1	28.24	42.23	2.61	2.50	0.26	0.14	1.50	0.40
11JY1352	C5 RIL 122	56	LP	1	37.24	47.39	3.35	1.99	0.59	0.37	1.27	0.96
11JY1354	C5 RIL 124	57	LP	1	42.58	45.06	3.41	1.91	0.40	0.18	1.06	0.58
11JY1356	C5 RIL 126	58	LP	1	34.74	48.23	3.18	2.09	0.31	0.17	1.39	0.48
11JY1357	C5 RIL 128	59	LP	1	35.58	51.73	3.37	1.77	0.41	0.20	1.45	0.61
11JY1360	C5 RIL 131	60	LP	1	31.24	55.73	2.63	2.06	0.33	0.18	1.78	0.52
11JY1362	C5 RIL 133	61	LP	1	35.91	47.39	3.83	2.38	0.44	0.20	1.32	0.64
11JY1363	C5 RIL 134	62	LP	1	37.24	61.06	3.40	2.07	0.38	0.19	1.64	0.57
11JY1364	C5 RIL 135	63	LP	1	35.58	44.39	3.93	2.60	0.43	0.18	1.25	0.61
11JY1365	C5 RIL 137	64	LP	1	32.24	50.23	2.72	2.19	0.31	0.14	1.56	0.45
11JY1366	C5 RIL 138	65	LP	1	32.24	61.06	2.89	2.13	0.36	0.19	1.89	0.54
11JY1367	C5 RIL 139	66	LP	1	37.74	52.73	2.86	2.12	0.32	0.19	1.40	0.51
11JY1368	C5 RIL 140	67	LP	1	31.91	39.39	2.46	1.56	0.25	0.11	1.23	0.36
11JY1369	C5 RIL 141	68	LP	1	35.91	51.06	3.74	2.54	0.41	0.17	1.42	0.57
11JY1370	C5 RIL 142	69	LP	1	31.24	32.73	2.42	1.19	0.24	0.11	1.05	0.35
11JY1372	C5 RIL 144	70	LP	1	37.58	65.06	4.66	3.99	0.54	0.28	1.73	0.82
11JY1374	C5 RIL 146	71	LP	1	33.41	61.81	2.99	3.53	0.30	0.24	1.85	0.54

Seed ID	Acc	Gen	trt.	rep	SL	RL	SFW	RFW	SDW	RDW	RSR	TDM
11JY1376	C5 RIL 150	72	LP	1	34.74	65.81	3.11	3.37	0.28	0.22	1.89	0.51
11JY1377	C5 RIL 151	73	LP	1	35.80	54.95	1.70	1.32	0.18	0.15	1.53	0.33
11JY1378	C5 RIL 152	74	LP	1	33.55	49.20	1.85	1.63	0.20	0.18	1.47	0.38
11JY1379	C5 RIL 153	75	LP	1	35.80	45.60	2.09	1.86	0.25	0.17	1.27	0.42
11JY1382	C5 RIL 156	76	LP	1	35.30	41.65	1.92	0.98	0.23	0.14	1.18	0.37
11JY1383	C5 RIL 157	77	LP	1	36.53	43.13	2.31	1.15	0.23	0.13	1.18	0.36
11JY1384	C5 RIL 158	78	LP	1	35.33	60.33	2.64	2.05	0.30	0.20	1.71	0.50
11JY1385	C5 RIL 159	79	LP	1	27.27	39.20	1.21	0.72	0.13	0.09	1.44	0.22
11JY1387	C5 RIL 161	80	LP	1	35.77	47.20	2.24	1.35	0.20	0.13	1.32	0.33
11JY1388	C5 RIL 162	81	LP	1	37.45	45.10	1.64	1.40	0.17	0.14	1.20	0.31
11JY1389	C5 RIL 163	82	LP	1	44.05	53.10	3.26	2.59	0.32	0.30	1.21	0.62
11JY1391	C5 RIL 165	83	LP	1	40.00	41.20	2.53	1.40	0.29	0.20	1.03	0.49
11JY1394	C5 RIL 169	84	LP	1	31.80	40.00	1.26	0.60	0.15	0.12	1.26	0.27
11JY1396	C5 RIL 171	85	LP	1	41.60	44.10	3.54	2.36	0.34	0.23	1.06	0.57
11JY1397	C5 RIL 172	86	LP	1	32.50	45.73	1.78	1.20	0.16	0.12	1.41	0.28
11JY1399	C5 RIL 174	87	LP	1	36.40	35.20	3.46	1.95	0.38	0.32	0.97	0.70
11JY1400	C5 RIL 176	88	LP	1	38.50	40.67	3.05	2.25	0.34	0.25	1.06	0.59
11JY1401	C5 RIL 177	89	LP	1	37.65	50.80	3.51	2.78	0.36	0.27	1.35	0.63
11JY1402	C5 RIL 178	90	LP	1	34.50	42.60	2.42	2.26	0.31	0.20	1.23	0.51
11JY1404	C5 RIL 182	91	LP	1	30.30	39.60	2.37	1.86	0.26	0.25	1.31	0.51
11JY1405	C5 RIL 183	92	LP	1	32.33	37.03	2.62	2.07	0.29	0.22	1.15	0.51
11JY1406	C5 RIL 184	93	LP	1	29.80	54.15	1.57	1.28	0.20	0.14	1.82	0.34
11JY1409	C5 RIL 187	94	LP	1	29.00	37.70	1.75	1.93	0.22	0.20	1.30	0.42
11JY1410	C5 RIL 188	95	LP	1	29.35	37.10	1.84	1.57	0.20	0.17	1.26	0.37
11JY1412	C5 RIL 191	96	LP	1	38.33	71.00	4.03	3.30	0.46	0.33	1.85	0.79
11JY1413	C5 RIL 192	97	LP	1	31.70	44.45	1.78	1.24	0.23	0.17	1.40	0.40
11JY1414	C5 RIL 193	98	LP	1	33.30	44.95	2.13	1.59	0.26	0.22	1.35	0.48
11JY1416	C5 RIL 195	99	LP	1	28.00	32.30	2.00	1.08	0.27	0.23	1.15	0.50
11JY1418	C5 RIL 198	100	LP	1	27.40	51.60	1.82	1.78	0.30	0.26	1.88	0.56
11JY1420	C5 RIL 200	101	LP	1	32.00	32.90	2.50	1.90	0.28	0.20	1.03	0.48
11JY1421	C5 RIL 203	102	LP	1	32.75	44.80	2.15	1.82	0.23	0.17	1.37	0.40
11JY1422	C5 RIL 204	103	LP	1	33.60	43.75	2.37	2.20	0.26	0.20	1.30	0.46
11JY1423	C5 RIL 205	104	LP	1	32.75	58.85	1.92	1.19	0.30	0.24	1.80	0.54
11JY1424	C5 RIL 206	105	LP	1	38.87	58.67	2.87	2.77	0.26	0.21	1.51	0.47
11JY1425	C5 RIL 207	106	LP	1	30.40	40.70	2.21	1.99	0.40	0.38	1.34	0.78
11JY1426	C5 RIL 208	107	LP	1	43.60	47.40	3.64	2.26	0.42	0.40	1.09	0.82

Seed ID	Acc	Gen	trt.	rep	SL	RL	SFW	RFW	SDW	RDW	RSR	TDM
11JY1428	C5 RIL 210	108	LP	1	30.65	47.60	2.53	1.68	0.28	0.21	1.55	0.49
11JY1429	C5 RIL 211	109	LP	1	29.99	45.39	2.03	2.38	0.37	0.26	1.51	0.63
11JY1429	C5 RIL 211	110	LP	1	33.60	39.65	2.14	1.67	0.23	0.17	1.18	0.40
11JY1431	C5 RIL 214	111	LP	1	29.45	49.40	2.27	2.44	0.25	0.20	1.68	0.45
11JY1433	C5 RIL 216	112	LP	1	27.25	33.60	1.31	0.62	0.17	0.14	1.23	0.31
11JY1434	C5 RIL 217	113	LP	1	38.30	37.50	4.41	1.95	0.48	0.42	0.98	0.90
11JY1435	C5 RIL 218	114	LP	1	31.40	63.50	2.64	2.49	0.30	0.21	2.02	0.51
11JY1437	C5 RIL 220	115	LP	1	32.95	52.35	2.13	2.22	0.22	0.22	1.59	0.44
11JY1440	C5 RIL 225	116	LP	1	32.17	55.00	2.46	2.57	0.26	0.21	1.71	0.47
11JY1442	C5 RIL 227	117	LP	1	30.93	44.27	2.32	2.33	0.25	0.18	1.43	0.43
11JY1444	C5 RIL 229	118	LP	1	40.30	60.10	3.41	2.80	0.33	0.24	1.49	0.57
11JY1454	C5 RIL 240	119	LP	1	32.45	45.90	3.00	2.29	0.30	0.24	1.41	0.54
11JY1455	C5 RIL 241	120	LP	1	29.50	41.00	1.25	1.46	0.30	0.13	1.39	0.43
11JY1456	C5 RIL 243	121	LP	1	29.80	40.33	1.85	1.76	0.24	0.17	1.35	0.41
11JY1459	C5 RIL P2	122	LP	1	35.15	53.50	3.44	1.55	0.34	0.26	1.52	0.60
11JY1460	C5 RIL P1	123	LP	1	31.80	37.50	1.15	0.47	0.12	0.10	1.18	0.22
11JY1461	C6 RIL 3	124	LP	1	40.60	31.80	2.44	1.61	0.26	0.10	0.78	0.36
11JY1462	C6 RIL 14	125	LP	1	49.87	60.10	5.93	2.67	0.32	0.15	1.21	0.47
11JY1463	C6 RIL 23	126	LP	1	33.00	35.00	1.53	1.43	0.18	0.13	1.06	0.31
11JY1464	C6 RIL 24	127	LP	1	37.00	40.00	2.64	2.14	0.32	0.22	1.08	0.54
11JY1465	C6 RIL 25	128	LP	1	44.67	63.67	3.52	3.49	0.39	0.29	1.43	0.68
11JY1466	C6 RIL 29	129	LP	1	29.00	33.00	1.58	1.80	0.21	0.16	1.14	0.37
11JY1468	C6 RIL 144	130	LP	1	33.00	39.00	1.46	1.40	0.19	0.17	1.18	0.36
11JY1469	C6 RIL 203	131	LP	1	42.33	53.00	3.43	3.37	0.38	0.26	1.25	0.64
11JY1470	C6 RIL 212	132	LP	1	36.33	36.33	2.15	1.03	0.24	0.11	1.00	0.35
11JY1472	C6 RIL 219	133	LP	1	28.00	30.00	1.36	1.29	0.15	0.10	1.07	0.25
11JY1473	C6 RIL 239	134	LP	1	33.87	42.27	2.37	1.93	0.26	0.15	1.25	0.41
11JY1474	C6 RIL 243	135	LP	1	43.60	47.43	4.21	3.63	0.49	0.33	1.09	0.82
11JY1475	C6 RIL 272	136	LP	1	48.20	51.73	5.66	2.16	0.44	0.12	1.07	0.56
11JY1476	C6 RIL 273	137	LP	1	40.07	42.13	3.17	2.04	0.40	0.28	1.05	0.68
11JY1477	C6 RIL 283	138	LP	1	39.97	42.33	2.43	1.78	0.32	0.20	1.06	0.52
11JY1478	C6 RIL 293	139	LP	1	40.63	36.60	3.09	1.72	0.43	0.31	0.90	0.74
11JY1479	C6 RIL 298	140	LP	1	35.70	29.80	2.96	0.90	0.42	0.22	0.83	0.64
11JY1480	C6 RIL 303	141	LP	1	39.67	21.67	2.61	1.08	0.31	0.19	0.55	0.50
11JY1481	C6 RIL 327	142	LP	1	28.15	33.85	1.36	1.16	0.17	0.14	1.20	0.31
11JY1482	C6 RIL 341	143	LP	1	31.45	24.90	2.12	1.81	0.26	0.18	0.79	0.44

Seed ID	Acc	Gen	trt.	rep	SL	RL	SFW	RFW	SDW	RDW	RSR	TDM
11JY1483	C6 RIL 348	144	LP	1	19.80	34.70	1.14	1.05	0.16	0.13	1.75	0.29
11JY1484	C6 RIL 402	145	LP	1	41.55	34.60	4.34	3.39	0.44	0.34	0.83	0.78
11JY1485	C6 RIL 403	146	LP	1	32.83	31.67	2.18	1.37	0.23	0.15	0.96	0.38
11JY1487	C6 RIL 5	147	LP	1	48.67	22.23	3.49	0.49	0.31	0.09	0.46	0.40
11JY1488	C6 RIL 7	148	LP	1	41.40	49.50	4.92	3.11	0.51	0.41	1.20	0.92
11JY1489	C6 RIL 10	149	LP	1	40.20	45.70	3.78	2.39	0.44	0.34	1.14	0.78
11JY1490	C6 RIL 15	150	LP	1	45.90	49.10	4.61	4.03	0.50	0.36	1.07	0.86
11JY1491	C6 RIL 19	151	LP	1	40.43	46.90	2.98	3.24	0.35	0.26	1.16	0.61
11JY1493	C6 RIL 21	152	LP	1	39.83	44.77	3.30	3.09	0.35	0.27	1.12	0.62
11JY1494	C6 RIL 23	153	LP	1	45.50	55.60	3.32	3.83	0.37	0.33	1.22	0.70
11JY1495	C6 RIL 24	154	LP	1	39.60	37.30	3.17	2.95	0.37	0.28	0.94	0.65
11JY1496	C6 RIL 25	155	LP	1	37.50	38.87	2.21	2.52	0.30	0.21	1.04	0.51
11JY1497	C6 RIL 28	156	LP	1	26.00	28.50	1.00	0.59	0.11	0.08	1.10	0.19
11JY1498	C6 RIL 29	157	LP	1	43.43	52.60	4.17	4.45	0.46	0.32	1.21	0.78
11JY1499	C6 RIL 30	158	LP	1	42.85	32.07	3.72	2.09	0.42	0.16	0.75	0.58
11JY1500	C6 RIL 31	159	LP	1	35.93	38.73	2.54	1.98	0.33	0.26	1.08	0.59
11JY1501	C6 RIL 32	160	LP	1	42.07	49.83	2.67	1.89	0.34	0.22	1.18	0.56
11JY1502	C6 RIL 33	161	LP	1	35.10	29.20	2.40	1.17	0.31	0.17	0.83	0.48
11JY1503	C6 RIL 36	162	LP	1	43.90	32.33	2.71	3.17	0.24	0.21	0.74	0.45
11JY1504	C6 RIL 39	163	LP	1	36.03	44.37	2.76	1.88	0.34	0.26	1.23	0.60
11JY1505	C6 RIL 40	164	LP	1	36.60	39.60	2.63	1.63	0.29	0.21	1.08	0.50
11JY1506	C6 RIL 42	165	LP	1	36.80	34.23	2.96	2.48	0.31	0.21	0.93	0.52
11JY1508	C6 RIL 47	166	LP	1	39.40	47.40	2.79	1.77	0.34	0.19	1.20	0.53
11JY1509	C6 RIL 49	167	LP	1	29.80	32.50	1.75	1.00	0.21	0.13	1.09	0.34
11JY1510	C6 RIL 50	168	LP	1	33.53	44.07	2.48	1.78	0.27	0.22	1.31	0.49
11JY1511	C6 RIL 52	169	LP	1	38.77	25.47	2.78	2.02	0.28	0.15	0.66	0.43
11JY1512	C6 RIL 53	170	LP	1	39.05	45.35	2.85	1.59	0.31	0.17	1.16	0.48
11JY1513	C6 RIL 57	171	LP	1	33.25	38.75	2.14	1.33	0.24	0.16	1.17	0.40
11JY1514	C6 RIL 58	172	LP	1	53.60	25.60	6.93	4.52	0.84	0.50	0.48	1.34
11JY1515	C6 RIL 59	173	LP	1	34.70	39.70	1.93	1.09	0.24	0.14	1.14	0.38
11JY1516	C6 RIL 60	174	LP	1	31.00	46.05	1.62	1.46	0.22	0.18	1.49	0.40
11JY1517	C6 RIL 64	175	LP	1	37.73	34.67	2.17	1.25	0.24	0.16	0.92	0.40
11JY1518	C6 RIL 65	176	LP	1	37.30	49.07	2.30	1.71	0.30	0.22	1.32	0.52
11JY1519	C6 RIL 66	177	LP	1	28.95	38.90	1.42	1.22	0.19	0.16	1.34	0.35
11JY1520	C6 RIL 68	178	LP	1	23.15	29.50	0.67	0.28	0.13	0.08	1.27	0.21
11JY1521	C6 RIL 69	179	LP	1	29.57	36.37	1.57	1.02	0.20	0.15	1.23	0.35

Seed ID	Acc	Gen	trt.	rep	SL	RL	SFW	RFW	SDW	RDW	RSR	TDM
11JY1522	C6 RIL 70	180	LP	1	36.73	41.47	3.12	2.41	0.23	0.13	1.13	0.36
11JY1523	C6 RIL 72	181	LP	1	36.40	38.50	1.80	1.43	0.23	0.17	1.06	0.40
11JY1524	C6 RIL 74	182	LP	1	40.83	39.53	2.25	1.77	0.29	0.23	0.97	0.52
11JY1525	C6 RIL 76	183	LP	1	36.95	48.00	2.51	1.52	0.35	0.24	1.30	0.59
11JY1527	C6 RIL 79	184	LP	1	32.10	39.00	2.14	1.67	0.29	0.23	1.21	0.52
11JY1528	C6 RIL 83	185	LP	1	37.80	28.00	2.33	1.51	0.24	0.17	0.74	0.41
11JY1529	C6 RIL 85	186	LP	1	34.17	31.47	1.80	0.81	0.23	0.15	0.92	0.38
11JY1530	C6 RIL 86	187	LP	1	24.00	15.83	0.73	0.32	0.13	0.08	0.66	0.21
11JY1531	C6 RIL 91	188	LP	1	39.47	24.37	2.84	1.74	0.41	0.29	0.62	0.70
11JY1532	C6 RIL 94	189	LP	1	42.20	39.93	2.93	0.63	0.32	0.14	0.95	0.46
11JY1533	C6 RIL 95	190	LP	1	37.40	46.67	1.99	0.73	0.27	0.19	1.25	0.46
11JY1534	C6 RIL 97	191	LP	1	37.37	55.87	4.19	3.18	0.32	0.23	1.50	0.55
11JY1535	C6 RIL 103	192	LP	1	37.80	57.05	1.84	0.70	0.23	0.15	1.51	0.38
11JY1536	C6 RIL 104	193	LP	1	29.45	36.25	1.38	0.49	0.17	0.09	1.23	0.26
11JY1537	C6 RIL 107	194	LP	1	38.27	36.53	1.97	0.72	0.27	0.18	0.95	0.45
11JY1538	C6 RIL 108	195	LP	1	45.57	29.50	2.87	0.79	0.38	0.20	0.65	0.58
11JY1539	C6 RIL 109	196	LP	1	46.65	30.50	1.88	0.90	0.25	0.15	0.65	0.40
11JY1540	C6 RIL 112	197	LP	1	30.10	30.17	1.20	0.49	0.15	0.11	1.00	0.26
11JY1541	C6 RIL 113	198	LP	1	45.50	37.17	3.52	2.00	0.36	0.29	0.82	0.65
11JY1542	C6 RIL 115	199	LP	1	41.57	24.67	2.33	1.02	0.31	0.18	0.59	0.49
11JY1543	C6 RIL 116	200	LP	1	44.73	49.90	2.18	0.60	0.27	0.15	1.12	0.42
11JY1544	C6 RIL 117	201	LP	1	42.40	48.80	2.13	0.85	0.23	0.17	1.15	0.40
11JY1545	C6 RIL 120	202	LP	1	50.45	59.45	4.26	2.53	0.38	0.19	1.18	0.57
11JY1546	C6 RIL 121	203	LP	1	40.50	31.43	1.97	0.61	0.30	0.18	0.78	0.48
11JY1547	C6 RIL 123	204	LP	1	42.57	37.67	2.66	0.97	0.36	0.22	0.88	0.58
11JY1548	C6 RIL 124	205	LP	1	37.30	29.37	1.23	0.35	0.21	0.12	0.79	0.33
11JY1549	C6 RIL 127	206	LP	1	40.73	50.73	3.08	1.72	0.35	0.18	1.25	0.53
11JY1550	C6 RIL 131	207	LP	1	31.30	40.20	1.30	0.51	0.17	0.13	1.28	0.30
11JY1551	C6 RIL 134	208	LP	1	48.85	37.65	2.97	1.45	0.29	0.18	0.77	0.47
11JY1552	C6 RIL 135	209	LP	1	46.60	53.40	3.26	1.74	0.39	0.23	1.15	0.62
11JY1553	C6 RIL 138	210	LP	1	45.63	47.40	3.22	1.18	0.41	0.22	1.04	0.63
11JY1554	C6 RIL 139	211	LP	1	33.00	39.90	1.72	1.12	0.19	0.15	1.21	0.34
11JY1555	C6 RIL 140	212	LP	1	23.00	30.20	0.64	0.27	0.09	0.05	1.31	0.14
11JY1556	C6 RIL 141	213	LP	1	44.00	50.87	3.84	1.84	0.34	0.45	1.16	0.79
11JY1557	C6 RIL 142	214	LP	1	47.13	50.27	4.20	2.36	0.49	0.33	1.07	0.82
11JY1558	C6 RIL 143	215	LP	1	36.20	49.30	1.81	1.45	0.20	0.12	1.36	0.32

Seed ID	Acc	Gen	trt.	rep	SL	RL	SFW	RFW	SDW	RDW	RSR	TDM
11JY1560	C6 RIL 146	216	LP	1	43.53	41.17	2.63	1.65	0.25	0.14	0.95	0.39
11JY1561	C6 RIL 147	217	LP	1	21.20	25.50	0.71	0.50	0.07	0.06	1.20	0.13
11JY1562	C6 RIL 148	218	LP	1	33.35	41.80	2.06	1.94	0.21	0.17	1.25	0.38
11JY1563	C6 RIL 150	219	LP	1	24.10	29.50	1.23	1.15	0.12	0.09	1.22	0.21
11JY1565	C6 RIL 153	220	LP	1	40.55	45.20	3.97	3.31	0.45	0.26	1.11	0.71
11JY1566	C6 RIL 156	221	LP	1	40.50	60.30	2.64	2.93	0.36	0.32	1.49	0.68
11JY1567	C6 RIL 160	222	LP	1	43.47	36.97	3.07	1.01	0.33	0.16	0.85	0.49
11JY1568	C6 RIL 161	223	LP	1	37.73	44.07	2.20	1.35	0.23	0.17	1.17	0.40
11JY1569	C6 RIL 162	224	LP	1	56.10	34.48	5.51	4.36	0.56	0.33	0.61	0.89
11JY1570	C6 RIL 163	225	LP	1	35.63	40.43	1.97	1.11	0.23	0.17	1.13	0.40
11JY1571	C6 RIL 164	226	LP	1	36.55	35.10	1.84	0.69	0.23	0.12	0.96	0.35
11JY1572	C6 RIL 169	227	LP	1	40.10	35.87	2.11	0.86	0.26	0.14	0.89	0.40
11JY1573	C6 RIL 171	228	LP	1	45.80	31.05	2.75	1.48	0.34	0.18	0.68	0.52
11JY1574	C6 RIL 172	229	LP	1	38.00	40.85	2.12	1.46	0.29	0.25	1.08	0.54
11JY1576	C6 RIL 174	230	LP	1	49.50	42.70	2.67	0.92	0.33	0.17	0.86	0.50
11JY1577	C6 RIL 175	231	LP	1	30.50	35.20	1.48	0.72	0.17	0.10	1.15	0.27
11JY1578	C6 RIL 176	232	LP	1	45.40	48.60	2.75	2.01	0.27	0.27	1.07	0.54
11JY1579	C6 RIL 179	233	LP	1	36.80	32.70	1.69	0.76	0.20	0.14	0.89	0.34
11JY1580	C6 RIL 181	234	LP	1	37.10	28.05	1.89	0.58	0.24	0.13	0.76	0.37
11JY1581	C6 RIL 184	235	LP	1	33.40	27.30	2.70	1.41	0.08	0.04	0.82	0.12
11JY1582	C6 RIL 186	236	LP	1	38.80	35.83	3.82	3.54	0.40	0.26	0.92	0.66
11JY1583	C6 RIL 187	237	LP	1	29.63	39.75	1.38	0.46	0.18	0.13	1.34	0.31
11JY1584	C6 RIL 188	238	LP	1	36.33	48.67	2.14	0.66	0.26	0.15	1.34	0.41
11JY1585	C6 RIL 189	239	LP	1	21.10	25.50	0.82	0.49	0.13	0.10	1.21	0.23
11JY1586	C6 RIL 190	240	LP	1	46.60	37.77	3.49	1.17	0.29	0.16	0.81	0.45
11JY1587	C6 RIL 192	241	LP	1	39.25	30.70	2.08	0.67	0.22	0.15	0.78	0.37
11JY1588	C6 RIL 193	242	LP	1	43.70	46.47	2.73	0.76	0.31	0.21	1.06	0.52
11JY1589	C6 RIL 203	243	LP	1	47.53	52.27	2.79	0.76	0.28	0.17	1.10	0.45
11JY1590	C6 RIL 204	244	LP	1	42.63	46.73	2.12	0.68	0.27	0.18	1.10	0.45
11JY1591	C6 RIL 205	245	LP	1	33.15	34.60	1.04	0.24	0.14	0.10	1.04	0.24
11JY1592	C6 RIL 206	246	LP	1	42.20	50.25	1.96	0.82	0.21	0.14	1.19	0.35
11JY1593	C6 RIL 207	247	LP	1	36.10	44.37	3.30	2.45	0.35	0.17	1.23	0.52
11JY1594	C6 RIL 211	248	LP	1	35.80	25.10	1.41	0.76	0.17	0.12	0.70	0.29
11JY1595	C6 RIL 212	249	LP	1	41.93	43.27	2.49	1.17	0.25	0.15	1.03	0.40
11JY1596	C6 RIL 217	250	LP	1	36.00	24.20	1.52	0.50	0.18	0.11	0.67	0.29
11JY1597	C6 RIL 218	251	LP	1	43.55	35.75	2.55	0.88	0.23	0.12	0.82	0.35

Seed ID	Acc	Gen	trt.	rep	SL	RL	SFW	RFW	SDW	RDW	RSR	TDM
11JY1598	C6 RIL 219	252	LP	1	53.90	49.10	4.63	1.57	0.45	0.19	0.91	0.64
11JY1599	C6 RIL 220	253	LP	1	37.55	48.35	2.33	0.82	0.30	0.24	1.29	0.54
11JY1600	C6 RIL 225	254	LP	1	42.67	33.80	3.53	2.21	0.34	0.18	0.79	0.52
11JY1601	C6 RIL 230	255	LP	1	37.97	57.67	2.52	2.36	0.28	0.21	1.52	0.49
11JY1602	C6 RIL 231	256	LP	1	48.90	32.37	3.96	2.99	0.31	0.22	0.66	0.53
11JY1603	C6 RIL 232	257	LP	1	46.13	44.03	3.34	2.50	0.37	0.22	0.95	0.59
11JY1604	C6 RIL 234	258	LP	1	42.03	64.20	3.24	3.69	0.37	0.26	1.53	0.63
11JY1605	C6 RIL 235	259	LP	1	44.07	51.30	3.00	3.17	0.34	0.22	1.16	0.56
11JY1606	C6 RIL 236	260	LP	1	35.00	32.43	2.82	2.54	0.31	0.19	0.93	0.50
11JY1607	C6 RIL 238	261	LP	1	42.53	40.73	3.96	2.76	0.41	0.23	0.96	0.64
11JY1608	C6 RIL 239	262	LP	1	39.65	31.10	1.68	1.00	0.17	0.07	0.78	0.24
11JY1609	C6 RIL 241	263	LP	1	45.83	59.30	3.02	3.14	0.34	0.24	1.29	0.58
11JY1610	C6 RIL 243	264	LP	1	49.93	77.77	4.66	4.75	0.48	0.36	1.56	0.84
11JY1611	C6 RIL 245	265	LP	1	34.50	35.27	2.22	1.41	0.25	0.10	1.02	0.35
11JY1612	C6 RIL 249	266	LP	1	42.37	39.70	3.31	1.97	0.36	0.16	0.94	0.52
11JY1613	C6 RIL 253	267	LP	1	47.37	55.67	4.39	3.26	0.47	0.25	1.18	0.72
11JY1614	C6 RIL 258	268	LP	1	40.25	43.60	2.77	1.89	0.30	0.16	1.08	0.46
11JY1616	C6 RIL 261	269	LP	1	47.67	40.70	5.50	3.35	0.53	0.25	0.85	0.78
11JY1617	C6 RIL 262	270	LP	1	37.67	24.03	2.43	1.38	0.32	0.15	0.64	0.47
11JY1619	C6 RIL 265	271	LP	1	48.47	47.77	4.29	2.70	0.47	0.23	0.99	0.70
11JY1620	C6 RIL 267	272	LP	1	41.27	45.07	3.51	2.11	0.34	0.15	1.09	0.49
11JY1621	C6 RIL 268	273	LP	1	46.47	36.90	4.26	3.25	0.40	0.23	0.79	0.63
11JY1622	C6 RIL 270	274	LP	1	42.43	41.83	2.81	2.58	0.32	0.21	0.99	0.53
11JY1623	C6 RIL 272	275	LP	1	43.60	53.73	3.21	2.49	0.34	0.21	1.23	0.55
11JY1624	C6 RIL 273	276	LP	1	40.90	44.83	3.36	1.71	0.32	0.13	1.10	0.45
11JY1625	C6 RIL 275	277	LP	1	44.07	31.17	4.59	2.79	0.45	0.25	0.71	0.70
11JY1626	C6 RIL 276	278	LP	1	39.30	37.60	3.10	2.63	0.37	0.23	0.96	0.60
11JY1627	C6 RIL 282	279	LP	1	49.13	44.77	5.02	3.31	0.61	0.32	0.91	0.93
11JY1628	C6 RIL 283	280	LP	1	52.37	46.37	4.31	3.71	0.51	0.28	0.89	0.79
11JY1629	C6 RIL 284	281	LP	1	48.50	36.70	4.91	3.30	0.46	0.25	0.76	0.71
11JY1630	C6 RIL 286	282	LP	1	32.53	36.20	2.09	2.10	0.26	0.17	1.11	0.43
11JY1631	C6 RIL 287	283	LP	1	40.53	45.07	2.90	2.74	0.35	0.22	1.11	0.57
11JY1632	C6 RIL 288	284	LP	1	29.50	24.20	1.45	0.66	0.11	0.05	0.82	0.16
11JY1633	C6 RIL 289	285	LP	1	44.83	41.87	3.73	2.40	0.40	0.21	0.93	0.61
11JY1634	C6 RIL 291	286	LP	1	43.93	52.90	3.06	2.31	0.35	0.18	1.20	0.53
11JY1635	C6 RIL 292	287	LP	1	32.30	10.30	1.43	1.18	0.20	0.13	0.32	0.33

Seed ID	Acc	Gen	trt.	rep	SL	RL	SFW	RFW	SDW	RDW	RSR	TDM
11JY1636	C6 RIL 293	288	LP	1	50.80	57.57	4.22	3.35	0.50	0.29	1.13	0.79
11JY1637	C6 RIL 295	289	LP	1	42.50	32.15	3.87	1.90	0.41	0.19	0.76	0.60
11JY1638	C6 RIL 297	290	LP	1	44.43	32.10	3.42	2.22	0.37	0.20	0.72	0.57
11JY1639	C6 RIL 298	291	LP	1	34.27	36.07	2.67	1.95	0.27	0.14	1.05	0.41
11JY1640	C6 RIL 300	292	LP	1	40.13	33.37	3.44	1.83	0.33	0.15	0.83	0.48
11JY1641	C6 RIL 303	293	LP	1	36.23	39.20	2.48	2.06	0.22	0.13	1.08	0.35
11JY1642	C6 RIL 310	294	LP	1	43.07	40.17	3.56	3.28	0.35	0.24	0.93	0.59
11JY1643	C6 RIL 314	295	LP	1	34.35	33.30	1.64	0.55	0.14	0.50	0.97	0.64
11JY1644	C6 RIL 315	296	LP	1	47.63	36.40	4.27	2.98	0.40	0.23	0.76	0.63
11JY1645	C6 RIL 316	297	LP	1	25.40	42.20	1.46	0.82	0.11	0.07	1.66	0.18
11JY1646	C6 RIL 317	298	LP	1	36.63	43.93	2.55	2.57	0.29	0.16	1.20	0.45
11JY1647	C6 RIL 318	299	LP	1	45.27	23.00	4.62	3.56	0.43	0.28	0.51	0.71
11JY1648	C6 RIL 319	300	LP	1	33.00	30.00	2.46	1.76	0.26	0.13	0.91	0.39
11JY1649	C6 RIL 320	301	LP	1	40.10	41.17	2.93	2.94	0.32	0.21	1.03	0.53
11JY1650	C6 RIL 321	302	LP	1	27.50	32.43	1.49	0.58	0.23	0.12	1.18	0.35
11JY1651	C6 RIL 322	303	LP	1	38.13	40.60	4.44	2.64	0.51	0.30	1.06	0.81
11JY1652	C6 RIL 324	304	LP	1	50.73	43.60	4.82	2.96	0.45	0.23	0.86	0.68
11JY1653	C6 RIL 327	305	LP	1	57.10	51.60	5.66	4.43	0.53	0.32	0.90	0.85
11JY1654	C6 RIL 328	306	LP	1	46.83	48.90	4.87	2.96	0.45	0.22	1.04	0.67
11JY1655	C6 RIL 330	307	LP	1	45.73	41.03	3.84	2.08	0.37	0.16	0.90	0.53
11JY1656	C6 RIL 332	308	LP	1	54.30	66.07	6.01	4.75	0.53	0.34	1.22	0.87
11JY1657	C6 RIL 333	309	LP	1	47.87	33.00	3.46	2.32	0.33	0.19	0.69	0.52
11JY1658	C6 RIL 335	310	LP	1	35.00	37.00	1.75	2.19	0.26	0.19	1.06	0.45
11JY1659	C6 RIL 336	311	LP	1	30.50	25.00	1.37	1.32	0.21	0.13	0.82	0.34
11JY1660	C6 RIL 339	312	LP	1	31.67	43.00	2.06	1.89	0.21	0.15	1.36	0.36
11JY1661	C6 RIL 340	313	LP	1	27.50	28.50	1.08	1.29	0.16	0.12	1.04	0.28
11JY1662	C6 RIL 341	314	LP	1	32.50	36.25	1.64	1.81	0.25	0.20	1.12	0.45
11JY1663	C6 RIL 342	315	LP	1	34.33	60.33	2.29	2.11	0.25	0.17	1.76	0.42
11JY1664	C6 RIL 344	316	LP	1	32.33	29.00	1.89	1.59	0.20	0.16	0.90	0.36
11JY1665	C6 RIL 346	317	LP	1	36.47	33.07	3.75	2.01	0.36	0.17	0.91	0.53
11JY1666	C6 RIL 347	318	LP	1	39.33	55.33	3.72	2.38	0.33	0.17	1.41	0.50
11JY1667	C6 RIL 349	319	LP	1	43.50	63.00	2.34	2.51	0.30	0.22	1.45	0.52
11JY1668	C6 RIL 352	320	LP	1	36.00	53.00	2.00	1.86	0.24	0.15	1.47	0.39
11JY1669	C6 RIL 354	321	LP	1	33.75	57.00	1.97	1.18	0.21	0.11	1.69	0.32
11JY1670	C6 RIL 355	322	LP	1	32.67	41.67	1.76	1.61	0.26	0.17	1.28	0.43
11JY1671	C6 RIL 356	323	LP	1	36.00	49.67	2.25	1.80	0.28	0.15	1.38	0.43

Seed ID	Acc	Gen	trt.	rep	SL	RL	SFW	RFW	SDW	RDW	RSR	TDM
11JY1672	C6 RIL 358	324	LP	1	38.00	59.00	2.53	2.70	0.26	0.20	1.55	0.46
11JY1673	C6 RIL 361	325	LP	1	25.00	41.67	1.18	1.57	0.15	0.10	1.67	0.25
11JY1674	C6 RIL 362	326	LP	1	39.00	43.67	3.32	2.62	0.34	0.20	1.12	0.54
11JY1675	C6 RIL 364	327	LP	1	42.33	47.33	3.07	2.31	0.34	0.18	1.12	0.52
11JY1676	C6 RIL 366	328	LP	1	36.93	28.53	2.70	1.84	0.28	0.13	0.77	0.41
11JY1677	C6 RIL 368	329	LP	1	36.17	37.00	1.88	1.19	0.22	0.12	1.02	0.34
11JY1678	C6 RIL 372	330	LP	1	49.33	53.33	4.01	2.23	0.41	0.19	1.08	0.60
11JY1680	C6 RIL 379	331	LP	1	32.50	33.50	1.28	1.35	0.16	0.10	1.03	0.26
11JY1682	C6 RIL 382	332	LP	1	39.33	45.67	2.85	2.09	0.31	0.20	1.16	0.51
11JY1683	C6 RIL 388	333	LP	1	41.00	44.67	3.40	2.89	0.37	0.27	1.09	0.64
11JY1684	C6 RIL 391	334	LP	1	31.00	36.00	1.89	2.01	0.20	0.14	1.16	0.34
11JY1686	C6 RIL 394	335	LP	1	37.67	51.67	3.15	2.95	0.33	0.22	1.37	0.55
11JY1687	C6 RIL 395	336	LP	1	43.00	44.67	3.59	4.35	0.38	0.22	1.04	0.60
11JY1688	C6 RIL 398	337	LP	1	40.33	40.33	3.52	3.17	0.35	0.24	1.00	0.59
11JY1689	C6 RIL 400	338	LP	1	34.67	26.00	1.60	1.24	0.18	0.11	0.75	0.29
11JY2045	(CUBA/GUAD C1 F27-4-3-3-B-1-Bx[KILIMA ST94A]-30/MSV-03-2-10-B-2-B-B)-160-1-B-3-B	339	LP	1	35.08	47.39	2.28	1.37	0.25	0.18	1.35	0.43
11JY2047	[[MSRXPOOL9]C1F2-176-4-7-X-1-B/CML206]-5-2-3-1-BBBBB-B-B-B	340	LP	1	44.41	54.39	4.24	2.85	0.47	0.30	1.22	0.77
11JY2051	[CML199/[EV7992#/EV8449-SR]C1F2-334-1(OSU8i)-6-3-Sn]-B-23-2-2-B*4-B-B-B	341	LP	1	31.58	40.81	1.80	2.01	0.36	0.16	1.29	0.52
11JY2052	[CML312/[TUXPSEQ]C1F2/P49-SR]F2-45-3-2-1-BB//INTA-F2-192-2-1-1-1-BBBB]-1-5-1-1-1-BBB-B-B-B	342	LP	1	36.08	35.73	2.87	1.74	0.33	0.18	0.99	0.51
11JY2053	[CML312/CML445//[TUXPSEQ]C1F2/P49-SR]F2-45-3-2-1-BBB]-1-2-1-1-2-BBB-B-B-B	343	LP	1	27.58	33.81	1.35	0.83	0.18	0.07	1.23	0.25
11JY2054	[CML312/MAS[MSR/312]-109-3]-B-71-3-BBB-B-B-B	344	LP	1	40.74	48.73	3.18	1.89	0.36	0.17	1.20	0.52
11JY2055	[CML389/CML176]-B-29-2-2-B*5	345	LP	1	37.08	44.73	3.47	2.29	0.35	0.21	1.21	0.55
11JY2056	[CML395/CML440//[LPSC3H144-1-2-2-2-4-#-BB/SC/ZM605#b-19-2-X]-1-2-X-1-1-BB]-1-2-1-1-B]-3-2-1-1-BBB-B-B-B	346	LP	1	36.74	55.89	3.17	2.48	0.34	0.23	1.52	0.57
11JY2060	[CML444/ZSR923S4BULK-2-2-X-X-X-X-1-BB]-1-1-1-2/CML441]-1-1-1-2-BBB-B-B-B	347	LP	1	35.58	39.81	2.19	1.21	0.32	0.18	1.12	0.50
11JY2061	[DRB-F2-180-2/DRB-3-4-1]-X-6-1-3-BB-2-BBBBBB-B-B-B	348	LP	1	29.58	50.48	2.06	1.95	0.29	0.15	1.71	0.44

Seed ID	Acc	Gen	trt.	rep	SL	RL	SFW	RFW	SDW	RDW	RSR	TDM
11JY2063	[DTPWC8F31-4-2-1-6-B2/CML395//[CML445/ZM621B]-2-1-2-3-1-BB]-3-2-1-1-1-2-B-B-B	349	LP	1	42.74	50.39	3.23	1.85	0.33	0.21	1.18	0.54
11JY2064	[Ent320:92SEW2-77/[DMRESR-W]EarlySel-#I-2-4-B/CML386]-B-11-3-B-2-#-B*4	350	LP	1	28.91	60.48	1.69	2.17	0.25	0.15	2.09	0.40
11JY2065	[LZ956441/LZ966205]-B-3-4-4-B-5-BBBBB-B-B-B	351	LP	1	39.24	67.81	3.50	4.05	0.46	0.30	1.73	0.75
11JY2066	[MSRXPOOL9]C1F2-205-1(OSU23i)-5-3-X-X-1-B//EV7992/EV8449...-3-2-2-1-BBBBB-B-B-B	352	LP	1	34.24	55.48	3.61	2.30	0.42	0.21	1.62	0.62
11JY2067	[SYN-USAB2/SYN-ELIB2]-12-1-1-1-B*4-B-B-B	353	LP	1	45.08	63.73	4.36	3.10	0.54	0.25	1.41	0.80
11JY2070	02SADVE2B-#-42-1-1-1-1-B-B-B	354	LP	1	31.24	65.81	2.55	1.95	0.28	0.15	2.11	0.43
11JY2071	02SADVL2B-#-16-2-1-B-B-B	355	LP	1	30.24	47.14	1.36	1.44	0.24	0.18	1.56	0.42
11JY2075	20V-18	356	LP	1	34.91	35.48	2.71	1.46	0.50	0.22	1.02	0.71
11JY2076	622016-ZCN-2	357	LP	1	45.91	40.81	4.19	2.84	0.44	0.24	0.89	0.68
11JY2078	761BB2 BCox751B-B-1-1-B-B-B-B-B-B	358	LP	1	44.24	42.89	4.17	3.49	0.52	0.36	0.97	0.87
11JY2079	BRAZ 2309	359	LP	1	38.41	39.39	2.65	1.79	0.28	0.16	1.03	0.44
11JY2081	CL-04934 (P49C2H12-5-4xP23C2-11-1)-2-2-2-B*10	360	LP	1	33.74	34.73	3.39	2.71	0.36	0.24	1.03	0.60
11JY2083	CML103	361	LP	1	41.91	56.48	3.37	2.53	0.46	0.31	1.35	0.78
11JY2085	CML114	362	LP	1	44.74	58.39	4.15	4.09	0.46	0.37	1.31	0.83
11JY2086	CML115	363	LP	1	37.74	41.39	3.07	2.57	0.31	0.21	1.10	0.53
11JY2087	CML116	364	LP	1	37.58	46.48	2.73	2.32	0.30	0.20	1.24	0.50
11JY2088	CML118	365	LP	1	37.91	49.48	3.65	2.81	0.41	0.23	1.31	0.64
11JY2090	CML127	366	LP	1	38.58	55.81	3.31	2.91	0.38	0.18	1.45	0.55
11JY2091	CML130	367	LP	1	32.91	47.48	1.27	0.92	0.17	0.07	1.44	0.23
11JY2092	CML133	368	LP	1	40.58	65.14	3.51	4.69	0.58	0.33	1.61	0.91
11JY2093	CML134	369	LP	1	32.24	39.14	1.47	1.03	0.26	0.13	1.21	0.38
11JY2094	CML135	370	LP	1	33.24	46.48	3.45	3.98	0.45	0.33	1.40	0.78
11JY2102	CML169	371	LP	1	40.08	42.01	2.86	1.84	0.32	0.14	1.05	0.46
11JY2103	CML170	372	LP	1	43.24	47.34	3.74	3.38	0.40	0.25	1.09	0.66
11JY2107	CML192	373	LP	1	48.58	45.68	5.50	2.85	0.52	0.25	0.94	0.78
11JY2108	CML20	374	LP	1	44.08	40.39	3.46	2.00	0.37	0.21	0.92	0.58
11JY2109	CML202	375	LP	1	39.91	51.34	4.19	3.12	0.41	0.26	1.29	0.67
11JY2110	CML206	376	LP	1	45.24	56.01	4.97	3.22	0.51	0.28	1.24	0.80
11JY2112	CML226	377	LP	1	31.91	41.68	2.24	1.52	0.27	0.12	1.31	0.40
11JY2114	CML229	378	LP	1	43.41	39.73	4.26	2.46	0.43	0.21	0.92	0.64
11JY2117	CML283	379	LP	1	40.00	36.68	2.87	1.81	0.29	0.15	0.92	0.44
11JY2122	CML290	380	LP	1	32.24	38.39	2.53	1.77	0.24	0.17	1.19	0.41

Seed ID	Acc	Gen	trt.	rep	SL	RL	SFW	RFW	SDW	RDW	RSR	TDM
11JY2125	CML304	381	LP	1	35.58	39.68	3.56	3.07	0.36	0.22	1.12	0.58
11JY2126	CML31	382	LP	1	34.24	50.01	2.55	1.72	0.30	0.14	1.46	0.44
11JY2127	CML311/MBR C3 BC F23-1-2-1-B-B-B	383	LP	1	41.91	48.34	4.33	3.02	0.44	0.21	1.15	0.65
11JY2128	CML311/MBR C3 BC F3-1-1-1-B-B-B	384	LP	1	41.91	45.68	3.91	2.39	0.41	0.23	1.09	0.65
11JY2129	CML311/MBR C3 BC F3-1-1-2-B-B	385	LP	1	45.41	45.73	3.69	2.55	0.41	0.25	1.01	0.66
11JY2132	CML311/MBR C3 BC F43-2-1-1-B-B-B	386	LP	1	35.58	50.68	2.89	2.16	0.28	0.15	1.42	0.43
11JY2133	CML311/MBR C3 BC F65-1-2-2-B-B-B	387	LP	1	39.91	62.68	3.47	2.83	0.34	0.21	1.57	0.55
11JY2134	CML311/MBR C3 BC F95-2-2-1-B-B-B	388	LP	1	44.58	61.68	4.82	4.78	0.48	0.38	1.38	0.87
11JY2136	CML312SR	389	LP	1	40.58	55.01	3.63	2.38	0.35	0.19	1.36	0.54
11JY2137	CML312SRQ=[[(CLQ-RCWQ83xCML312SR)xCML312SR]xCML312SR)]-15-1-BBB	390	LP	1	35.58	33.34	2.98	2.17	0.38	0.17	0.94	0.55
11JY2140	CML322	391	LP	1	37.24	44.68	4.47	3.69	0.47	0.24	1.20	0.71
11JY2141	CML323	392	LP	1	54.91	44.68	6.22	3.61	0.63	0.29	0.81	0.92
11JY2142	CML325	393	LP	1	40.24	64.68	4.19	3.06	0.45	0.31	1.61	0.77
11JY2144	CML328	394	LP	1	48.24	62.68	5.45	3.57	0.54	0.29	1.30	0.84
11JY2146	CML338	395	LP	1	45.24	57.81	5.06	3.87	0.52	0.33	1.28	0.85
11JY2147	CML360	396	LP	1	45.91	46.48	4.81	3.19	0.49	0.27	1.01	0.76
11JY2148	CML361	397	LP	1	41.24	49.81	3.66	2.78	0.35	0.23	1.21	0.57
11JY2149	CML364	398	LP	1	46.01	47.31	3.85	2.64	0.45	0.22	1.03	0.67
11JY2150	CML380xMBR/MDR C3 BC F21-1-1-2-B-B-B-B-3-1-B-B-B	399	LP	1	29.24	60.48	3.17	2.63	0.32	0.20	2.07	0.52
11JY2151	CML384xMBR/MDR C3 BC F58-2-1-3-B-B-B-B-3-1-B-B-B	400	LP	1	41.34	40.64	4.57	2.69	0.51	0.23	0.98	0.74
11JY2152	CML389	401	LP	1	43.68	53.64	5.04	3.52	0.43	0.26	1.23	0.69
11JY2153	CML389/CML144//CML159//POOL15QPMSR-B-6-B-B	402	LP	1	41.01	43.98	3.71	2.56	0.41	0.22	1.07	0.63
11JY2154	CML40	403	LP	1	43.34	63.98	4.06	3.33	0.42	0.25	1.48	0.67
11JY2155	CML402	404	LP	1	51.01	55.31	7.41	3.90	0.78	0.30	1.08	1.08
11JY2157	CML411	405	LP	1	43.34	50.64	4.75	2.61	0.41	0.25	1.17	0.66
11JY2160	CML423	406	LP	1	43.01	54.98	4.53	2.87	0.39	0.25	1.28	0.65
11JY2162	CML428	407	LP	1	45.01	52.98	4.49	3.01	0.48	0.25	1.18	0.73
11JY2163	CML430	408	LP	1	42.84	55.64	5.52	2.85	0.41	0.22	1.30	0.63
11JY2164	CML431	409	LP	1	36.68	40.64	3.05	1.62	0.27	0.13	1.11	0.40
11JY2165	CML432	410	LP	1	39.34	38.64	4.53	2.15	0.40	0.19	0.98	0.58
11JY2166	CML433	411	LP	1	37.34	42.14	4.31	2.66	0.42	0.21	1.13	0.63

Seed ID	Acc	Gen	trt.	rep	SL	RL	SFW	RFW	SDW	RDW	RSR	TDM
11JY2168	CML445/CML144//CML159//POOL15QPMSR-B-55-B-B	412	LP	1	42.34	53.98	4.77	2.98	0.52	0.29	1.27	0.81
11JY2170	CML454	413	LP	1	40.34	69.98	3.13	3.13	0.32	0.26	1.73	0.58
11JY2172	CML468	414	LP	1	45.01	45.31	3.10	2.20	0.30	0.17	1.01	0.47
11JY2173	CML470	415	LP	1	46.01	45.31	4.49	3.00	0.43	0.31	0.98	0.74
11JY2176	CML479	416	LP	1	29.68	60.31	2.89	2.48	0.31	0.22	2.03	0.53
11JY2177	CML480	417	LP	1	38.68	46.64	3.17	1.86	0.32	0.22	1.21	0.54
11JY2180	CML496	418	LP	1	30.24	40.14	2.53	1.56	0.23	0.14	1.33	0.37
11JY2189	CML80	419	LP	1	52.68	62.64	6.58	4.24	0.54	0.36	1.19	0.90
11JY2192	CML94	420	LP	1	50.34	59.64	5.52	4.19	0.54	0.42	1.18	0.96
11JY2193	CML96	421	LP	1	45.34	42.31	4.66	2.25	0.40	0.23	0.93	0.63
11JY2194	CML99	422	LP	1	38.01	44.64	4.13	2.58	0.42	0.27	1.17	0.69
11JY2196	Cuba/GuadC3F125-2-2-1-B-B-B	423	LP	1	42.08	43.39	4.49	3.01	0.43	0.25	1.03	0.68
11JY2197	CY9169	424	LP	1	43.38	44.47	4.12	2.20	0.41	0.19	1.03	0.60
11JY2198	DTPW C9	425	LP	1	33.22	44.81	3.69	3.04	0.46	0.29	1.35	0.76
11JY2199	DTPWC9-F104-5-4-1-1-B-B-B	426	LP	1	42.22	54.81	4.01	2.78	0.47	0.23	1.30	0.70
11JY2200	DTPY C9	427	LP	1	40.24	55.81	4.61	3.62	0.47	0.33	1.39	0.81
11JY2201	DTPYC9-F46-1-2-1-2-B-B	428	LP	1	44.38	50.14	3.75	2.99	0.46	0.24	1.13	0.70
11JY2202	DTPYC9-F46-3-9-1-1-B-BTL-07B 6614-42	429	LP	1	31.22	40.81	2.11	1.27	0.27	0.11	1.31	0.38
11JY2204	Guad 6	430	LP	1	44.05	47.47	4.02	3.11	0.48	0.28	1.08	0.77
11JY2205	H-16	431	LP	1	42.05	58.14	4.39	3.87	0.48	0.28	1.38	0.75
11JY2207	La Posta Seq C7-F125-2-1-1-2-B-B-B	432	LP	1	32.38	48.47	3.41	2.73	0.36	0.24	1.50	0.60
11JY2211	La Posta Seq C7-F64-2-6-1-2-B-B-B	433	LP	1	43.91	54.14	4.23	3.68	0.42	0.32	1.23	0.74
11JY2216	La Posta Seq C7-F96-1-2-1-2-B-B	434	LP	1	41.91	66.81	3.86	2.61	0.36	0.21	1.59	0.57
11JY2218	LPSC7	435	LP	1	37.38	57.14	3.49	3.14	0.36	0.23	1.53	0.59
11JY2220	MAS[206/312]-23-2-1-1-B*6-B-B-B	436	LP	1	45.72	49.14	3.43	2.41	0.37	0.19	1.07	0.56
11JY2223	MBR C6 BC F234-1-B-#-1-1-B-B-B-B-B	437	LP	1	39.38	47.47	3.80	2.41	0.42	0.22	1.21	0.64
11JY2225	P402c2F2-695-2-BB-2-B*4-1-B	438	LP	1	34.72	43.81	2.75	2.15	0.31	0.17	1.26	0.48
11JY2228	P591c4 F55-2-2-2-B-B-B	439	LP	1	49.72	38.81	4.17	2.54	0.45	0.22	0.78	0.67
11JY2229	P591c41y2GENF3-1-1-2-B-B-B	440	LP	1	32.05	43.14	2.38	1.58	0.27	0.15	1.35	0.41
11JY2230	PAZM 6053	441	LP	1	26.22	43.31	2.45	1.77	0.28	0.12	1.65	0.40
11JY2231	Pool 21 x Pool 22	442	LP	1	39.72	39.14	2.50	2.06	0.33	0.13	0.99	0.46
11JY2233	R15	443	LP	1	44.58	69.48	4.34	2.92	0.37	0.20	1.56	0.58
11JY2234	RDOM 330	444	LP	1	31.38	43.81	2.23	2.22	0.29	0.14	1.40	0.44
11JY2238	VL0512452	445	LP	1	38.72	58.14	4.08	3.18	0.50	0.29	1.50	0.79
11JY2239	VL0512464	446	LP	1	36.05	52.47	3.34	2.17	0.36	0.18	1.46	0.53

Seed ID	Acc	Gen	trt.	rep	SL	RL	SFW	RFW	SDW	RDW	RSR	TDM
11JY2240	VL05128	447	LP	1	37.50	60.01	2.13	1.95	0.34	0.14	1.60	0.48
11JY2241	VL052	448	LP	1	38.91	41.48	3.18	2.11	0.33	0.16	1.07	0.49
11JY2243	VL05353	449	LP	1	50.91	58.48	5.22	4.02	0.52	0.29	1.15	0.82
11JY2244	VL054178	450	LP	1	40.50	63.68	3.03	2.51	0.40	0.19	1.57	0.60
11JY2246	VL054881	451	LP	1	43.16	48.01	2.90	2.68	0.42	0.25	1.11	0.67
11JY2248	VL0556	452	LP	1	35.16	59.01	2.45	2.10	0.30	0.12	1.68	0.42
11JY2249	VL05561	453	LP	1	38.16	58.68	2.91	2.50	0.37	0.18	1.54	0.55
11JY2250	VL0558	454	LP	1	35.08	64.31	4.37	3.30	0.41	0.28	1.83	0.69
11JY2252	VL05616	455	LP	1	39.41	52.14	4.26	2.76	0.39	0.25	1.32	0.65
11JY2255	VL056942	456	LP	1	33.08	50.14	3.43	2.07	0.36	0.21	1.52	0.57
11JY2259	VL062784	457	LP	1	37.41	46.14	3.43	1.90	0.36	0.21	1.23	0.56
11JY2260	VL062785	458	LP	1	45.08	63.81	4.89	3.01	0.48	0.30	1.42	0.78
11JY2262	VL06384	459	LP	1	36.58	51.31	3.16	1.84	0.26	0.17	1.40	0.43
11JY2263	ZM521B-66-4-1-1-BB-B-B-B	460	LP	1	43.74	53.06	3.77	2.30	0.36	0.18	1.21	0.54
11JY2264	川29♀	461	LP	1	39.41	43.06	3.58	1.87	0.35	0.16	1.09	0.50
11JY2265	慈溪白糯	462	LP	1	34.08	46.73	2.77	2.17	0.26	0.18	1.37	0.43
11JY2266	独紫	463	LP	1	43.74	46.39	4.45	2.43	0.37	0.20	1.06	0.57
11JY2268	交51	464	LP	1	38.41	43.73	4.09	2.49	0.37	0.20	1.14	0.57
11JY2269	双M9	465	LP	1	39.58	47.31	4.74	3.26	0.51	0.25	1.20	0.77
11JY2270	四川地方种质	466	LP	1	52.08	52.73	5.43	3.16	0.52	0.24	1.01	0.76
11JY2271	豫综BC15-2	467	LP	1	41.41	48.06	3.88	1.85	0.43	0.22	1.16	0.66
11JY2279	407	468	LP	1	33.74	31.06	2.71	1.41	0.25	0.12	0.92	0.37
11JY2280	412	469	LP	1	39.08	37.81	4.07	2.33	0.37	0.21	0.97	0.58
11JY2288	485	470	LP	1	46.24	56.81	5.55	4.00	0.51	0.32	1.23	0.83
11JY2290	495	471	LP	1	40.58	53.48	3.71	2.54	0.35	0.23	1.32	0.58
11JY2311	8001	472	LP	1	35.74	38.81	3.01	1.59	0.27	0.17	1.09	0.45
11JY2317	[(CML395/CML444)-B-4-1-3-1-B/CML395//DTPWC8F31-1-1-2-2]-5-1-2-2-BB-B-B-B	473	LP	1	33.24	65.81	2.68	2.22	0.25	0.19	1.98	0.44
11JY2327	4F1	474	LP	1	40.24	42.48	4.95	3.85	0.50	0.33	1.06	0.83
11JY2356	B73	475	LP	1	40.24	40.73	3.78	1.70	0.42	0.19	1.01	0.62
11JY2391	E28	476	LP	1	40.58	45.48	4.85	2.90	0.44	0.20	1.12	0.64
11JY2394	ES40	477	LP	1	37.08	42.81	3.37	1.95	0.29	0.17	1.15	0.46
11JY2396	F42	478	LP	1	41.24	39.81	6.84	4.25	0.67	0.47	0.97	1.13
11JY2398	FR19	479	LP	1	31.58	43.14	2.11	1.37	0.24	0.10	1.37	0.34
11JY2426	MBNA	480	LP	1	38.83	44.68	3.20	2.30	0.38	0.17	1.15	0.55

Seed ID	Acc	Gen	trt.	rep	SL	RL	SFW	RFW	SDW	RDW	RSR	TDM
11JY2434	NS701	481	LP	1	42.83	66.01	3.28	2.44	0.39	0.18	1.54	0.57
11JY2445	PHG83	482	LP	1	44.50	60.34	4.07	3.37	0.43	0.17	1.36	0.61
11JY2446	PHN47	483	LP	1	38.50	43.01	3.30	2.87	0.41	0.17	1.12	0.58
11JY2453	R09	484	LP	1	43.58	60.31	4.44	3.81	0.49	0.33	1.38	0.82
11JY2467	Va35	485	LP	1	34.74	49.48	4.24	2.41	0.36	0.22	1.42	0.58
11JY2470	W8304	486	LP	1	48.08	37.48	5.30	2.39	0.46	0.28	0.78	0.73
11JY2474	XZY364-1	487	LP	1	35.66	38.06	1.04	0.95	0.22	0.12	1.07	0.34
11JY2491	长3154	488	LP	1	34.99	40.89	1.87	1.58	0.34	0.14	1.17	0.48
11JY2498	丹3130	489	LP	1	32.41	38.48	3.24	2.18	0.32	0.23	1.19	0.55
11JY2500	丹340	490	LP	1	35.33	45.73	2.91	2.69	0.37	0.23	1.29	0.59
11JY2504	丹360	491	LP	1	40.08	46.81	4.04	2.24	0.45	0.21	1.17	0.67
11JY2522	辐746	492	LP	1	45.41	58.81	4.41	2.75	0.40	0.27	1.30	0.66
11JY2526	旱21	493	LP	1	40.74	44.14	4.28	2.36	0.39	0.21	1.08	0.61
11JY2547	吉419	494	LP	1	48.08	62.81	5.07	3.41	0.50	0.36	1.31	0.87
11JY2557	吉846	495	LP	1	50.16	60.68	6.05	4.00	0.63	0.28	1.21	0.92
11JY2563	冀研01-3-2-2-1-5-1	496	LP	1	40.16	62.68	4.80	2.41	0.49	0.21	1.56	0.70
11JY2570	金黄96C	497	LP	1	41.99	56.39	2.75	2.14	0.34	0.20	1.34	0.54
11JY2574	辽138	498	LP	1	39.83	57.68	4.67	2.60	0.44	0.18	1.45	0.63
11JY2587	辽孤001	499	LP	1	42.74	54.06	4.52	2.64	0.49	0.27	1.26	0.76
11JY2603	齐205	500	LP	1	50.41	45.73	4.89	2.57	0.51	0.29	0.91	0.80
11JY2606	齐310	501	LP	1	34.74	30.06	2.91	1.41	0.31	0.14	0.87	0.45
11JY2621	双105	502	LP	1	42.41	36.06	3.67	2.00	0.33	0.16	0.85	0.50
11JY2623	双741	503	LP	1	44.41	39.73	4.32	2.10	0.39	0.17	0.89	0.56
11JY2667	郑22	504	LP	1	40.50	51.68	3.83	2.62	0.46	0.23	1.28	0.70
11JY2672	郑29	505	LP	1	44.49	41.39	4.26	2.75	0.47	0.27	0.93	0.73
11JY2675	郑30	506	LP	1	40.83	47.01	3.92	2.25	0.38	0.16	1.15	0.55
11JY2677	郑35	507	LP	1	37.83	54.01	3.84	2.82	0.46	0.26	1.43	0.73
11JY2697	综31	508	LP	1	47.50	46.34	4.73	2.53	0.49	0.23	0.98	0.72
12JY0001	C5 RIL P2	509	LP	1	40.00	48.89	2.32	2.61	0.42	0.21	1.22	0.63
12JY0002	C5 RIL P1	510	LP	1	34.66	45.39	2.40	2.83	0.39	0.25	1.31	0.65
12JY0015	[(SML*SMQPM)*(MTL*SMQPM)]F1S6-1-25-BB-1-B	511	LP	1	46.33	32.73	2.55	1.71	0.34	0.14	0.71	0.48
12JY0017	[CML159/[CML159/[MSRXPOOL9]C1F2-205-1(OSU23i)-5-3-X-X-1-BB]F2-3sx]-8-1-1-BB-1-B	512	LP	1	38.33	60.06	2.80	1.91	0.42	0.23	1.57	0.65

Seed ID	Acc	Gen	trt.	rep	SL	RL	SFW	RFW	SDW	RDW	RSR	TDM
12JY0018	[CML198/LPSC3H144-1-2-2-2-2-#-BB]-1-4-1-1-4-B*4-B-B-B	513	LP	1	40.33	66.06	3.37	2.93	0.41	0.28	1.64	0.69
12JY0029	[DTPWC8F31-4-2-1-6/CML444//ZM521B-66-4-1-1-1-BB]-3-2-1-B-B-B	514	LP	1	27.66	53.39	1.59	1.91	0.21	0.16	1.93	0.38
12JY0040	18-599	515	LP	1	53.33	38.73	4.26	1.89	0.58	0.25	0.73	0.83
12JY0041	18-599(RED)	516	LP	1	39.99	35.39	3.21	1.29	0.43	0.17	0.88	0.60
12JY0108	CML330	517	LP	1	32.99	68.06	1.72	2.42	0.34	0.19	2.06	0.53
12JY0122	CML418	518	LP	1	37.99	40.06	2.01	1.95	0.33	0.20	1.05	0.53
12JY0146	CML504	519	LP	1	34.99	65.06	1.80	1.31	0.27	0.19	1.86	0.46
12JY0156	Cuba/Guad C3 F53-3-1-1-B-B-B	520	LP	1	43.66	54.73	3.86	2.45	0.44	0.28	1.25	0.72
12JY0164	ECA-MOROSR(BC1)F2-7-ECAVEE7/PL15QPMC7SRC1F2//POOL15QPMSR-B-4-B-B	521	LP	1	30.99	36.73	1.58	1.24	0.24	0.12	1.18	0.36
12JY0167	INTA-191-2-1-2-B*8-B-B-B	522	LP	1	38.66	30.39	1.72	1.19	0.29	0.14	0.79	0.43
12JY0173	La Posta Seq C7-F86-1-1-1-1-B-B-B	523	LP	1	24.49	35.39	0.96	1.26	0.20	0.10	1.44	0.30
12JY0185	P501SRc0-F2-47-3-1-1-B-B-B-B	524	LP	1	26.99	43.06	0.21	1.16	0.15	0.11	1.60	0.27
12JY0208	VL05610	525	LP	1	39.87	32.27	2.25	1.44	0.33	0.17	0.81	0.51
12JY0226	178	526	LP	1	28.66	19.06	0.95	1.54	0.25	0.16	0.67	0.41
12JY0228	273	527	LP	1	42.79	47.10	3.79	3.00	0.45	0.25	1.10	0.69
12JY0229	288	528	LP	1	38.33	35.73	1.98	1.59	0.30	0.17	0.93	0.47
12JY0240	764	529	LP	1	36.79	49.43	2.44	1.60	0.30	0.18	1.34	0.48
12JY0246	6103	530	LP	1	36.33	33.39	3.17	2.26	0.28	0.21	0.92	0.49
12JY0252	81565	531	LP	1	39.54	36.93	2.88	2.01	0.34	0.21	0.93	0.55
12JY0264	634-11511	532	LP	1	43.87	45.10	4.13	1.99	0.46	0.22	1.03	0.68
12JY0265	698-1	533	LP	1	33.99	38.73	2.26	1.82	0.27	0.16	1.14	0.44
12JY0308	FAPW	534	LP	1	44.74	41.81	4.72	2.63	0.46	0.28	0.93	0.73
12JY0322	LH132	535	LP	1	42.74	65.14	3.58	2.70	0.28	0.18	1.52	0.46
12JY0323	LH51	536	LP	1	36.08	34.14	3.14	2.28	0.24	0.17	0.95	0.41
12JY0325	LX9801	537	LP	1	44.08	60.48	4.60	3.48	0.35	0.22	1.37	0.57
12JY0329	Mo17	538	LP	1	37.74	27.81	2.84	2.42	0.24	0.12	0.74	0.36
12JY0347	R08	539	LP	1	39.41	50.48	2.15	1.85	0.23	0.10	1.28	0.32
12JY0349	RP125	540	LP	1	41.66	57.98	4.41	2.94	0.41	0.26	1.39	0.67
12JY0365	Zhao835	541	LP	1	42.74	42.81	5.72	3.37	0.44	0.26	1.00	0.70
12JY0370	昌7-2	542	LP	1	32.37	42.60	1.96	1.12	0.25	0.11	1.32	0.37
12JY0383	丹598	543	LP	1	44.20	42.77	3.66	2.45	0.43	0.20	0.97	0.64
12JY0401	黄早四	544	LP	1	31.16	38.73	2.02	1.46	0.30	0.16	1.24	0.46

Seed ID	Acc	Gen	trt.	rep	SL	RL	SFW	RFW	SDW	RDW	RSR	TDM
12JY0416	吉853	545	LP	1	34.41	48.48	2.54	1.95	0.18	0.15	1.41	0.33
12JY0443	南21-3	546	LP	1	46.87	58.10	4.33	2.93	0.44	0.25	1.24	0.68
12JY0450	齐319	547	LP	1	46.54	51.43	4.54	3.60	0.46	0.30	1.11	0.76
12JY0457	沈5003	548	LP	1	27.66	55.06	0.79	1.49	0.23	0.18	1.99	0.41
12JY0462	四287	549	LP	1	42.70	64.77	4.35	4.03	0.40	0.30	1.52	0.70
12JY0473	铁7922	550	LP	1	36.41	46.14	4.39	2.27	0.28	0.19	1.27	0.47
11JY1255	C5 RIL 2	1	LP	2	34.86	59.64	3.52	4.05	0.45	0.28	1.71	0.73
11JY1256	C5 RIL 3	2	LP	2	34.52	51.64	2.21	1.39	0.35	0.13	1.50	0.49
11JY1258	C5 RIL 5	3	LP	2	34.19	46.98	2.94	2.19	0.36	0.15	1.37	0.51
11JY1259	C5 RIL 6	4	LP	2	40.52	47.98	3.65	2.48	0.49	0.20	1.18	0.69
11JY1262	C5 RIL 10	5	LP	2	32.69	43.14	2.31	2.08	0.30	0.14	1.32	0.44
11JY1263	C5 RIL 11	6	LP	2	41.19	58.64	3.32	3.47	0.44	0.24	1.42	0.68
11JY1264	C5 RIL 12	7	LP	2	35.86	64.31	2.54	2.70	0.33	0.19	1.79	0.51
11JY1267	C5 RIL 17	8	LP	2	32.52	55.98	2.97	2.85	0.43	0.23	1.72	0.65
11JY1268	C5 RIL 18	9	LP	2	37.86	51.31	3.86	3.18	0.49	0.22	1.36	0.71
11JY1269	C5 RIL 19	10	LP	2	32.52	43.98	4.01	4.17	0.52	0.30	1.35	0.82
11JY1271	C5 RIL 21	11	LP	2	36.86	51.98	3.02	2.87	0.46	0.21	1.41	0.67
11JY1272	C5 RIL 24	12	LP	2	29.52	43.31	2.56	2.43	0.36	0.19	1.47	0.55
11JY1274	C5 RIL 26	13	LP	2	32.52	54.64	3.97	3.52	0.51	0.26	1.68	0.77
11JY1275	C5 RIL 27	14	LP	2	32.19	43.14	1.95	2.35	0.36	0.14	1.34	0.50
11JY1276	C5 RIL 28	15	LP	2	32.19	34.31	2.35	1.31	0.36	0.13	1.07	0.49
11JY1278	C5 RIL 30	16	LP	2	29.86	46.98	2.60	2.52	0.38	0.21	1.57	0.59
11JY1279	C5 RIL 31	17	LP	2	30.86	53.98	3.37	2.44	0.48	0.21	1.75	0.69
11JY1280	C5 RIL 32	18	LP	2	37.52	48.98	3.67	3.00	0.53	0.27	1.31	0.80
11JY1281	C5 RIL 34	19	LP	2	29.52	39.31	2.34	2.48	0.38	0.19	1.33	0.57
11JY1282	C5 RIL 36	20	LP	2	40.91	44.48	5.09	3.97	0.56	0.29	1.09	0.85
11JY1283	C5 RIL 38	21	LP	2	32.24	49.81	3.03	3.22	0.34	0.21	1.54	0.55
11JY1284	C5 RIL 39	22	LP	2	35.91	42.48	2.98	2.06	0.43	0.16	1.18	0.60
11JY1285	C5 RIL 40	23	LP	2	37.24	39.14	3.56	3.31	0.46	0.24	1.05	0.70
11JY1286	C5 RIL 41	24	LP	2	33.91	29.81	2.33	1.89	0.32	0.14	0.88	0.46
11JY1287	C5 RIL 42	25	LP	2	33.91	48.48	3.01	2.17	0.39	0.19	1.43	0.58
11JY1288	C5 RIL 44	26	LP	2	38.58	34.48	3.50	3.68	0.48	0.23	0.89	0.71
11JY1289	C5 RIL 45	27	LP	2	37.24	42.48	2.85	2.09	0.40	0.19	1.14	0.60
11JY1290	C5 RIL 46	28	LP	2	34.58	40.98	2.81	2.79	0.40	0.22	1.19	0.62
11JY1292	C5 RIL 48	29	LP	2	38.08	41.48	4.03	2.86	0.46	0.21	1.09	0.67
11JY1293	C5 RIL 49	30	LP	2	39.58	44.81	3.21	2.96	0.50	0.27	1.13	0.77

Seed ID	Acc	Gen	trt.	rep	SL	RL	SFW	RFW	SDW	RDW	RSR	TDM
11JY1299	C5 RIL 57	31	LP	2	37.24	53.81	3.40	4.36	0.46	0.30	1.44	0.76
11JY1301	C5 RIL 59	32	LP	2	33.58	44.98	2.58	3.52	0.36	0.21	1.34	0.57
11JY1305	C5 RIL 64	33	LP	2	38.24	51.48	2.84	3.12	0.47	0.23	1.35	0.70
11JY1310	C5 RIL 68	34	LP	2	42.91	49.48	4.65	4.84	0.54	0.34	1.15	0.88
11JY1312	C5 RIL 70	35	LP	2	30.77	54.44	2.34	2.22	0.27	0.18	1.77	0.44
11JY1315	C5 RIL 82	36	LP	2	36.77	40.28	2.96	1.42	0.37	0.15	1.10	0.52
11JY1316	C5 RIL 74	37	LP	2	26.10	40.11	1.49	1.77	0.24	0.16	1.54	0.40
11JY1318	C5 RIL 78	38	LP	2	29.77	44.44	1.58	1.60	0.24	0.15	1.49	0.38
11JY1319	C5 RIL 79	39	LP	2	42.44	46.44	3.85	3.20	0.51	0.25	1.09	0.76
11JY1320	C5 RIL 80	40	LP	2	31.27	41.28	1.24	1.73	0.20	0.16	1.32	0.35
11JY1323	C5 RIL 84	41	LP	2	33.77	51.78	2.83	2.22	0.37	0.22	1.53	0.59
11JY1324	C5 RIL 86	42	LP	2	37.44	45.78	3.87	3.00	0.39	0.24	1.22	0.63
11JY1326	C5 RIL 88	43	LP	2	37.10	52.78	3.59	2.81	0.46	0.23	1.42	0.69
11JY1327	C5 RIL 89	44	LP	2	31.10	40.44	0.74	0.66	0.20	0.13	1.30	0.33
11JY1328	C5 RIL 90	45	LP	2	30.77	39.28	1.91	1.48	0.24	0.13	1.28	0.37
11JY1329	C5 RIL 92	46	LP	2	26.27	40.28	1.69	0.94	0.27	0.14	1.53	0.41
11JY1333	C5 RIL 96	47	LP	2	46.44	53.44	5.65	4.38	0.54	0.31	1.15	0.85
11JY1334	C5 RIL 99	48	LP	2	35.44	45.78	3.79	2.77	0.42	0.23	1.29	0.65
11JY1336	C5 RIL 102	49	LP	2	33.10	47.11	2.26	2.05	0.38	0.34	1.42	0.72
11JY1337	C5 RIL 103	50	LP	2	31.10	38.11	3.00	2.06	0.39	0.18	1.23	0.58
11JY1339	C5 RIL 106	51	LP	2	37.44	40.44	3.80	2.85	0.43	0.23	1.08	0.66
11JY1341	C5 RIL 108	52	LP	2	32.24	56.73	2.65	3.31	0.32	0.25	1.76	0.57
11JY1349	C5 RIL 119	53	LP	2	33.24	39.73	3.23	2.42	0.37	0.22	1.19	0.59
11JY1350	C5 RIL 120	54	LP	2	34.24	44.06	2.90	2.53	0.31	0.18	1.29	0.49
11JY1351	C5 RIL 121	55	LP	2	29.24	51.73	2.17	3.18	0.24	0.23	1.77	0.47
11JY1352	C5 RIL 122	56	LP	2	39.91	65.73	5.57	4.24	0.39	0.20	1.65	0.59
11JY1354	C5 RIL 124	57	LP	2	39.24	47.39	3.64	2.26	0.44	0.20	1.21	0.63
11JY1356	C5 RIL 126	58	LP	2	34.24	54.73	3.06	2.21	0.36	0.19	1.60	0.55
11JY1357	C5 RIL 128	59	LP	2	33.24	50.06	2.95	1.84	0.36	0.18	1.51	0.54
11JY1360	C5 RIL 131	60	LP	2	35.58	53.73	3.62	2.21	0.44	0.24	1.51	0.68
11JY1362	C5 RIL 133	61	LP	2	32.24	48.73	2.99	1.57	0.32	0.17	1.51	0.49
11JY1363	C5 RIL 134	62	LP	2	44.58	69.06	6.48	4.45	0.72	0.32	1.55	1.04
11JY1364	C5 RIL 135	63	LP	2	33.58	45.73	2.87	2.17	0.33	0.18	1.36	0.51
11JY1365	C5 RIL 137	64	LP	2	34.24	55.23	2.76	2.31	0.33	0.16	1.61	0.50
11JY1366	C5 RIL 138	65	LP	2	33.24	56.06	3.17	2.65	0.39	0.21	1.69	0.60
11JY1367	C5 RIL 139	66	LP	2	36.24	52.73	3.52	2.32	0.41	0.21	1.45	0.63

Seed ID	Acc	Gen	trt.	rep	SL	RL	SFW	RFW	SDW	RDW	RSR	TDM
11JY1368	C5 RIL 140	67	LP	2	35.91	43.39	3.15	2.14	0.38	0.20	1.21	0.58
11JY1369	C5 RIL 141	68	LP	2	37.58	54.73	4.44	2.75	0.49	0.19	1.46	0.68
11JY1370	C5 RIL 142	69	LP	2	32.58	45.39	3.89	1.71	0.39	0.15	1.39	0.54
11JY1372	C5 RIL 144	70	LP	2	38.24	59.06	4.51	3.51	0.57	0.28	1.54	0.85
11JY1374	C5 RIL 146	71	LP	2	32.41	69.14	3.53	4.24	0.35	0.29	2.13	0.64
11JY1376	C5 RIL 150	72	LP	2	38.08	66.14	4.64	4.66	0.41	0.29	1.74	0.70
11JY1377	C5 RIL 151	73	LP	2	37.53	52.95	1.73	1.20	0.18	0.14	1.41	0.32
11JY1378	C5 RIL 152	74	LP	2	32.50	47.30	1.71	1.33	0.20	0.16	1.46	0.36
11JY1379	C5 RIL 153	75	LP	2	35.60	40.20	2.31	1.58	0.24	0.14	1.13	0.38
11JY1382	C5 RIL 156	76	LP	2	32.50	40.60	2.11	0.93	0.23	0.14	1.25	0.37
11JY1383	C5 RIL 157	77	LP	2	38.27	42.73	2.43	1.28	0.24	0.14	1.12	0.38
11JY1384	C5 RIL 158	78	LP	2	36.50	63.65	2.58	1.55	0.32	0.19	1.74	0.51
11JY1385	C5 RIL 159	79	LP	2	25.20	34.50	1.10	0.64	0.10	0.08	1.37	0.18
11JY1387	C5 RIL 161	80	LP	2	35.70	45.23	2.32	1.47	0.22	0.16	1.27	0.38
11JY1388	C5 RIL 162	81	LP	2	32.50	41.00	1.17	0.87	0.13	0.10	1.26	0.23
11JY1389	C5 RIL 163	82	LP	2	46.45	55.75	4.20	3.16	0.38	0.31	1.20	0.69
11JY1391	C5 RIL 165	83	LP	2	40.00	41.20	2.53	1.40	0.29	0.20	1.03	0.49
11JY1394	C5 RIL 169	84	LP	2	31.80	40.00	1.26	0.60	0.15	0.12	1.26	0.27
11JY1396	C5 RIL 171	85	LP	2	36.70	42.30	2.91	1.64	0.28	0.21	1.15	0.49
11JY1397	C5 RIL 172	86	LP	2	35.87	49.40	1.88	1.59	0.21	0.16	1.38	0.37
11JY1399	C5 RIL 174	87	LP	2	40.00	39.60	3.76	2.30	0.38	0.36	0.99	0.74
11JY1400	C5 RIL 176	88	LP	2	37.87	35.20	3.38	2.60	0.33	0.24	0.93	0.57
11JY1401	C5 RIL 177	89	LP	2	38.55	61.15	3.40	3.63	0.33	0.30	1.59	0.63
11JY1402	C5 RIL 178	90	LP	2	34.27	50.00	2.97	2.69	0.30	0.23	1.46	0.53
11JY1404	C5 RIL 182	91	LP	2	35.33	48.10	3.36	2.26	0.35	0.27	1.36	0.62
11JY1405	C5 RIL 183	92	LP	2	35.33	48.10	3.36	2.26	0.35	0.27	1.36	0.62
11JY1406	C5 RIL 184	93	LP	2	32.30	51.50	2.01	1.92	0.23	0.14	1.59	0.37
11JY1409	C5 RIL 187	94	LP	2	29.00	37.70	1.75	1.93	0.22	0.20	1.30	0.42
11JY1410	C5 RIL 188	95	LP	2	28.80	46.00	2.02	1.72	0.22	0.20	1.60	0.42
11JY1412	C5 RIL 191	96	LP	2	38.47	71.00	3.38	2.95	0.38	0.32	1.85	0.70
11JY1413	C5 RIL 192	97	LP	2	38.65	38.25	1.63	1.10	0.19	0.21	0.99	0.40
11JY1414	C5 RIL 193	98	LP	2	29.50	44.43	1.48	1.40	0.20	0.20	1.51	0.40
11JY1416	C5 RIL 195	99	LP	2	28.00	32.30	2.00	1.08	0.27	0.23	1.15	0.50
11JY1418	C5 RIL 198	100	LP	2	31.30	55.70	2.55	2.06	0.32	0.28	1.78	0.60
11JY1420	C5 RIL 200	101	LP	2	31.10	28.47	2.43	1.36	0.30	0.22	0.92	0.52
11JY1421	C5 RIL 203	102	LP	2	28.60	42.30	1.61	0.98	0.18	0.15	1.48	0.33

Seed ID	Acc	Gen	trt.	rep	SL	RL	SFW	RFW	SDW	RDW	RSR	TDM
11JY1422	C5 RIL 204	103	LP	2	30.20	38.80	1.41	0.63	0.66	0.19	1.28	0.85
11JY1423	C5 RIL 205	104	LP	2	40.10	61.10	1.53	2.35	0.32	0.25	1.52	0.57
11JY1424	C5 RIL 206	105	LP	2	34.70	69.50	2.21	1.98	0.25	0.22	2.00	0.47
11JY1425	C5 RIL 207	106	LP	2	43.60	47.40	3.64	2.26	0.42	0.40	1.09	0.82
11JY1426	C5 RIL 208	107	LP	2	43.60	47.40	3.64	2.26	0.42	0.40	1.09	0.82
11JY1428	C5 RIL 210	108	LP	2	30.35	49.50	2.46	2.26	0.26	0.28	1.63	0.54
11JY1429	C5 RIL 211	109	LP	2	29.33	34.73	1.67	1.88	0.33	0.23	1.18	0.56
11JY1429	C5 RIL 211	110	LP	2	36.60	45.70	2.10	1.89	0.26	0.18	1.25	0.44
11JY1431	C5 RIL 214	111	LP	2	31.60	47.00	2.21	2.27	0.24	0.22	1.49	0.46
11JY1433	C5 RIL 216	112	LP	2	24.30	35.60	1.16	0.53	0.15	0.12	1.47	0.27
11JY1434	C5 RIL 217	113	LP	2	38.30	37.50	4.41	1.95	0.48	0.42	0.98	0.90
11JY1435	C5 RIL 218	114	LP	2	37.20	63.50	3.24	2.63	0.39	0.21	1.71	0.60
11JY1437	C5 RIL 220	115	LP	2	32.73	45.97	2.04	1.82	0.20	0.19	1.40	0.39
11JY1440	C5 RIL 225	116	LP	2	29.70	48.95	2.20	2.00	0.22	0.16	1.65	0.38
11JY1442	C5 RIL 227	117	LP	2	31.00	42.43	2.14	2.08	0.23	0.16	1.37	0.39
11JY1444	C5 RIL 229	118	LP	2	36.90	60.60	2.92	2.43	0.26	0.22	1.64	0.48
11JY1454	C5 RIL 240	119	LP	2	35.50	52.40	3.33	3.01	0.38	0.31	1.48	0.69
11JY1455	C5 RIL 241	120	LP	2	28.40	36.37	1.32	1.32	0.30	0.12	1.28	0.42
11JY1456	C5 RIL 243	121	LP	2	29.00	42.45	1.66	0.94	0.23	0.17	1.46	0.40
11JY1459	C5 RIL P2	122	LP	2	33.80	52.40	3.19	1.45	0.30	0.24	1.55	0.54
11JY1460	C5 RIL P1	123	LP	2	31.80	37.50	1.37	0.60	0.15	0.11	1.18	0.26
11JY1461	C6 RIL 3	124	LP	2	43.67	25.30	2.76	1.61	0.21	0.14	0.58	0.35
11JY1462	C6 RIL 14	125	LP	2	52.17	58.53	4.64	3.08	0.39	0.21	1.12	0.60
11JY1463	C6 RIL 23	126	LP	2	34.67	32.67	1.67	1.50	0.19	0.13	0.94	0.32
11JY1464	C6 RIL 24	127	LP	2	35.33	32.67	2.03	1.48	0.29	0.19	0.92	0.48
11JY1465	C6 RIL 25	128	LP	2	40.33	50.00	3.10	2.95	0.36	0.27	1.24	0.63
11JY1466	C6 RIL 29	129	LP	2	32.67	37.67	1.84	2.12	0.25	0.20	1.15	0.45
11JY1468	C6 RIL 144	130	LP	2	33.00	46.00	1.45	1.32	0.22	0.13	1.39	0.35
11JY1469	C6 RIL 203	131	LP	2	37.33	48.00	2.39	2.49	0.27	0.19	1.29	0.46
11JY1470	C6 RIL 212	132	LP	2	34.00	30.00	1.71	1.25	0.20	0.13	0.88	0.33
11JY1472	C6 RIL 219	133	LP	2	35.00	39.00	1.80	1.74	0.22	0.15	1.11	0.37
11JY1473	C6 RIL 239	134	LP	2	41.40	51.40	3.74	2.82	0.45	0.31	1.24	0.76
11JY1474	C6 RIL 243	135	LP	2	38.53	39.53	2.99	2.21	0.36	0.22	1.03	0.58
11JY1475	C6 RIL 272	136	LP	2	54.83	66.83	7.52	3.83	0.54	0.22	1.22	0.76
11JY1476	C6 RIL 273	137	LP	2	37.93	39.43	2.83	1.72	0.38	0.28	1.04	0.66
11JY1477	C6 RIL 283	138	LP	2	39.47	45.10	2.18	1.21	0.31	0.20	1.14	0.51

Seed ID	Acc	Gen	trt.	rep	SL	RL	SFW	RFW	SDW	RDW	RSR	TDM
11JY1478	C6 RIL 293	139	LP	2	37.73	39.50	2.23	1.14	0.34	0.25	1.05	0.59
11JY1479	C6 RIL 298	140	LP	2	35.70	29.80	2.96	0.90	0.42	0.22	0.83	0.64
11JY1480	C6 RIL 303	141	LP	2	35.13	23.13	2.47	0.74	0.34	0.19	0.66	0.53
11JY1481	C6 RIL 327	142	LP	2	48.60	35.30	3.68	2.78	0.41	0.33	0.73	0.74
11JY1482	C6 RIL 341	143	LP	2	31.60	24.80	2.16	1.67	0.26	0.17	0.78	0.43
11JY1483	C6 RIL 348	144	LP	2	17.90	44.50	1.15	0.54	0.16	0.10	2.49	0.26
11JY1484	C6 RIL 402	145	LP	2	33.80	31.45	2.42	1.32	0.28	0.19	0.93	0.47
11JY1485	C6 RIL 403	146	LP	2	34.45	43.10	2.29	1.76	0.29	0.23	1.25	0.52
11JY1487	C6 RIL 5	147	LP	2	48.40	30.63	3.99	2.07	0.35	0.14	0.63	0.49
11JY1488	C6 RIL 7	148	LP	2	40.00	51.80	5.00	3.18	0.51	0.42	1.30	0.93
11JY1489	C6 RIL 10	149	LP	2	37.90	37.70	3.18	1.71	0.36	0.23	0.99	0.59
11JY1490	C6 RIL 15	150	LP	2	44.57	48.43	3.71	2.79	0.41	0.28	1.09	0.69
11JY1491	C6 RIL 19	151	LP	2	40.40	56.70	3.03	2.98	0.34	0.26	1.40	0.60
11JY1493	C6 RIL 21	152	LP	2	37.73	34.07	3.22	2.47	0.34	0.22	0.90	0.56
11JY1494	C6 RIL 23	153	LP	2	37.87	50.80	2.70	2.58	0.30	0.22	1.34	0.52
11JY1495	C6 RIL 24	154	LP	2	38.33	39.13	3.55	3.17	0.39	0.29	1.02	0.68
11JY1496	C6 RIL 25	155	LP	2	42.37	47.00	2.69	3.15	0.33	0.27	1.11	0.60
11JY1497	C6 RIL 28	156	LP	2	26.00	28.50	1.00	0.59	0.11	0.08	1.10	0.19
11JY1498	C6 RIL 29	157	LP	2	45.20	53.67	3.84	4.20	0.39	0.35	1.19	0.74
11JY1499	C6 RIL 30	158	LP	2	53.70	36.63	6.22	3.57	0.68	0.30	0.68	0.98
11JY1500	C6 RIL 31	159	LP	2	39.45	56.70	4.56	2.67	0.56	0.35	1.44	0.91
11JY1501	C6 RIL 32	160	LP	2	35.67	37.80	1.64	0.98	0.21	0.13	1.06	0.34
11JY1502	C6 RIL 33	161	LP	2	36.80	34.50	2.48	1.43	0.31	0.20	0.94	0.51
11JY1503	C6 RIL 36	162	LP	2	37.10	29.67	1.96	1.90	0.36	0.33	0.80	0.69
11JY1504	C6 RIL 39	163	LP	2	31.70	39.10	1.93	1.88	0.25	0.20	1.23	0.45
11JY1505	C6 RIL 40	164	LP	2	32.83	31.37	1.96	1.14	0.23	0.15	0.96	0.38
11JY1506	C6 RIL 42	165	LP	2	33.47	33.77	2.65	2.16	0.39	0.25	1.01	0.64
11JY1508	C6 RIL 47	166	LP	2	41.00	46.63	2.86	1.95	0.32	0.22	1.14	0.54
11JY1509	C6 RIL 49	167	LP	2	29.80	32.50	1.75	1.00	0.21	0.13	1.09	0.34
11JY1510	C6 RIL 50	168	LP	2	34.90	53.57	2.86	1.81	0.33	0.27	1.53	0.60
11JY1511	C6 RIL 52	169	LP	2	36.47	22.63	2.12	1.87	0.25	0.14	0.62	0.39
11JY1512	C6 RIL 53	170	LP	2	29.65	39.00	1.27	0.69	0.16	0.10	1.32	0.26
11JY1513	C6 RIL 57	171	LP	2	37.00	39.20	2.84	2.12	0.35	0.25	1.06	0.60
11JY1514	C6 RIL 58	172	LP	2	45.50	26.20	4.68	3.36	0.55	0.36	0.58	0.91
11JY1515	C6 RIL 59	173	LP	2	31.00	33.55	1.42	0.77	0.20	0.13	1.08	0.33
11JY1516	C6 RIL 60	174	LP	2	30.20	36.30	1.33	1.07	0.19	0.12	1.20	0.31

Seed ID	Acc	Gen	trt.	rep	SL	RL	SFW	RFW	SDW	RDW	RSR	TDM
11JY1517	C6 RIL 64	175	LP	2	36.80	35.90	1.97	1.12	0.23	0.15	0.98	0.38
11JY1518	C6 RIL 65	176	LP	2	35.53	28.67	1.74	1.11	0.24	0.18	0.81	0.42
11JY1519	C6 RIL 66	177	LP	2	31.10	39.40	1.29	0.85	0.20	0.14	1.27	0.34
11JY1520	C6 RIL 68	178	LP	2	37.35	29.65	2.14	0.83	0.29	0.16	0.79	0.45
11JY1521	C6 RIL 69	179	LP	2	41.15	38.55	2.40	1.51	0.29	0.22	0.94	0.51
11JY1522	C6 RIL 70	180	LP	2	26.66	40.33	3.97	2.82	0.31	0.17	1.51	0.48
11JY1523	C6 RIL 72	181	LP	2	37.45	48.10	2.40	1.74	0.29	0.24	1.28	0.53
11JY1524	C6 RIL 74	182	LP	2	35.95	40.85	1.70	1.32	0.23	0.18	1.14	0.41
11JY1525	C6 RIL 76	183	LP	2	29.43	36.40	1.44	0.84	0.23	0.16	1.24	0.39
11JY1527	C6 RIL 79	184	LP	2	30.53	30.83	1.44	0.93	0.21	0.15	1.01	0.36
11JY1528	C6 RIL 83	185	LP	2	43.27	31.06	3.03	2.22	0.33	0.21	0.72	0.54
11JY1529	C6 RIL 85	186	LP	2	38.55	44.70	2.47	1.41	0.34	0.23	1.16	0.57
11JY1530	C6 RIL 86	187	LP	2	30.47	29.23	1.15	0.82	0.18	0.12	0.96	0.30
11JY1531	C6 RIL 91	188	LP	2	44.83	33.50	3.25	1.83	0.44	0.29	0.75	0.73
11JY1532	C6 RIL 94	189	LP	2	44.17	36.67	3.25	0.80	0.36	0.15	0.83	0.51
11JY1533	C6 RIL 95	190	LP	2	21.09	45.95	1.50	0.83	0.23	0.17	2.18	0.40
11JY1534	C6 RIL 97	191	LP	2	44.37	58.30	4.75	4.28	0.38	0.30	1.31	0.68
11JY1535	C6 RIL 103	192	LP	2	42.80	49.90	1.95	0.78	0.28	0.19	1.17	0.47
11JY1536	C6 RIL 104	193	LP	2	34.35	36.80	1.63	0.59	0.20	0.14	1.07	0.34
11JY1537	C6 RIL 107	194	LP	2	39.00	35.20	2.06	0.74	0.27	0.18	0.90	0.45
11JY1538	C6 RIL 108	195	LP	2	46.87	33.93	3.25	0.86	0.46	0.24	0.72	0.70
11JY1539	C6 RIL 109	196	LP	2	37.10	34.67	1.92	0.79	0.24	0.14	0.93	0.38
11JY1540	C6 RIL 112	197	LP	2	34.77	33.03	1.40	0.52	0.16	0.12	0.95	0.28
11JY1541	C6 RIL 113	198	LP	2	47.37	37.27	4.15	1.63	0.48	0.30	0.79	0.78
11JY1542	C6 RIL 115	199	LP	2	46.93	26.33	3.09	0.76	0.41	0.16	0.56	0.57
11JY1543	C6 RIL 116	200	LP	2	45.20	47.30	2.42	0.67	0.24	0.11	1.05	0.35
11JY1544	C6 RIL 117	201	LP	2	43.77	49.47	2.95	1.07	0.30	0.21	1.13	0.51
11JY1545	C6 RIL 120	202	LP	2	57.00	56.50	4.87	2.30	0.41	0.21	0.99	0.62
11JY1546	C6 RIL 121	203	LP	2	39.47	34.43	2.01	0.62	0.29	0.16	0.87	0.45
11JY1547	C6 RIL 123	204	LP	2	43.40	37.83	3.27	1.20	0.36	0.25	0.87	0.61
11JY1548	C6 RIL 124	205	LP	2	36.67	30.80	1.38	0.45	0.22	0.14	0.84	0.36
11JY1549	C6 RIL 127	206	LP	2	39.13	37.23	2.03	0.69	0.27	0.14	0.95	0.41
11JY1550	C6 RIL 131	207	LP	2	26.60	48.50	0.91	0.82	0.13	0.12	1.82	0.25
11JY1551	C6 RIL 134	208	LP	2	48.20	44.90	3.74	2.26	0.46	0.27	0.93	0.73
11JY1552	C6 RIL 135	209	LP	2	36.73	54.83	3.01	1.78	0.35	0.21	1.49	0.56
11JY1553	C6 RIL 138	210	LP	2	46.37	42.90	3.03	1.15	0.41	0.21	0.93	0.62

Seed ID	Acc	Gen	trt.	rep	SL	RL	SFW	RFW	SDW	RDW	RSR	TDM
11JY1554	C6 RIL 139	211	LP	2	35.40	39.80	1.52	0.81	0.18	0.12	1.12	0.30
11JY1555	C6 RIL 140	212	LP	2	36.65	33.85	1.63	0.55	0.19	0.11	0.92	0.30
11JY1556	C6 RIL 141	213	LP	2	40.87	50.43	3.03	2.43	0.36	0.18	1.23	0.54
11JY1557	C6 RIL 142	214	LP	2	41.73	46.53	3.08	1.49	0.38	0.22	1.12	0.60
11JY1558	C6 RIL 143	215	LP	2	50.85	44.10	4.35	2.77	0.43	0.23	0.87	0.66
11JY1560	C6 RIL 146	216	LP	2	45.17	40.37	3.14	1.99	0.31	0.18	0.89	0.49
11JY1561	C6 RIL 147	217	LP	2	21.20	25.50	0.71	0.50	0.07	0.06	1.20	0.13
11JY1562	C6 RIL 148	218	LP	2	36.55	40.05	2.10	1.44	0.23	0.14	1.10	0.37
11JY1563	C6 RIL 150	219	LP	2	21.70	30.20	1.20	0.90	0.10	0.07	1.39	0.17
11JY1565	C6 RIL 153	220	LP	2	32.50	43.55	2.31	2.46	0.26	0.19	1.34	0.45
11JY1566	C6 RIL 156	221	LP	2	33.30	54.40	1.77	1.68	0.26	0.18	1.63	0.44
11JY1567	C6 RIL 160	222	LP	2	42.03	33.63	2.99	1.62	0.31	0.18	0.80	0.49
11JY1568	C6 RIL 161	223	LP	2	35.45	39.60	2.08	1.64	0.23	0.16	1.12	0.39
11JY1569	C6 RIL 162	224	LP	2	49.80	37.67	5.84	4.16	0.51	0.32	0.76	0.83
11JY1570	C6 RIL 163	225	LP	2	38.77	45.40	2.56	1.30	0.29	0.19	1.17	0.48
11JY1571	C6 RIL 164	226	LP	2	37.65	33.00	2.36	0.87	0.54	0.16	0.88	0.70
11JY1572	C6 RIL 169	227	LP	2	31.05	28.95	1.15	0.61	0.15	0.09	0.93	0.24
11JY1573	C6 RIL 171	228	LP	2	44.70	23.80	3.00	1.12	0.37	0.21	0.53	0.58
11JY1574	C6 RIL 172	229	LP	2	37.90	47.25	2.22	1.51	0.29	0.24	1.25	0.53
11JY1576	C6 RIL 174	230	LP	2	50.80	37.50	2.91	1.36	0.33	0.18	0.74	0.51
11JY1577	C6 RIL 175	231	LP	2	41.40	42.30	2.40	0.79	0.31	0.19	1.02	0.50
11JY1578	C6 RIL 176	232	LP	2	50.20	31.20	3.70	1.03	0.48	0.25	0.62	0.73
11JY1579	C6 RIL 179	233	LP	2	33.80	30.30	1.68	0.71	0.18	0.12	0.90	0.30
11JY1580	C6 RIL 181	234	LP	2	38.80	29.15	2.57	0.84	0.30	0.14	0.75	0.44
11JY1581	C6 RIL 184	235	LP	2	33.40	27.30	2.70	1.41	0.08	0.04	0.82	0.12
11JY1582	C6 RIL 186	236	LP	2	44.47	40.20	4.44	4.07	0.47	0.31	0.90	0.78
11JY1583	C6 RIL 187	237	LP	2	38.25	35.30	2.28	0.62	0.30	0.16	0.92	0.46
11JY1584	C6 RIL 188	238	LP	2	32.93	39.50	1.66	0.59	0.20	0.13	1.20	0.33
11JY1585	C6 RIL 189	239	LP	2	21.10	25.50	0.82	0.49	0.13	0.10	1.21	0.23
11JY1586	C6 RIL 190	240	LP	2	40.90	36.97	2.23	0.78	0.28	0.15	0.90	0.43
11JY1587	C6 RIL 192	241	LP	2	38.00	36.30	2.20	0.45	0.25	0.20	0.96	0.45
11JY1588	C6 RIL 193	242	LP	2	42.30	50.25	2.29	0.78	0.28	0.21	1.19	0.49
11JY1589	C6 RIL 203	243	LP	2	44.67	50.10	2.74	0.58	0.29	0.18	1.12	0.47
11JY1590	C6 RIL 204	244	LP	2	43.00	39.45	2.47	0.94	0.30	0.25	0.92	0.55
11JY1591	C6 RIL 205	245	LP	2	35.57	37.63	1.28	0.36	0.15	0.13	1.06	0.28
11JY1592	C6 RIL 206	246	LP	2	37.45	44.15	1.46	0.71	0.18	0.10	1.18	0.28

Seed ID	Acc	Gen	trt.	rep	SL	RL	SFW	RFW	SDW	RDW	RSR	TDM
11JY1593	C6 RIL 207	247	LP	2	34.70	46.17	2.73	2.16	0.30	0.16	1.33	0.46
11JY1594	C6 RIL 211	248	LP	2	36.43	34.30	1.74	0.82	0.18	0.12	0.94	0.30
11JY1595	C6 RIL 212	249	LP	2	41.50	47.50	2.44	1.19	0.22	0.15	1.14	0.37
11JY1596	C6 RIL 217	250	LP	2	36.00	24.20	1.52	0.50	0.18	0.11	0.67	0.29
11JY1597	C6 RIL 218	251	LP	2	45.73	42.27	3.14	1.36	0.30	0.16	0.92	0.46
11JY1598	C6 RIL 219	252	LP	2	45.65	45.45	3.02	1.37	0.34	0.18	1.00	0.52
11JY1599	C6 RIL 220	253	LP	2	36.40	49.70	1.95	0.50	0.23	0.17	1.37	0.40
11JY1600	C6 RIL 225	254	LP	2	45.47	33.57	4.61	2.58	0.42	0.23	0.74	0.65
11JY1601	C6 RIL 230	255	LP	2	38.77	60.07	2.78	2.74	0.36	0.25	1.55	0.61
11JY1602	C6 RIL 231	256	LP	2	45.97	26.87	3.22	2.49	0.41	0.24	0.58	0.65
11JY1603	C6 RIL 232	257	LP	2	40.90	44.75	2.94	2.81	0.36	0.24	1.09	0.60
11JY1604	C6 RIL 234	258	LP	2	55.00	62.27	6.06	5.68	0.59	0.35	1.13	0.94
11JY1605	C6 RIL 235	259	LP	2	51.73	55.60	4.49	4.59	0.49	0.32	1.07	0.81
11JY1606	C6 RIL 236	260	LP	2	38.60	43.20	2.97	2.71	0.36	0.20	1.12	0.56
11JY1607	C6 RIL 238	261	LP	2	43.97	44.20	3.72	2.74	0.40	0.23	1.01	0.63
11JY1608	C6 RIL 239	262	LP	2	42.60	24.70	1.92	0.45	0.18	0.11	0.58	0.29
11JY1609	C6 RIL 241	263	LP	2	49.90	57.87	3.53	3.62	0.40	0.26	1.16	0.66
11JY1610	C6 RIL 243	264	LP	2	47.03	67.97	3.85	4.05	0.44	0.31	1.45	0.75
11JY1611	C6 RIL 245	265	LP	2	41.83	51.57	3.09	2.04	0.34	0.16	1.23	0.50
11JY1612	C6 RIL 249	266	LP	2	29.63	29.80	1.12	0.86	0.13	0.06	1.01	0.19
11JY1613	C6 RIL 253	267	LP	2	50.30	55.00	5.57	3.57	0.58	0.27	1.09	0.85
11JY1614	C6 RIL 258	268	LP	2	38.25	48.25	2.81	2.72	0.32	0.20	1.26	0.52
11JY1616	C6 RIL 261	269	LP	2	35.20	40.47	2.22	1.76	0.17	0.13	1.15	0.30
11JY1617	C6 RIL 262	270	LP	2	39.57	30.47	3.81	2.30	0.22	0.10	0.77	0.32
11JY1619	C6 RIL 265	271	LP	2	45.93	53.33	3.74	2.46	0.42	0.20	1.16	0.62
11JY1620	C6 RIL 267	272	LP	2	46.47	52.60	4.11	2.50	0.43	0.21	1.13	0.64
11JY1621	C6 RIL 268	273	LP	2	47.27	65.07	4.67	2.77	0.42	0.20	1.38	0.62
11JY1622	C6 RIL 270	274	LP	2	40.03	49.93	2.91	3.09	0.31	0.21	1.25	0.52
11JY1623	C6 RIL 272	275	LP	2	53.23	63.93	3.93	3.25	0.41	0.25	1.20	0.66
11JY1624	C6 RIL 273	276	LP	2	46.67	44.00	4.90	2.29	0.48	0.19	0.94	0.67
11JY1625	C6 RIL 275	277	LP	2	44.73	33.07	5.20	3.25	0.50	0.27	0.74	0.77
11JY1626	C6 RIL 276	278	LP	2	38.10	43.90	2.94	2.47	0.36	0.22	1.15	0.58
11JY1627	C6 RIL 282	279	LP	2	48.47	35.77	3.97	3.19	0.51	0.31	0.74	0.82
11JY1628	C6 RIL 283	280	LP	2	49.33	30.90	3.71	2.53	0.42	0.22	0.63	0.64
11JY1629	C6 RIL 284	281	LP	2	48.00	37.28	4.63	3.46	0.49	0.24	0.78	0.73
11JY1630	C6 RIL 286	282	LP	2	36.67	41.20	2.86	3.06	0.34	0.27	1.12	0.61

Seed ID	Acc	Gen	trt.	rep	SL	RL	SFW	RFW	SDW	RDW	RSR	TDM
11JY1631	C6 RIL 287	283	LP	2	43.57	60.93	3.53	2.63	0.40	0.20	1.40	0.60
11JY1632	C6 RIL 288	284	LP	2	42.60	33.55	2.79	0.89	0.20	0.08	0.79	0.28
11JY1633	C6 RIL 289	285	LP	2	44.13	33.97	3.79	2.72	0.38	0.22	0.77	0.60
11JY1634	C6 RIL 291	286	LP	2	45.50	60.20	3.57	3.16	0.40	0.24	1.32	0.64
11JY1635	C6 RIL 292	287	LP	2	36.07	15.10	2.23	1.82	0.27	0.18	0.42	0.45
11JY1636	C6 RIL 293	288	LP	2	47.00	52.10	4.05	3.41	0.48	0.29	1.11	0.77
11JY1637	C6 RIL 295	289	LP	2	40.07	39.00	3.59	2.11	0.39	0.19	0.97	0.58
11JY1638	C6 RIL 297	290	LP	2	35.60	30.20	2.80	1.71	0.30	0.15	0.85	0.45
11JY1639	C6 RIL 298	291	LP	2	43.20	38.67	4.56	3.92	0.47	0.27	0.90	0.74
11JY1640	C6 RIL 300	292	LP	2	38.13	35.93	3.53	1.96	0.34	0.17	0.94	0.51
11JY1641	C6 RIL 303	293	LP	2	40.83	29.70	3.37	2.27	0.32	0.16	0.73	0.48
11JY1642	C6 RIL 310	294	LP	2	50.00	58.90	4.32	3.40	0.39	0.25	1.18	0.64
11JY1643	C6 RIL 314	295	LP	2	38.70	29.40	1.99	0.69	0.14	0.50	0.76	0.64
11JY1644	C6 RIL 315	296	LP	2	52.90	38.93	6.27	4.04	0.62	0.36	0.74	0.98
11JY1645	C6 RIL 316	297	LP	2	25.40	42.20	1.46	0.82	0.11	0.07	1.66	0.18
11JY1646	C6 RIL 317	298	LP	2	32.23	37.13	1.64	1.29	0.19	0.11	1.15	0.30
11JY1647	C6 RIL 318	299	LP	2	36.10	39.93	2.72	2.68	0.27	0.19	1.11	0.46
11JY1648	C6 RIL 319	300	LP	2	33.00	30.00	2.46	1.76	0.26	0.13	0.91	0.39
11JY1649	C6 RIL 320	301	LP	2	38.57	54.87	2.97	3.04	0.37	0.27	1.42	0.64
11JY1650	C6 RIL 321	302	LP	2	39.70	35.35	3.05	1.94	0.14	0.08	0.89	0.22
11JY1651	C6 RIL 322	303	LP	2	39.37	37.07	4.02	2.27	0.45	0.23	0.94	0.68
11JY1652	C6 RIL 324	304	LP	2	34.20	42.37	3.49	3.62	0.37	0.25	1.24	0.62
11JY1653	C6 RIL 327	305	LP	2	54.63	41.67	5.45	3.89	0.51	0.31	0.76	0.82
11JY1654	C6 RIL 328	306	LP	2	50.83	53.23	3.53	2.04	0.32	0.15	1.05	0.47
11JY1655	C6 RIL 330	307	LP	2	45.83	45.67	4.32	3.40	0.37	0.21	1.00	0.58
11JY1656	C6 RIL 332	308	LP	2	56.80	59.47	6.58	4.84	0.50	0.30	1.05	0.80
11JY1657	C6 RIL 333	309	LP	2	44.30	32.80	2.60	1.73	0.28	0.17	0.74	0.45
11JY1658	C6 RIL 335	310	LP	2	37.50	41.50	1.69	2.10	0.28	0.19	1.11	0.47
11JY1659	C6 RIL 336	311	LP	2	35.00	32.00	2.17	2.87	0.26	0.21	0.91	0.47
11JY1660	C6 RIL 339	312	LP	2	31.17	38.33	2.25	1.80	0.25	0.15	1.23	0.40
11JY1661	C6 RIL 340	313	LP	2	31.67	28.33	1.60	1.35	0.24	0.12	0.89	0.36
11JY1662	C6 RIL 341	314	LP	2	42.00	36.00	2.28	2.38	0.31	0.26	0.86	0.57
11JY1663	C6 RIL 342	315	LP	2	32.67	53.00	1.84	1.50	0.20	0.14	1.62	0.34
11JY1664	C6 RIL 344	316	LP	2	35.33	26.33	2.13	1.69	0.25	0.20	0.75	0.45
11JY1665	C6 RIL 346	317	LP	2	48.93	35.60	4.10	2.07	0.38	0.21	0.73	0.59
11JY1666	C6 RIL 347	318	LP	2	40.00	57.67	2.77	1.67	0.34	0.18	1.44	0.52

Seed ID	Acc	Gen	trt.	rep	SL	RL	SFW	RFW	SDW	RDW	RSR	TDM
11JY1667	C6 RIL 349	319	LP	2	36.00	38.00	2.25	2.43	0.29	0.18	1.06	0.47
11JY1668	C6 RIL 352	320	LP	2	31.00	46.00	1.36	1.27	0.18	0.11	1.48	0.29
11JY1669	C6 RIL 354	321	LP	2	26.00	23.00	0.74	0.63	0.22	0.13	0.88	0.35
11JY1670	C6 RIL 355	322	LP	2	28.67	42.00	1.32	0.92	0.19	0.12	1.46	0.31
11JY1671	C6 RIL 356	323	LP	2	38.67	36.00	2.49	2.56	0.30	0.20	0.93	0.50
11JY1672	C6 RIL 358	324	LP	2	32.50	51.00	1.86	2.22	0.21	0.16	1.57	0.37
11JY1673	C6 RIL 361	325	LP	2	23.67	34.00	1.01	1.09	0.18	0.13	1.44	0.31
11JY1674	C6 RIL 362	326	LP	2	47.00	55.33	4.75	2.32	0.44	0.20	1.18	0.64
11JY1675	C6 RIL 364	327	LP	2	44.00	56.00	3.60	2.05	0.37	0.21	1.27	0.58
11JY1676	C6 RIL 366	328	LP	2	46.87	34.90	4.27	2.49	0.38	0.18	0.74	0.56
11JY1677	C6 RIL 368	329	LP	2	35.67	39.67	1.95	1.37	0.24	0.13	1.11	0.37
11JY1678	C6 RIL 372	330	LP	2	43.67	49.00	3.13	2.01	0.36	0.19	1.12	0.55
11JY1680	C6 RIL 379	331	LP	2	34.33	37.33	1.65	1.24	0.19	0.11	1.09	0.30
11JY1682	C6 RIL 382	332	LP	2	42.67	42.00	3.88	2.91	0.42	0.26	0.98	0.68
11JY1683	C6 RIL 388	333	LP	2	38.33	46.00	2.21	2.18	0.29	0.19	1.20	0.48
11JY1684	C6 RIL 391	334	LP	2	39.00	36.00	3.19	2.93	0.35	0.23	0.92	0.58
11JY1686	C6 RIL 394	335	LP	2	44.33	54.67	3.56	3.50	0.38	0.24	1.23	0.62
11JY1687	C6 RIL 395	336	LP	2	45.00	38.00	3.91	3.87	0.43	0.27	0.84	0.70
11JY1688	C6 RIL 398	337	LP	2	45.00	53.67	3.96	3.59	0.41	0.27	1.19	0.68
11JY1689	C6 RIL 400	338	LP	2	36.33	41.67	1.61	1.39	0.20	0.13	1.15	0.33
11JY2045	(CUBA/GUAD C1 F27-4-3-3-B-1-Bx[KILIMA ST94A]-30/MSV-03-2-10-B-2-B-B)-160-1-B-3-B	339	LP	2	39.08	56.06	3.19	1.77	0.30	0.17	1.43	0.47
11JY2047	[[MSRXPOOL9]C1F2-176-4-7-X-1-B/CML206]-5-2-3-1-BBBBB-B-B-B	340	LP	2	46.24	61.89	4.47	2.81	0.49	0.29	1.34	0.78
11JY2051	[CML199/[EV7992#/EV8449-SR]C1F2-334-1(OSU8i)-6-3-Sn]-B-23-2-2-B*4-B-B-B	341	LP	2	26.91	39.14	0.96	1.00	0.21	0.07	1.45	0.28
11JY2052	[CML312/[TUXPSEQ]C1F2/P49-SR]F2-45-3-2-1-BB//INTA-F2-192-2-1-1-1-BBBB]-1-5-1-1-1-BBB-B-B-B	342	LP	2	34.24	44.39	2.73	1.56	0.30	0.17	1.30	0.46
11JY2053	[CML312/CML445//[TUXPSEQ]C1F2/P49-SR]F2-45-3-2-1-BBB]-1-2-1-1-2-BBB-B-B-B	343	LP	2	28.24	35.14	1.96	1.01	0.26	0.08	1.24	0.34
11JY2054	[CML312/MAS[MSR/312]-109-3]-B-71-3-BBB-B-B-B	344	LP	2	42.08	32.06	3.02	1.72	0.35	0.16	0.76	0.51
11JY2055	[CML389/CML176]-B-29-2-2-B*5	345	LP	2	38.41	62.39	4.11	2.78	0.40	0.27	1.62	0.67

Seed ID	Acc	Gen	trt.	rep	SL	RL	SFW	RFW	SDW	RDW	RSR	TDM
11JY2056	[CML395/CML440//[LPSC3H144-1-2-2-2-4-#-BB/SC/ZM605#b-19-2-X]-1-2-X-1-1-BB]-1-2-1-1-B]-3-2-1-1-BBB-B-B-B	346	LP	2	34.74	54.89	3.13	2.13	0.31	0.22	1.58	0.52
11JY2060	[CML444/ZSR923S4BULK-2-2-X-X-X-X-1-BB]-1-1-1-2/CML441]-1-1-1-2-BBB-B-B-B	347	LP	2	40.24	62.81	4.91	3.09	0.62	0.35	1.56	0.96
11JY2061	[DRB-F2-180-2/DRB-3-4-1]-X-6-1-3-BB-2-BBBBBB-B-B-B	348	LP	2	28.58	48.48	2.10	1.78	0.28	0.15	1.70	0.43
11JY2063	[DTPWC8F31-4-2-1-6-B2/CML395//[CML445/ZM621B]-2-1-2-3-1-BB]-3-2-1-1-1-2-B-B-B	349	LP	2	42.74	50.39	3.69	2.02	0.41	0.23	1.18	0.63
11JY2064	[Ent320:92SEW2-77/[DMRESR-W]EarlySel-#I-2-4-B/CML386]-B-11-3-B-2-#-B*4	350	LP	2	29.24	49.81	1.85	1.90	0.28	0.15	1.70	0.43
11JY2065	[LZ956441/LZ966205]-B-3-4-4-B-5-BBBBB-B-B-B	351	LP	2	38.24	62.14	3.92	4.19	0.50	0.33	1.62	0.84
11JY2066	[MSRXPOOL9]C1F2-205-1(OSU23i)-5-3-X-X-1-B//EV7992/EV8449...-3-2-2-1-BBBBB-B-B-B	352	LP	2	25.24	30.14	1.48	1.36	0.17	0.05	1.19	0.23
11JY2067	[SYN-USAB2/SYN-ELIB2]-12-1-1-1-B*4-B-B-B	353	LP	2	42.41	55.06	3.64	2.23	0.40	0.21	1.30	0.61
11JY2070	02SADVE2B-#-42-1-1-1-1-B-B-B	354	LP	2	33.24	56.48	3.01	2.01	0.33	0.19	1.70	0.52
11JY2071	02SADVL2B-#-16-2-1-B-B-B	355	LP	2	30.58	55.48	2.03	2.62	0.29	0.23	1.81	0.52
11JY2075	20V-18	356	LP	2	35.91	38.81	2.82	2.37	0.45	0.27	1.08	0.72
11JY2076	622016-ZCN-2	357	LP	2	45.58	39.48	4.57	3.47	0.47	0.31	0.87	0.78
11JY2078	761BB2 BCox751B-B-1-1-B-B-B-B-B-B	358	LP	2	42.24	46.39	3.59	2.87	0.44	0.26	1.10	0.69
11JY2079	BRAZ 2309	359	LP	2	41.08	40.73	2.61	1.95	0.32	0.15	0.99	0.47
11JY2081	CL-04934 (P49C2H12-5-4xP23C2-11-1)-2-2-2-B*10	360	LP	2	32.74	33.73	3.41	2.67	0.36	0.25	1.03	0.61
11JY2083	CML103	361	LP	2	40.24	64.14	2.84	2.38	0.41	0.29	1.59	0.70
11JY2085	CML114	362	LP	2	43.24	44.89	4.27	3.37	0.50	0.33	1.04	0.83
11JY2086	CML115	363	LP	2	43.41	57.39	4.27	3.37	0.45	0.39	1.32	0.84
11JY2087	CML116	364	LP	2	37.58	40.48	2.50	1.37	0.26	0.12	1.08	0.38
11JY2088	CML118	365	LP	2	37.91	56.81	3.35	2.51	0.40	0.28	1.50	0.68
11JY2090	CML127	366	LP	2	33.58	53.81	2.87	2.48	0.30	0.16	1.60	0.45
11JY2091	CML130	367	LP	2	31.91	45.14	1.20	1.11	0.18	0.09	1.41	0.26
11JY2092	CML133	368	LP	2	37.24	57.48	2.77	3.91	0.38	0.33	1.54	0.70
11JY2093	CML134	369	LP	2	33.24	39.14	1.79	1.13	0.29	0.15	1.18	0.45
11JY2094	CML135	370	LP	2	29.58	43.48	2.92	3.68	0.40	0.33	1.47	0.73
11JY2102	CML169	371	LP	2	33.58	38.68	2.43	1.45	0.41	0.13	1.15	0.55
11JY2103	CML170	372	LP	2	42.91	58.68	3.92	4.17	0.41	0.29	1.37	0.70
11JY2107	CML192	373	LP	2	45.58	43.34	4.81	3.25	0.45	0.22	0.95	0.67

Seed ID	Acc	Gen	trt.	rep	SL	RL	SFW	RFW	SDW	RDW	RSR	TDM
11JY2108	CML20	374	LP	2	45.41	44.06	4.23	2.48	0.44	0.23	0.97	0.67
11JY2109	CML202	375	LP	2	38.58	55.34	3.73	2.61	0.38	0.22	1.43	0.60
11JY2110	CML206	376	LP	2	41.91	52.01	4.56	3.09	0.48	0.28	1.24	0.77
11JY2112	CML226	377	LP	2	32.24	40.34	2.05	1.15	0.24	0.09	1.25	0.34
11JY2114	CML229	378	LP	2	40.74	37.73	4.06	2.31	0.40	0.22	0.93	0.62
11JY2117	CML283	379	LP	2	40.00	44.01	3.20	2.09	0.34	0.18	1.10	0.52
11JY2122	CML290	380	LP	2	29.74	38.39	2.25	1.69	0.24	0.21	1.29	0.44
11JY2125	CML304	381	LP	2	39.58	40.01	4.40	3.49	0.45	0.22	1.01	0.68
11JY2126	CML31	382	LP	2	38.58	49.68	3.21	2.19	0.35	0.20	1.29	0.55
11JY2127	CML311/MBR C3 BC F23-1-2-1-B-B-B	383	LP	2	39.91	56.34	4.08	3.02	0.41	0.22	1.41	0.63
11JY2128	CML311/MBR C3 BC F3-1-1-1-B-B-B	384	LP	2	53.24	59.01	6.14	4.17	0.58	0.37	1.11	0.95
11JY2129	CML311/MBR C3 BC F3-1-1-2-B-B	385	LP	2	43.08	45.06	3.72	1.87	0.39	0.18	1.05	0.58
11JY2132	CML311/MBR C3 BC F43-2-1-1-B-B-B	386	LP	2	44.91	68.68	5.29	3.81	0.49	0.31	1.53	0.80
11JY2133	CML311/MBR C3 BC F65-1-2-2-B-B-B	387	LP	2	40.58	60.34	4.03	3.43	0.39	0.26	1.49	0.65
11JY2134	CML311/MBR C3 BC F95-2-2-1-B-B-B	388	LP	2	46.58	66.01	4.71	3.86	0.45	0.28	1.42	0.73
11JY2136	CML312SR	389	LP	2	40.24	63.68	3.71	2.94	0.36	0.24	1.58	0.60
11JY2137	CML312SRQ=[[(CLQ-RCWQ83xCML312SR)xCML312SR]xCML312SR)]-15-1-BBB	390	LP	2	46.24	53.68	4.57	3.47	0.80	0.27	1.16	1.07
11JY2140	CML322	391	LP	2	39.58	48.34	3.73	2.80	0.39	0.20	1.22	0.60
11JY2141	CML323	392	LP	2	51.91	53.68	5.99	3.93	0.61	0.29	1.03	0.90
11JY2142	CML325	393	LP	2	41.24	69.01	4.76	4.61	0.50	0.40	1.67	0.91
11JY2144	CML328	394	LP	2	37.91	51.68	4.47	3.12	0.43	0.21	1.36	0.64
11JY2146	CML338	395	LP	2	46.58	57.14	4.58	3.46	0.47	0.28	1.23	0.75
11JY2147	CML360	396	LP	2	47.91	48.48	5.41	3.36	0.51	0.26	1.01	0.78
11JY2148	CML361	397	LP	2	44.24	44.48	3.90	2.69	0.38	0.20	1.01	0.58
11JY2149	CML364	398	LP	2	37.34	49.98	4.16	2.14	0.39	0.17	1.34	0.56
11JY2150	CML380xMBR/MDR C3 BC F21-1-1-2-B-B-B-B-3-1-B-B-B	399	LP	2	33.91	66.14	4.06	3.68	0.43	0.37	1.95	0.80
11JY2151	CML384xMBR/MDR C3 BC F58-2-1-3-B-B-B-B-3-1-B-B-B	400	LP	2	38.68	38.64	4.34	2.80	0.70	0.30	1.00	1.00
11JY2152	CML389	401	LP	2	46.34	52.31	4.62	2.57	0.48	0.30	1.13	0.78
11JY2153	CML389/CML144//CML159//POOL15QPMSR-B-6-B-B	402	LP	2	50.84	49.64	4.54	2.82	0.66	0.19	0.98	0.85
11JY2154	CML40	403	LP	2	40.34	59.64	3.52	3.60	0.87	0.34	1.48	1.21
11JY2155	CML402	404	LP	2	41.01	60.31	4.81	3.56	0.68	0.39	1.47	1.07

Seed ID	Acc	Gen	trt.	rep	SL	RL	SFW	RFW	SDW	RDW	RSR	TDM
11JY2157	CML411	405	LP	2	39.34	47.64	3.29	2.15	0.31	0.20	1.21	0.51
11JY2160	CML423	406	LP	2	49.84	56.14	7.00	3.42	0.58	0.25	1.13	0.83
11JY2162	CML428	407	LP	2	37.68	56.98	2.83	2.46	0.27	0.20	1.51	0.48
11JY2163	CML430	408	LP	2	38.34	49.64	4.21	1.98	0.33	0.18	1.29	0.51
11JY2164	CML431	409	LP	2	36.84	45.64	3.49	2.30	0.27	0.15	1.24	0.42
11JY2165	CML432	410	LP	2	39.68	38.98	4.89	2.09	0.46	0.20	0.98	0.66
11JY2166	CML433	411	LP	2	31.84	40.64	3.06	1.76	0.29	0.18	1.28	0.47
11JY2168	CML445/CML144//CML159//POOL15QPMSR-B-55-B-B	412	LP	2	43.01	48.31	5.11	2.83	0.46	0.27	1.12	0.74
11JY2170	CML454	413	LP	2	39.34	69.98	3.44	3.00	0.35	0.29	1.78	0.65
11JY2172	CML468	414	LP	2	47.34	56.31	3.28	2.69	0.34	0.22	1.19	0.56
11JY2173	CML470	415	LP	2	47.68	45.64	4.81	3.33	0.49	0.30	0.96	0.78
11JY2176	CML479	416	LP	2	31.34	55.31	2.89	2.16	0.30	0.20	1.76	0.50
11JY2177	CML480	417	LP	2	45.68	49.64	3.99	2.48	0.42	0.25	1.09	0.67
11JY2180	CML496	418	LP	2	31.58	48.14	2.53	1.46	0.24	0.13	1.52	0.37
11JY2189	CML80	419	LP	2	48.34	49.31	5.26	2.88	0.45	0.25	1.02	0.70
11JY2192	CML94	420	LP	2	48.34	65.64	4.89	3.18	0.52	0.34	1.36	0.86
11JY2193	CML96	421	LP	2	42.01	37.64	3.76	1.63	0.35	0.16	0.90	0.51
11JY2194	CML99	422	LP	2	40.34	41.14	4.21	2.38	0.39	0.23	1.02	0.62
11JY2196	Cuba/GuadC3F125-2-2-1-B-B-B	423	LP	2	41.41	38.39	4.43	2.46	0.44	0.23	0.93	0.67
11JY2197	CY9169	424	LP	2	42.72	46.14	4.78	2.42	0.46	0.23	1.08	0.69
11JY2198	DTPW C9	425	LP	2	36.05	50.47	3.16	2.21	0.37	0.23	1.40	0.60
11JY2199	DTPWC9-F104-5-4-1-1-B-B-B	426	LP	2	50.05	65.47	5.89	3.00	0.60	0.32	1.31	0.93
11JY2200	DTPY C9	427	LP	2	34.58	50.14	3.74	3.23	0.39	0.27	1.45	0.66
11JY2201	DTPYC9-F46-1-2-1-2-B-B	428	LP	2	41.72	53.14	3.32	2.64	0.40	0.22	1.27	0.62
11JY2202	DTPYC9-F46-3-9-1-1-B-BTL-07B 6614-42	429	LP	2	30.72	42.81	2.73	1.41	0.28	0.13	1.39	0.41
11JY2204	Guad 6	430	LP	2	41.72	45.81	4.00	3.19	0.45	0.32	1.10	0.77
11JY2205	H-16	431	LP	2	50.72	58.14	5.00	2.82	0.42	0.27	1.15	0.69
11JY2207	La Posta Seq C7-F125-2-1-1-2-B-B-B	432	LP	2	32.38	48.81	3.60	2.72	0.38	0.21	1.51	0.59
11JY2211	La Posta Seq C7-F64-2-6-1-2-B-B-B	433	LP	2	45.91	65.81	4.64	3.61	0.51	0.34	1.43	0.84
11JY2216	La Posta Seq C7-F96-1-2-1-2-B-B	434	LP	2	37.58	45.14	4.49	3.04	0.43	0.26	1.20	0.69
11JY2218	LPSC7	435	LP	2	35.05	51.14	3.33	2.51	0.37	0.20	1.46	0.58
11JY2220	MAS[206/312]-23-2-1-1-B*6-B-B-B	436	LP	2	42.72	47.81	4.39	2.52	0.48	0.22	1.12	0.70
11JY2223	MBR C6 BC F234-1-B-#-1-1-B-B-B-B-B	437	LP	2	39.05	48.81	3.66	2.75	0.41	0.26	1.25	0.67
11JY2225	P402c2F2-695-2-BB-2-B*4-1-B	438	LP	2	34.72	45.31	2.88	2.46	0.29	0.15	1.31	0.45
11JY2228	P591c4 F55-2-2-2-B-B-B	439	LP	2	40.72	34.81	3.31	1.98	0.34	0.14	0.85	0.48

Seed ID	Acc	Gen	trt.	rep	SL	RL	SFW	RFW	SDW	RDW	RSR	TDM
11JY2229	P591c41y2GENF3-1-1-2-B-B-B	440	LP	2	29.72	38.81	2.73	1.56	0.30	0.12	1.31	0.42
11JY2230	PAZM 6053	441	LP	2	30.38	35.81	2.10	1.83	0.32	0.12	1.18	0.44
11JY2231	Pool 21 x Pool 22	442	LP	2	40.72	41.14	3.01	2.68	0.38	0.18	1.01	0.57
11JY2233	R15	443	LP	2	36.08	62.81	3.31	2.42	0.27	0.16	1.74	0.44
11JY2234	RDOM 330	444	LP	2	36.05	41.47	2.73	2.31	0.32	0.14	1.15	0.45
11JY2238	VL0512452	445	LP	2	41.05	52.81	3.58	2.78	0.46	0.25	1.29	0.71
11JY2239	VL0512464	446	LP	2	39.22	48.31	3.29	1.95	0.35	0.18	1.23	0.53
11JY2240	VL05128	447	LP	2	46.50	60.34	4.14	2.41	0.46	0.17	1.30	0.64
11JY2241	VL052	448	LP	2	36.24	35.48	3.10	2.06	0.30	0.17	0.98	0.47
11JY2243	VL05353	449	LP	2	52.24	53.81	5.75	4.26	0.51	0.29	1.03	0.80
11JY2244	VL054178	450	LP	2	40.83	64.01	2.90	2.58	0.41	0.20	1.57	0.61
11JY2246	VL054881	451	LP	2	43.16	47.34	3.33	2.84	0.45	0.23	1.10	0.69
11JY2248	VL0556	452	LP	2	36.16	64.34	2.28	2.27	0.34	0.20	1.78	0.54
11JY2249	VL05561	453	LP	2	41.16	61.01	3.25	2.35	0.41	0.19	1.48	0.60
11JY2250	VL0558	454	LP	2	37.58	70.48	4.65	3.25	0.50	0.35	1.88	0.85
11JY2252	VL05616	455	LP	2	39.41	53.14	4.24	2.63	0.39	0.26	1.35	0.65
11JY2255	VL056942	456	LP	2	37.08	66.81	3.54	2.02	0.33	0.23	1.80	0.55
11JY2259	VL062784	457	LP	2	37.74	42.81	3.40	1.91	0.30	0.18	1.13	0.48
11JY2260	VL062785	458	LP	2	38.08	54.48	3.75	2.33	0.35	0.24	1.43	0.59
11JY2262	VL06384	459	LP	2	35.58	58.81	3.27	2.14	0.36	0.17	1.65	0.53
11JY2263	ZM521B-66-4-1-1-BB-B-B-B	460	LP	2	42.41	45.39	3.76	2.21	0.36	0.20	1.07	0.56
11JY2264	川29♀	461	LP	2	43.41	48.39	3.74	1.84	0.39	0.20	1.11	0.59
11JY2265	慈溪白糯	462	LP	2	37.74	53.06	2.87	2.02	0.28	0.16	1.41	0.44
11JY2266	独紫	463	LP	2	46.41	50.39	4.68	2.66	0.39	0.23	1.09	0.62
11JY2268	交51	464	LP	2	40.08	43.73	3.58	2.11	0.34	0.17	1.09	0.51
11JY2269	双M9	465	LP	2	33.08	46.81	3.39	2.00	0.32	0.20	1.42	0.52
11JY2270	四川地方种质	466	LP	2	53.41	58.39	5.22	3.22	0.48	0.27	1.09	0.74
11JY2271	豫综BC15-2	467	LP	2	43.91	51.56	4.33	2.45	0.41	0.24	1.17	0.66
11JY2279	407	468	LP	2	33.74	33.06	2.65	1.41	0.24	0.14	0.98	0.38
11JY2280	412	469	LP	2	47.08	42.31	5.28	2.90	0.47	0.27	0.90	0.75
11JY2288	485	470	LP	2	43.91	55.81	5.08	3.53	0.47	0.25	1.27	0.72
11JY2290	495	471	LP	2	42.58	48.81	3.11	2.11	0.35	0.18	1.15	0.53
11JY2311	8001	472	LP	2	37.08	44.14	3.72	2.00	0.36	0.20	1.19	0.56
11JY2317	[(CML395/CML444)-B-4-1-3-1-B/CML395//DTPWC8F31-1-1-2-2]-5-1-2-2-BB-B-B-B	473	LP	2	29.58	59.81	2.61	1.95	0.24	0.17	2.02	0.42

Seed ID	Acc	Gen	trt.	rep	SL	RL	SFW	RFW	SDW	RDW	RSR	TDM
11JY2327	4F1	474	LP	2	40.58	43.14	4.76	3.73	0.48	0.35	1.06	0.83
11JY2356	B73	475	LP	2	39.91	33.06	3.46	1.60	0.39	0.16	0.83	0.55
11JY2391	E28	476	LP	2	39.91	51.14	5.11	3.14	0.55	0.30	1.28	0.85
11JY2394	ES40	477	LP	2	34.58	33.31	3.45	1.79	0.30	0.14	0.96	0.44
11JY2396	F42	478	LP	2	42.24	36.81	5.56	3.74	0.54	0.36	0.87	0.90
11JY2398	FR19	479	LP	2	35.58	52.81	3.64	2.83	0.37	0.24	1.48	0.61
11JY2426	MBNA	480	LP	2	35.50	42.01	2.66	2.28	0.33	0.16	1.18	0.49
11JY2434	NS701	481	LP	2	43.50	57.34	3.18	2.22	0.36	0.17	1.32	0.54
11JY2445	PHG83	482	LP	2	45.83	58.68	4.89	3.39	0.48	0.19	1.28	0.67
11JY2446	PHN47	483	LP	2	38.50	37.68	3.08	2.81	0.39	0.17	0.98	0.56
11JY2453	R09	484	LP	2	40.58	61.81	3.49	2.68	0.38	0.22	1.52	0.60
11JY2467	Va35	485	LP	2	36.41	56.14	3.95	2.75	0.31	0.23	1.54	0.54
11JY2470	W8304	486	LP	2	50.74	39.48	4.53	1.94	0.41	0.24	0.78	0.65
11JY2474	XZY364-1	487	LP	2	33.33	42.73	1.11	1.27	0.23	0.14	1.28	0.37
11JY2491	长3154	488	LP	2	36.49	57.89	3.23	2.83	0.46	0.33	1.59	0.79
11JY2498	丹3130	489	LP	2	30.91	35.14	3.02	1.66	0.30	0.20	1.14	0.50
11JY2500	丹340	490	LP	2	38.99	54.73	3.03	2.51	0.37	0.22	1.40	0.59
11JY2504	丹360	491	LP	2	42.74	52.48	4.23	2.57	0.42	0.23	1.23	0.64
11JY2522	辐746	492	LP	2	51.41	64.81	5.10	3.28	0.48	0.34	1.26	0.83
11JY2526	旱21	493	LP	2	44.41	52.14	4.07	2.51	0.36	0.24	1.17	0.59
11JY2547	吉419	494	LP	2	42.08	57.48	3.98	2.64	0.40	0.26	1.37	0.66
11JY2557	吉846	495	LP	2	50.83	54.68	5.75	3.72	0.58	0.23	1.08	0.82
11JY2563	冀研01-3-2-2-1-5-1	496	LP	2	37.83	43.68	4.54	2.12	0.52	0.17	1.15	0.70
11JY2570	金黄96C	497	LP	2	42.33	50.06	1.71	1.47	0.29	0.15	1.18	0.44
11JY2574	辽138	498	LP	2	41.16	58.68	4.79	3.42	0.52	0.24	1.43	0.77
11JY2587	辽孤001	499	LP	2	42.41	49.39	4.98	3.07	0.53	0.29	1.16	0.82
11JY2603	齐205	500	LP	2	47.41	39.06	4.47	2.20	0.46	0.25	0.82	0.71
11JY2606	齐310	501	LP	2	32.41	32.73	3.04	1.58	0.30	0.13	1.01	0.43
11JY2621	双105	502	LP	2	42.08	39.06	4.29	2.00	0.40	0.23	0.93	0.63
11JY2623	双741	503	LP	2	41.74	41.39	4.43	2.29	0.39	0.18	0.99	0.56
11JY2667	郑22	504	LP	2	41.83	56.68	4.28	2.90	0.48	0.27	1.35	0.75
11JY2672	郑29	505	LP	2	45.49	42.39	4.50	2.98	0.46	0.29	0.93	0.75
11JY2675	郑30	506	LP	2	41.83	47.68	3.81	2.20	0.43	0.16	1.14	0.59
11JY2677	郑35	507	LP	2	40.16	54.01	4.25	2.75	0.51	0.30	1.34	0.81

Seed ID	Acc	Gen	trt.	rep	SL	RL	SFW	RFW	SDW	RDW	RSR	TDM
11JY2697	综31	508	LP	2	48.16	49.01	4.52	2.32	0.50	0.22	1.02	0.72
12JY0001	C5 RIL P2	509	LP	2	50.00	63.39	2.35	2.67	0.42	0.22	1.27	0.64
12JY0002	C5 RIL P1	510	LP	2	32.99	41.06	1.63	2.50	0.32	0.23	1.24	0.55
12JY0015	[(SML*SMQPM)*(MTL*SMQPM)]F1S6-1-25-BB-1-B	511	LP	2	47.99	41.06	4.17	2.48	0.42	0.21	0.86	0.62
12JY0017	[CML159/[CML159/[MSRXPOOL9]C1F2-205-1(OSU23i)-5-3-X-X-1-BB]F2-3sx]-8-1-1-BB-1-B	512	LP	2	36.99	58.73	2.85	2.24	0.45	0.23	1.59	0.69
12JY0018	[CML198/LPSC3H144-1-2-2-2-2-#-BB]-1-4-1-1-4-B*4-B-B-B	513	LP	2	35.33	63.06	3.03	2.60	0.38	0.27	1.79	0.65
12JY0029	[DTPWC8F31-4-2-1-6/CML444//ZM521B-66-4-1-1-1-BB]-3-2-1-B-B-B	514	LP	2	33.99	59.39	3.30	2.52	0.37	0.21	1.75	0.57
12JY0040	18-599	515	LP	2	52.66	30.73	3.87	1.57	0.48	0.21	0.58	0.68
12JY0041	18-599(RED)	516	LP	2	41.33	37.39	2.75	1.17	0.41	0.18	0.90	0.59
12JY0108	CML330	517	LP	2	30.66	68.73	1.94	2.86	0.32	0.25	2.24	0.58
12JY0122	CML418	518	LP	2	31.33	35.73	0.74	1.25	0.23	0.12	1.14	0.35
12JY0146	CML504	519	LP	2	34.99	52.73	1.54	1.26	0.27	0.17	1.51	0.44
12JY0156	Cuba/Guad C3 F53-3-1-1-B-B-B	520	LP	2	43.33	53.06	2.93	2.11	0.34	0.24	1.22	0.58
12JY0164	ECA-MOROSR(BC1)F2-7-ECAVEE7/PL15QPMC7SRC1F2//POOL15QPMSR-B-4-B-B	521	LP	2	37.99	53.73	2.24	1.44	0.28	0.15	1.41	0.43
12JY0167	INTA-191-2-1-2-B*8-B-B-B	522	LP	2	37.33	43.06	1.60	1.39	0.26	0.14	1.15	0.40
12JY0173	La Posta Seq C7-F86-1-1-1-1-B-B-B	523	LP	2	22.99	32.39	0.27	0.94	0.13	0.12	1.41	0.25
12JY0185	P501SRc0-F2-47-3-1-1-B-B-B-B	524	LP	2	26.49	40.89	0.30	1.09	0.13	0.08	1.54	0.21
12JY0208	VL05610	525	LP	2	36.87	31.68	2.53	1.62	0.34	0.20	0.86	0.54
12JY0226	178	526	LP	2	30.33	19.39	1.09	1.44	0.27	0.17	0.64	0.45
12JY0228	273	527	LP	2	44.37	44.18	4.03	2.58	0.45	0.21	1.00	0.66
12JY0229	288	528	LP	2	32.99	31.39	1.25	1.42	0.24	0.14	0.95	0.38
12JY0240	764	529	LP	2	38.95	53.43	2.99	2.12	0.35	0.19	1.37	0.54
12JY0246	6103	530	LP	2	34.33	48.39	1.67	1.75	0.25	0.20	1.41	0.45
12JY0252	81565	531	LP	2	37.37	38.43	2.69	1.99	0.31	0.19	1.03	0.50
12JY0264	634-11511	532	LP	2	41.54	48.77	3.60	1.81	0.41	0.18	1.17	0.59
12JY0265	698-1	533	LP	2	38.66	40.73	3.67	2.40	0.33	0.21	1.05	0.54
12JY0308	FAPW	534	LP	2	46.08	38.81	4.50	2.44	0.42	0.23	0.84	0.65
12JY0322	LH132	535	LP	2	43.41	60.81	4.02	2.57	0.30	0.19	1.40	0.49
12JY0323	LH51	536	LP	2	33.41	37.14	2.98	2.38	0.22	0.16	1.11	0.38
12JY0325	LX9801	537	LP	2	42.41	60.14	3.07	2.60	0.22	0.16	1.42	0.38

Seed ID	Acc	Gen	trt.	rep	SL	RL	SFW	RFW	SDW	RDW	RSR	TDM
12JY0329	Mo17	538	LP	2	39.08	28.14	3.02	2.15	0.28	0.14	0.72	0.42
12JY0347	R08	539	LP	2	38.74	57.81	2.08	1.90	0.22	0.10	1.49	0.32
12JY0349	RP125	540	LP	2	37.66	51.98	3.70	2.31	0.36	0.20	1.38	0.56
12JY0365	Zhao835	541	LP	2	39.41	39.14	4.38	3.30	0.38	0.23	0.99	0.60
12JY0370	昌7-2	542	LP	2	38.29	53.18	2.52	1.75	0.32	0.17	1.39	0.50
12JY0383	丹598	543	LP	2	45.20	39.93	3.89	2.32	0.44	0.20	0.88	0.64
12JY0401	黄早四	544	LP	2	33.33	40.23	2.79	1.88	0.35	0.17	1.21	0.52
12JY0416	吉853	545	LP	2	39.08	50.14	3.35	1.98	0.22	0.12	1.28	0.35
12JY0443	南21-3	546	LP	2	48.70	50.77	4.37	2.82	0.43	0.25	1.04	0.68
12JY0450	齐319	547	LP	2	45.54	44.77	3.90	3.25	0.40	0.27	0.98	0.68
12JY0457	沈5003	548	LP	2	30.49	44.89	1.10	1.74	0.22	0.20	1.47	0.41
12JY0462	四287	549	LP	2	41.12	59.18	4.59	4.61	0.43	0.33	1.44	0.76
12JY0473	铁7922	550	LP	2	40.41	45.14	5.71	2.37	0.39	0.21	1.12	0.61
11JY1255	C5 RIL 2	1	NP	1	67.88	37.47	8.30	3.49	0.55	0.21	0.55	0.76
11JY1256	C5 RIL 3	2	NP	1	75.88	47.80	9.39	3.15	0.79	0.19	0.63	0.99
11JY1258	C5 RIL 5	3	NP	1	70.22	34.80	8.45	2.44	0.54	0.16	0.50	0.70
11JY1259	C5 RIL 6	4	NP	1	79.55	37.13	11.46	3.52	0.77	0.21	0.47	0.98
11JY1262	C5 RIL 10	5	NP	1	67.55	36.13	6.51	3.18	0.41	0.17	0.53	0.58
11JY1263	C5 RIL 11	6	NP	1	70.55	29.47	10.59	3.73	0.76	0.20	0.42	0.96
11JY1264	C5 RIL 12	7	NP	1	59.88	39.13	4.29	1.96	0.27	0.11	0.65	0.38
11JY1267	C5 RIL 17	8	NP	1	69.22	35.80	7.44	3.15	0.50	0.15	0.52	0.65
11JY1268	C5 RIL 18	9	NP	1	61.05	30.13	6.76	2.02	0.37	0.10	0.49	0.46
11JY1269	C5 RIL 19	10	NP	1	68.55	42.63	9.81	3.89	0.68	0.23	0.62	0.91
11JY1271	C5 RIL 21	11	NP	1	74.55	41.13	12.19	4.94	0.87	0.27	0.55	1.14
11JY1272	C5 RIL 24	12	NP	1	63.88	30.47	7.16	3.60	0.49	0.20	0.48	0.68
11JY1274	C5 RIL 26	13	NP	1	78.22	41.13	15.36	4.94	1.15	0.33	0.53	1.48
11JY1275	C5 RIL 27	14	NP	1	62.05	38.63	7.69	3.56	0.55	0.17	0.62	0.72
11JY1276	C5 RIL 28	15	NP	1	68.22	24.80	6.65	1.91	0.42	0.10	0.36	0.52
11JY1278	C5 RIL 30	16	NP	1	67.88	37.47	8.75	3.33	0.60	0.21	0.55	0.81
11JY1279	C5 RIL 31	17	NP	1	58.22	29.47	6.54	2.09	0.39	0.13	0.51	0.52
11JY1280	C5 RIL 32	18	NP	1	78.55	38.13	15.29	5.15	1.20	0.34	0.49	1.54
11JY1281	C5 RIL 34	19	NP	1	67.88	26.13	10.24	3.86	0.71	0.23	0.38	0.94
11JY1282	C5 RIL 36	20	NP	1	72.55	27.13	7.80	3.11	0.62	0.20	0.37	0.81
11JY1283	C5 RIL 38	21	NP	1	62.05	36.63	5.68	3.24	0.37	0.15	0.59	0.52
11JY1284	C5 RIL 39	22	NP	1	71.55	28.80	8.12	3.92	0.71	0.24	0.40	0.95
11JY1285	C5 RIL 40	23	NP	1	78.22	35.13	10.33	4.53	0.85	0.39	0.45	1.24

Seed ID	Acc	Gen	trt.	rep	SL	RL	SFW	RFW	SDW	RDW	RSR	TDM
11JY1286	C5 RIL 41	24	NP	1	73.22	28.47	8.58	3.46	0.70	0.22	0.39	0.92
11JY1287	C5 RIL 42	25	NP	1	80.05	41.63	9.51	4.01	0.75	0.21	0.52	0.96
11JY1288	C5 RIL 44	26	NP	1	75.05	35.63	5.75	2.41	0.40	0.12	0.47	0.51
11JY1289	C5 RIL 45	27	NP	1	79.55	43.13	7.75	2.97	0.51	0.21	0.54	0.72
11JY1290	C5 RIL 46	28	NP	1	71.55	27.80	7.57	2.98	0.54	0.16	0.39	0.70
11JY1292	C5 RIL 48	29	NP	1	76.55	37.63	11.65	3.39	0.86	0.23	0.49	1.09
11JY1293	C5 RIL 49	30	NP	1	79.88	46.13	11.04	4.77	1.03	0.33	0.58	1.36
11JY1299	C5 RIL 57	31	NP	1	73.88	31.13	6.30	3.81	0.63	0.20	0.42	0.83
11JY1301	C5 RIL 59	32	NP	1	73.88	34.80	7.94	3.38	0.55	0.16	0.47	0.71
11JY1305	C5 RIL 64	33	NP	1	78.55	47.13	7.99	3.59	0.76	0.30	0.60	1.07
11JY1310	C5 RIL 68	34	NP	1	77.55	43.63	12.04	5.54	0.81	0.36	0.56	1.17
11JY1312	C5 RIL 70	35	NP	1	70.22	37.13	11.81	2.93	0.84	0.21	0.53	1.05
11JY1315	C5 RIL 82	36	NP	1	66.05	37.80	9.59	2.64	0.98	0.39	0.57	1.37
11JY1316	C5 RIL 74	37	NP	1	61.55	29.80	9.47	2.66	0.79	0.20	0.48	0.99
11JY1318	C5 RIL 78	38	NP	1	60.88	35.13	8.32	2.41	0.54	0.17	0.58	0.71
11JY1319	C5 RIL 79	39	NP	1	71.88	39.47	12.30	4.14	1.02	0.31	0.55	1.32
11JY1320	C5 RIL 80	40	NP	1	57.05	31.80	5.79	2.09	0.48	0.17	0.56	0.66
11JY1323	C5 RIL 84	41	NP	1	65.05	42.30	11.10	3.59	0.85	0.24	0.65	1.09
11JY1324	C5 RIL 86	42	NP	1	56.55	36.80	7.51	2.28	0.57	0.19	0.65	0.76
11JY1326	C5 RIL 88	43	NP	1	64.88	41.13	9.36	2.83	0.77	0.21	0.63	0.98
11JY1327	C5 RIL 89	44	NP	1	66.05	41.30	11.07	3.09	0.78	0.27	0.63	1.06
11JY1328	C5 RIL 90	45	NP	1	61.55	35.30	9.10	2.49	0.65	0.20	0.57	0.85
11JY1329	C5 RIL 92	46	NP	1	70.05	38.30	15.56	4.21	0.97	0.29	0.55	1.26
11JY1333	C5 RIL 96	47	NP	1	75.22	41.47	13.27	3.31	1.02	0.24	0.55	1.26
11JY1334	C5 RIL 99	48	NP	1	68.55	38.80	10.65	3.40	0.84	0.24	0.57	1.08
11JY1336	C5 RIL 102	49	NP	1	57.22	30.47	7.23	1.96	0.75	0.21	0.53	0.96
11JY1337	C5 RIL 103	50	NP	1	63.88	32.80	10.10	3.21	0.87	0.24	0.51	1.11
11JY1339	C5 RIL 106	51	NP	1	69.55	27.80	12.01	4.45	1.04	0.35	0.40	1.39
11JY1341	C5 RIL 108	52	NP	1	70.55	43.30	5.79	2.80	0.43	0.15	0.61	0.58
11JY1349	C5 RIL 119	53	NP	1	75.55	43.30	10.14	4.10	0.87	0.23	0.57	1.10
11JY1350	C5 RIL 120	54	NP	1	74.55	33.63	7.59	3.53	0.57	0.17	0.45	0.74
11JY1351	C5 RIL 121	55	NP	1	80.05	49.80	13.83	5.26	0.99	0.28	0.62	1.27
11JY1352	C5 RIL 122	56	NP	1	76.22	43.30	12.29	4.21	0.86	0.18	0.57	1.04
11JY1354	C5 RIL 124	57	NP	1	84.88	45.30	13.88	4.62	1.12	0.27	0.53	1.39
11JY1356	C5 RIL 126	58	NP	1	78.05	39.30	8.07	2.81	0.61	0.16	0.50	0.77
11JY1357	C5 RIL 128	59	NP	1	70.88	41.30	10.03	3.18	0.76	0.20	0.58	0.95

Seed ID	Acc	Gen	trt.	rep	SL	RL	SFW	RFW	SDW	RDW	RSR	TDM
11JY1360	C5 RIL 131	60	NP	1	71.88	39.97	9.27	4.70	0.69	0.27	0.56	0.96
11JY1362	C5 RIL 133	61	NP	1	68.55	39.30	8.28	2.80	0.52	0.15	0.57	0.66
11JY1363	C5 RIL 134	62	NP	1	74.22	50.97	14.45	4.17	0.71	0.26	0.69	0.97
11JY1364	C5 RIL 135	63	NP	1	80.22	51.97	11.61	3.70	1.05	0.27	0.65	1.32
11JY1365	C5 RIL 137	64	NP	1	67.55	47.80	6.76	2.75	0.39	0.08	0.71	0.47
11JY1366	C5 RIL 138	65	NP	1	72.55	38.97	8.68	3.22	0.65	0.14	0.54	0.79
11JY1367	C5 RIL 139	66	NP	1	66.55	34.30	4.89	1.74	0.30	0.07	0.52	0.37
11JY1368	C5 RIL 140	67	NP	1	74.88	39.63	10.91	3.93	0.90	0.29	0.53	1.19
11JY1369	C5 RIL 141	68	NP	1	71.22	34.97	7.90	2.10	0.50	0.11	0.49	0.61
11JY1370	C5 RIL 142	69	NP	1	63.05	31.80	8.87	2.43	0.57	0.12	0.50	0.69
11JY1372	C5 RIL 144	70	NP	1	78.55	52.30	11.67	4.05	1.05	0.31	0.67	1.36
11JY1374	C5 RIL 146	71	NP	1	78.22	46.80	15.66	4.30	0.97	0.26	0.60	1.23
11JY1376	C5 RIL 150	72	NP	1	80.55	43.13	11.62	3.05	0.79	0.17	0.54	0.96
11JY1377	C5 RIL 151	73	NP	1	62.80	45.30	15.19	4.66	1.25	0.36	0.72	1.61
11JY1378	C5 RIL 152	74	NP	1	43.60	28.40	5.43	1.58	0.48	0.16	0.65	0.64
11JY1379	C5 RIL 153	75	NP	1	51.00	24.70	6.81	2.29	0.61	0.18	0.48	0.79
11JY1382	C5 RIL 156	76	NP	1	56.20	28.70	10.26	1.77	0.79	0.20	0.51	0.99
11JY1383	C5 RIL 157	77	NP	1	36.35	25.70	4.63	1.31	0.36	0.14	0.71	0.50
11JY1384	C5 RIL 158	78	NP	1	49.75	51.30	8.20	2.56	0.63	0.18	1.03	0.81
11JY1385	C5 RIL 159	79	NP	1	52.40	41.25	9.35	3.42	0.65	0.19	0.79	0.84
11JY1387	C5 RIL 161	80	NP	1	46.50	38.27	6.91	1.92	0.47	0.11	0.82	0.58
11JY1388	C5 RIL 162	81	NP	1	54.40	35.80	5.37	1.63	0.43	0.13	0.66	0.56
11JY1389	C5 RIL 163	82	NP	1	49.80	30.25	6.80	1.90	0.52	0.13	0.61	0.65
11JY1391	C5 RIL 165	83	NP	1	52.30	33.20	8.53	1.25	0.62	0.21	0.63	0.83
11JY1394	C5 RIL 169	84	NP	1	33.60	31.80	2.72	0.60	0.21	0.10	0.95	0.31
11JY1396	C5 RIL 171	85	NP	1	42.73	21.83	3.76	1.00	0.30	0.39	0.51	0.69
11JY1397	C5 RIL 172	86	NP	1	51.55	34.05	6.34	1.87	0.53	0.14	0.66	0.67
11JY1399	C5 RIL 174	87	NP	1	43.00	24.00	7.47	2.29	0.61	0.19	0.56	0.80
11JY1400	C5 RIL 176	88	NP	1	47.93	39.37	9.61	2.69	0.65	0.24	0.82	0.89
11JY1401	C5 RIL 177	89	NP	1	45.63	41.73	7.70	3.06	0.62	0.20	0.91	0.82
11JY1402	C5 RIL 178	90	NP	1	47.00	29.20	5.98	1.74	0.48	0.14	0.62	0.62
11JY1404	C5 RIL 182	91	NP	1	37.30	28.25	4.76	1.39	0.39	0.15	0.76	0.54
11JY1405	C5 RIL 183	92	NP	1	40.07	30.77	8.13	3.40	0.65	0.22	0.77	0.87
11JY1406	C5 RIL 184	93	NP	1	41.00	40.00	6.72	2.82	0.51	0.22	0.98	0.73
11JY1409	C5 RIL 187	94	NP	1	42.60	32.90	5.77	2.30	0.46	0.17	0.77	0.63
11JY1410	C5 RIL 188	95	NP	1	31.00	30.75	4.53	1.29	0.39	0.13	0.99	0.52

Seed ID	Acc	Gen	trt.	rep	SL	RL	SFW	RFW	SDW	RDW	RSR	TDM
11JY1412	C5 RIL 191	96	NP	1	52.40	45.23	12.56	3.61	1.05	0.36	0.86	1.41
11JY1413	C5 RIL 192	97	NP	1	44.50	40.30	6.63	2.28	0.56	0.21	0.91	0.77
11JY1414	C5 RIL 193	98	NP	1	34.10	46.97	4.93	1.32	0.41	0.17	1.38	0.58
11JY1416	C5 RIL 195	99	NP	1	44.20	28.30	6.58	1.02	0.66	0.25	0.64	0.91
11JY1418	C5 RIL 198	100	NP	1	37.85	36.85	5.94	1.95	0.48	0.20	0.97	0.68
11JY1420	C5 RIL 200	101	NP	1	43.50	31.00	9.21	1.42	0.80	0.19	0.71	0.99
11JY1421	C5 RIL 203	102	NP	1	37.15	33.25	6.27	1.77	0.47	0.19	0.90	0.66
11JY1422	C5 RIL 204	103	NP	1	41.30	39.90	6.89	1.88	0.54	0.17	0.97	0.71
11JY1423	C5 RIL 205	104	NP	1	44.80	42.97	7.10	1.64	0.59	0.16	0.96	0.75
11JY1424	C5 RIL 206	105	NP	1	52.67	45.57	8.33	2.86	0.67	0.24	0.87	0.91
11JY1425	C5 RIL 207	106	NP	1	34.30	31.80	4.04	2.15	0.29	0.14	0.93	0.43
11JY1426	C5 RIL 208	107	NP	1	33.40	32.40	9.18	4.96	0.54	0.29	0.97	0.83
11JY1428	C5 RIL 210	108	NP	1	43.67	34.33	10.69	5.33	0.77	0.28	0.79	1.05
11JY1429	C5 RIL 211	109	NP	1	64.88	34.13	2.39	1.68	0.46	0.12	0.53	0.58
11JY1429	C5 RIL 211	110	NP	1	27.10	28.70	12.35	1.45	0.25	0.11	1.06	0.36
11JY1431	C5 RIL 214	111	NP	1	39.80	34.85	8.95	4.29	0.62	0.24	0.88	0.86
11JY1433	C5 RIL 216	112	NP	1	28.55	27.05	3.93	1.83	0.32	0.15	0.95	0.47
11JY1434	C5 RIL 217	113	NP	1	30.80	21.00	3.67	1.21	0.25	0.10	0.68	0.35
11JY1435	C5 RIL 218	114	NP	1	31.00	25.00	4.74	2.00	0.40	0.12	0.81	0.52
11JY1437	C5 RIL 220	115	NP	1	48.80	28.30	8.53	3.43	0.65	0.23	0.58	0.88
11JY1440	C5 RIL 225	116	NP	1	30.60	31.40	3.63	1.53	0.22	0.10	1.03	0.32
11JY1442	C5 RIL 227	117	NP	1	36.77	35.43	9.97	4.47	0.65	0.25	0.96	0.90
11JY1444	C5 RIL 229	118	NP	1	38.50	23.20	10.92	3.00	0.45	0.27	0.60	0.72
11JY1454	C5 RIL 240	119	NP	1	34.60	29.00	7.35	2.20	0.48	0.15	0.84	0.63
11JY1455	C5 RIL 241	120	NP	1	36.35	23.30	5.17	1.43	0.36	0.13	0.64	0.49
11JY1456	C5 RIL 243	121	NP	1	44.05	38.75	10.04	3.64	0.75	0.23	0.88	0.98
11JY1459	C5 RIL P2	122	NP	1	38.90	43.35	7.36	2.97	0.57	0.20	1.11	0.77
11JY1460	C5 RIL P1	123	NP	1	40.00	31.50	7.83	3.32	0.54	0.21	0.79	0.75
11JY1461	C6 RIL 3	124	NP	1	50.70	26.30	5.09	0.80	0.44	0.13	0.52	0.57
11JY1462	C6 RIL 14	125	NP	1	57.10	40.77	7.33	2.73	0.53	0.14	0.71	0.67
11JY1463	C6 RIL 23	126	NP	1	68.00	43.67	15.06	5.57	1.07	0.36	0.64	1.43
11JY1464	C6 RIL 24	127	NP	1	47.67	38.33	10.18	4.28	0.97	0.30	0.80	1.27
11JY1465	C6 RIL 25	128	NP	1	61.50	52.50	19.50	6.47	1.30	0.42	0.85	1.72
11JY1466	C6 RIL 29	129	NP	1	51.83	41.13	12.28	4.11	0.94	0.26	0.79	1.20
11JY1468	C6 RIL 144	130	NP	1	80.50	43.40	26.44	8.23	2.15	0.63	0.54	2.78
11JY1469	C6 RIL 203	131	NP	1	64.97	35.30	18.08	8.13	1.19	0.41	0.54	1.60

Seed ID	Acc	Gen	trt.	rep	SL	RL	SFW	RFW	SDW	RDW	RSR	TDM
11JY1470	C6 RIL 212	132	NP	1	43.80	35.70	16.23	3.72	1.37	0.31	0.82	1.68
11JY1472	C6 RIL 219	133	NP	1	61.40	53.80	14.64	5.00	1.15	0.34	0.88	1.49
11JY1473	C6 RIL 239	134	NP	1	66.70	50.50	16.11	6.22	1.20	0.49	0.76	1.69
11JY1474	C6 RIL 243	135	NP	1	51.33	44.93	13.54	5.22	1.12	0.53	0.88	1.65
11JY1475	C6 RIL 272	136	NP	1	54.47	49.10	7.42	3.29	0.54	0.16	0.90	0.70
11JY1476	C6 RIL 273	137	NP	1	51.33	44.93	13.54	5.22	1.12	0.53	0.88	1.65
11JY1477	C6 RIL 283	138	NP	1	67.53	37.50	15.37	4.36	1.25	0.45	0.56	1.70
11JY1478	C6 RIL 293	139	NP	1	72.17	42.80	19.24	5.11	1.39	0.42	0.59	1.81
11JY1479	C6 RIL 298	140	NP	1	44.50	41.70	7.28	1.40	0.96	0.18	0.94	1.14
11JY1480	C6 RIL 303	141	NP	1	49.70	39.90	12.99	3.05	0.98	0.30	0.80	1.28
11JY1481	C6 RIL 327	142	NP	1	32.50	28.00	4.28	2.24	0.42	0.24	0.86	0.66
11JY1482	C6 RIL 341	143	NP	1	51.00	34.00	11.56	4.70	0.92	0.37	0.67	1.29
11JY1483	C6 RIL 348	144	NP	1	17.00	26.00	1.30	0.92	0.15	0.08	1.53	0.23
11JY1484	C6 RIL 402	145	NP	1	46.00	41.00	9.68	5.38	0.80	0.36	0.89	1.16
11JY1485	C6 RIL 403	146	NP	1	20.00	28.00	1.80	0.80	0.17	0.10	1.40	0.27
11JY1487	C6 RIL 5	147	NP	1	50.93	43.10	4.78	2.63	0.38	0.14	0.85	0.52
11JY1488	C6 RIL 7	148	NP	1	36.00	41.50	7.12	2.01	0.54	0.23	1.15	0.77
11JY1489	C6 RIL 10	149	NP	1	59.10	40.20	17.57	5.89	1.30	0.48	0.68	1.78
11JY1490	C6 RIL 15	150	NP	1	65.30	45.20	16.36	7.01	1.23	0.45	0.69	1.68
11JY1491	C6 RIL 19	151	NP	1	50.07	40.00	8.41	3.27	0.59	0.22	0.80	0.81
11JY1493	C6 RIL 21	152	NP	1	56.90	23.59	11.36	3.91	1.07	0.34	0.41	1.41
11JY1494	C6 RIL 23	153	NP	1	51.20	45.07	8.26	3.51	0.65	0.27	0.88	0.92
11JY1495	C6 RIL 24	154	NP	1	49.03	48.20	9.75	3.74	0.73	0.34	0.98	1.07
11JY1496	C6 RIL 25	155	NP	1	50.40	45.03	9.81	2.62	0.69	0.25	0.89	0.94
11JY1497	C6 RIL 28	156	NP	1	33.60	38.90	3.47	0.70	0.30	0.15	1.16	0.45
11JY1498	C6 RIL 29	157	NP	1	36.73	38.70	8.55	3.82	0.61	0.28	1.05	0.89
11JY1499	C6 RIL 30	158	NP	1	51.27	43.60	5.16	2.51	0.44	0.16	0.85	0.60
11JY1500	C6 RIL 31	159	NP	1	49.67	34.10	10.58	3.47	0.94	0.33	0.69	1.27
11JY1501	C6 RIL 32	160	NP	1	63.37	43.93	14.81	4.92	1.27	0.41	0.69	1.68
11JY1502	C6 RIL 33	161	NP	1	44.10	31.20	4.54	1.18	0.35	0.10	0.71	0.45
11JY1503	C6 RIL 36	162	NP	1	64.97	43.27	19.12	7.78	1.34	0.48	0.67	1.82
11JY1504	C6 RIL 39	163	NP	1	47.93	46.57	12.47	4.18	0.83	0.29	0.97	1.12
11JY1505	C6 RIL 40	164	NP	1	56.40	61.55	15.07	4.52	1.00	0.43	1.09	1.43
11JY1506	C6 RIL 42	165	NP	1	57.13	30.47	12.31	3.04	1.02	0.35	0.53	1.37
11JY1508	C6 RIL 47	166	NP	1	63.67	47.77	17.15	4.15	1.13	0.41	0.75	1.54
11JY1509	C6 RIL 49	167	NP	1	47.70	30.30	11.81	1.45	0.15	0.12	0.64	0.27

Seed ID	Acc	Gen	trt.	rep	SL	RL	SFW	RFW	SDW	RDW	RSR	TDM
11JY1510	C6 RIL 50	168	NP	1	60.97	45.67	18.69	4.47	1.19	0.44	0.75	1.63
11JY1511	C6 RIL 52	169	NP	1	52.00	30.50	7.15	3.50	0.60	0.19	0.59	0.79
11JY1512	C6 RIL 53	170	NP	1	74.30	60.60	21.05	5.64	1.40	0.44	0.82	1.84
11JY1513	C6 RIL 57	171	NP	1	59.20	49.85	16.09	4.75	1.23	0.46	0.84	1.69
11JY1514	C6 RIL 58	172	NP	1	70.10	36.40	16.60	7.42	1.93	0.75	0.52	2.68
11JY1515	C6 RIL 59	173	NP	1	52.45	37.25	9.14	1.85	0.63	0.22	0.71	0.85
11JY1516	C6 RIL 60	174	NP	1	45.20	42.25	8.15	2.96	0.57	0.24	0.93	0.81
11JY1517	C6 RIL 64	175	NP	1	62.97	27.97	10.72	2.42	0.96	0.23	0.44	1.19
11JY1518	C6 RIL 65	176	NP	1	70.17	51.03	19.07	4.34	1.04	0.32	0.73	1.36
11JY1519	C6 RIL 66	177	NP	1	67.90	48.55	18.64	6.31	1.19	0.36	0.72	1.55
11JY1520	C6 RIL 68	178	NP	1	40.20	43.50	6.47	2.36	0.44	0.13	1.08	0.57
11JY1521	C6 RIL 69	179	NP	1	53.40	48.95	12.15	4.46	0.77	0.28	0.92	1.05
11JY1522	C6 RIL 70	180	NP	1	53.70	45.67	8.76	2.69	0.46	0.12	0.85	0.58
11JY1523	C6 RIL 72	181	NP	1	55.70	58.60	18.06	6.12	1.15	0.41	1.05	1.56
11JY1524	C6 RIL 74	182	NP	1	57.40	51.10	13.36	4.35	0.82	0.31	0.89	1.13
11JY1525	C6 RIL 76	183	NP	1	48.67	46.53	10.01	2.74	0.68	0.32	0.96	1.00
11JY1527	C6 RIL 79	184	NP	1	48.25	39.85	8.02	1.75	0.62	0.23	0.83	0.85
11JY1528	C6 RIL 83	185	NP	1	65.03	29.00	14.73	3.80	1.13	0.36	0.45	1.49
11JY1529	C6 RIL 85	186	NP	1	56.30	47.87	14.27	3.54	0.99	0.33	0.85	1.32
11JY1530	C6 RIL 86	187	NP	1	59.60	40.25	13.25	2.06	0.93	0.24	0.68	1.17
11JY1531	C6 RIL 91	188	NP	1	63.97	37.43	21.37	4.51	1.68	0.41	0.59	2.09
11JY1532	C6 RIL 94	189	NP	1	56.87	38.60	10.71	2.72	0.76	0.17	0.68	0.93
11JY1533	C6 RIL 95	190	NP	1	56.93	48.07	8.52	3.14	0.61	0.19	0.84	0.80
11JY1534	C6 RIL 97	191	NP	1	48.70	46.53	6.76	1.90	0.42	0.11	0.96	0.53
11JY1535	C6 RIL 103	192	NP	1	21.65	35.35	1.22	0.70	0.10	0.07	1.63	0.17
11JY1536	C6 RIL 104	193	NP	1	48.97	44.93	8.20	2.99	0.59	0.18	0.92	0.77
11JY1537	C6 RIL 107	194	NP	1	43.27	25.77	4.62	1.19	0.31	0.08	0.60	0.39
11JY1538	C6 RIL 108	195	NP	1	54.00	42.90	10.75	2.97	0.69	0.20	0.79	0.89
11JY1539	C6 RIL 109	196	NP	1	30.85	29.30	1.98	1.10	0.16	0.07	0.95	0.23
11JY1540	C6 RIL 112	197	NP	1	62.07	33.57	10.22	3.34	0.62	0.17	0.54	0.79
11JY1541	C6 RIL 113	198	NP	1	60.53	39.43	14.13	3.16	0.74	0.28	0.65	1.02
11JY1542	C6 RIL 115	199	NP	1	65.87	29.10	13.16	2.47	0.86	0.26	0.44	1.12
11JY1543	C6 RIL 116	200	NP	1	39.35	36.30	4.40	0.56	0.34	0.13	0.92	0.47
11JY1544	C6 RIL 117	201	NP	1	55.60	40.35	6.79	0.87	0.53	0.19	0.73	0.72
11JY1545	C6 RIL 120	202	NP	1	65.07	53.10	8.87	2.76	0.62	0.16	0.82	0.78
11JY1546	C6 RIL 121	203	NP	1	66.65	37.35	16.03	1.77	1.31	0.37	0.56	1.68

Seed ID	Acc	Gen	trt.	rep	SL	RL	SFW	RFW	SDW	RDW	RSR	TDM
11JY1547	C6 RIL 123	204	NP	1	65.90	37.05	19.02	3.25	1.50	0.56	0.56	2.06
11JY1548	C6 RIL 124	205	NP	1	48.70	44.10	9.04	0.99	0.70	0.22	0.91	0.92
11JY1549	C6 RIL 127	206	NP	1	51.20	33.25	9.32	2.74	0.73	0.20	0.65	0.93
11JY1550	C6 RIL 131	207	NP	1	54.30	47.60	10.87	4.07	0.88	0.30	0.88	1.18
11JY1551	C6 RIL 134	208	NP	1	60.70	35.15	11.18	8.46	0.95	0.27	0.58	1.22
11JY1552	C6 RIL 135	209	NP	1	63.17	37.43	15.71	4.82	1.10	0.30	0.59	1.40
11JY1553	C6 RIL 138	210	NP	1	61.43	43.03	11.76	3.81	0.87	0.28	0.70	1.15
11JY1554	C6 RIL 139	211	NP	1	37.90	33.40	5.47	1.41	0.32	0.11	0.88	0.43
11JY1555	C6 RIL 140	212	NP	1	59.30	38.20	12.23	3.10	1.64	0.38	0.64	2.02
11JY1556	C6 RIL 141	213	NP	1	45.77	38.93	4.27	0.90	0.34	0.10	0.85	0.44
11JY1557	C6 RIL 142	214	NP	1	80.80	46.55	23.10	6.38	1.61	0.46	0.58	2.07
11JY1558	C6 RIL 143	215	NP	1	61.40	42.80	9.70	1.81	0.74	0.18	0.70	0.92
11JY1560	C6 RIL 146	216	NP	1	51.03	37.93	6.77	1.41	0.49	0.15	0.74	0.64
11JY1561	C6 RIL 147	217	NP	1	28.60	27.50	2.30	0.36	0.19	0.10	0.96	0.29
11JY1562	C6 RIL 148	218	NP	1	47.50	45.35	6.93	1.67	0.57	0.23	0.95	0.80
11JY1563	C6 RIL 150	219	NP	1	51.80	40.60	5.41	1.00	0.44	0.15	0.78	0.59
11JY1565	C6 RIL 153	220	NP	1	45.25	43.10	8.53	1.32	0.65	0.20	0.95	0.85
11JY1566	C6 RIL 156	221	NP	1	56.50	42.20	5.06	0.55	0.33	0.10	0.75	0.43
11JY1567	C6 RIL 160	222	NP	1	65.07	43.33	11.87	4.52	0.93	0.26	0.67	1.19
11JY1568	C6 RIL 161	223	NP	1	57.55	42.00	9.48	3.19	0.70	0.21	0.73	0.91
11JY1569	C6 RIL 162	224	NP	1	56.25	36.75	7.73	2.55	0.60	0.17	0.65	0.77
11JY1570	C6 RIL 163	225	NP	1	46.57	33.60	6.93	2.57	0.49	0.13	0.72	0.62
11JY1571	C6 RIL 164	226	NP	1	56.70	37.70	9.21	2.50	0.58	0.23	0.66	0.81
11JY1572	C6 RIL 169	227	NP	1	58.83	34.47	7.13	1.22	0.61	0.12	0.59	0.73
11JY1573	C6 RIL 171	228	NP	1	65.80	30.53	9.78	1.39	0.72	0.14	0.46	0.86
11JY1574	C6 RIL 172	229	NP	1	63.70	53.10	12.62	4.19	0.89	0.33	0.83	1.22
11JY1576	C6 RIL 174	230	NP	1	36.50	30.10	2.97	1.02	0.23	0.10	0.82	0.33
11JY1577	C6 RIL 175	231	NP	1	42.40	30.20	3.85	0.75	0.38	0.12	0.71	0.50
11JY1578	C6 RIL 176	232	NP	1	40.35	26.45	4.16	0.71	0.35	0.13	0.66	0.48
11JY1579	C6 RIL 179	233	NP	1	31.30	37.25	2.82	0.33	0.24	0.08	1.19	0.32
11JY1580	C6 RIL 181	234	NP	1	50.65	37.05	5.17	0.78	0.54	0.12	0.73	0.66
11JY1581	C6 RIL 184	235	NP	1	70.40	33.20	7.90	1.83	0.31	0.10	0.47	0.41
11JY1582	C6 RIL 186	236	NP	1	52.90	41.10	10.61	5.35	0.74	0.30	0.78	1.04
11JY1583	C6 RIL 187	237	NP	1	32.60	35.50	2.99	0.56	0.30	0.13	1.09	0.43
11JY1584	C6 RIL 188	238	NP	1	45.50	48.05	6.60	1.88	0.53	0.21	1.06	0.74
11JY1585	C6 RIL 189	239	NP	1	53.40	37.60	9.51	1.86	0.75	0.25	0.70	1.00

Seed ID	Acc	Gen	trt.	rep	SL	RL	SFW	RFW	SDW	RDW	RSR	TDM
11JY1586	C6 RIL 190	240	NP	1	60.47	34.83	6.72	1.40	0.52	0.14	0.58	0.66
11JY1587	C6 RIL 192	241	NP	1	43.10	35.30	3.69	0.80	0.29	0.10	0.82	0.39
11JY1588	C6 RIL 193	242	NP	1	59.10	46.65	7.70	2.42	0.52	0.15	0.79	0.67
11JY1589	C6 RIL 203	243	NP	1	63.87	44.60	9.01	2.03	0.59	0.15	0.70	0.74
11JY1590	C6 RIL 204	244	NP	1	51.40	39.35	6.38	1.95	0.44	0.13	0.77	0.57
11JY1591	C6 RIL 205	245	NP	1	45.33	37.63	4.45	0.94	0.26	0.10	0.83	0.36
11JY1592	C6 RIL 206	246	NP	1	42.60	46.50	3.54	1.00	0.26	0.10	1.09	0.36
11JY1593	C6 RIL 207	247	NP	1	54.73	31.93	9.22	3.51	0.72	0.24	0.58	0.96
11JY1594	C6 RIL 211	248	NP	1	45.33	28.63	3.95	0.74	0.27	0.10	0.63	0.37
11JY1595	C6 RIL 212	249	NP	1	60.90	39.47	9.02	1.76	0.61	0.15	0.65	0.76
11JY1596	C6 RIL 217	250	NP	1	40.60	28.90	3.59	0.35	0.30	0.11	0.71	0.41
11JY1597	C6 RIL 218	251	NP	1	57.75	35.70	7.75	1.47	0.52	0.12	0.62	0.64
11JY1598	C6 RIL 219	252	NP	1	67.25	52.10	10.48	2.17	0.79	0.18	0.77	0.97
11JY1599	C6 RIL 220	253	NP	1	50.05	35.40	5.53	0.92	0.55	0.21	0.71	0.76
11JY1600	C6 RIL 225	254	NP	1	57.47	29.33	11.27	3.01	0.80	0.21	0.51	1.01
11JY1601	C6 RIL 230	255	NP	1	56.80	60.55	9.55	3.74	0.66	0.24	1.07	0.90
11JY1602	C6 RIL 231	256	NP	1	62.35	47.40	10.00	4.03	0.79	0.26	0.76	1.05
11JY1603	C6 RIL 232	257	NP	1	59.60	51.90	12.43	5.33	0.90	0.33	0.87	1.23
11JY1604	C6 RIL 234	258	NP	1	70.90	51.25	14.60	5.90	1.08	0.31	0.72	1.39
11JY1605	C6 RIL 235	259	NP	1	63.70	44.70	12.91	5.82	0.94	0.37	0.70	1.31
11JY1606	C6 RIL 236	260	NP	1	52.35	47.20	9.08	5.74	0.67	0.28	0.90	0.95
11JY1607	C6 RIL 238	261	NP	1	53.73	38.07	10.74	2.39	0.70	0.16	0.71	0.86
11JY1608	C6 RIL 239	262	NP	1	41.80	30.20	1.64	0.37	0.55	0.14	0.72	0.69
11JY1609	C6 RIL 241	263	NP	1	66.23	44.67	15.26	6.44	1.03	0.39	0.67	1.42
11JY1610	C6 RIL 243	264	NP	1	62.40	58.13	12.20	6.77	0.95	0.38	0.93	1.33
11JY1611	C6 RIL 245	265	NP	1	47.35	51.75	8.34	2.73	0.62	0.16	1.09	0.78
11JY1612	C6 RIL 249	266	NP	1	44.64	35.03	3.68	1.35	0.30	0.10	0.78	0.40
11JY1613	C6 RIL 253	267	NP	1	58.83	53.53	12.82	3.29	1.00	0.25	0.91	1.25
11JY1614	C6 RIL 258	268	NP	1	60.00	44.20	9.60	3.39	0.64	0.22	0.74	0.86
11JY1616	C6 RIL 261	269	NP	1	47.50	39.60	5.29	1.37	0.41	0.13	0.83	0.54
11JY1617	C6 RIL 262	270	NP	1	46.33	33.13	6.95	2.47	0.51	0.17	0.72	0.68
11JY1619	C6 RIL 265	271	NP	1	66.80	55.20	12.31	4.00	0.90	0.20	0.83	1.10
11JY1620	C6 RIL 267	272	NP	1	58.47	55.87	10.12	3.42	0.79	0.18	0.96	0.97
11JY1621	C6 RIL 268	273	NP	1	47.27	44.37	1.04	3.83	0.34	0.13	0.94	0.47
11JY1622	C6 RIL 270	274	NP	1	58.67	36.07	10.78	4.52	0.77	0.23	0.61	1.00
11JY1623	C6 RIL 272	275	NP	1	66.60	50.30	12.54	6.14	0.92	0.32	0.76	1.24

Seed ID	Acc	Gen	trt.	rep	SL	RL	SFW	RFW	SDW	RDW	RSR	TDM
11JY1624	C6 RIL 273	276	NP	1	37.80	32.67	3.76	0.36	0.25	0.10	0.86	0.35
11JY1625	C6 RIL 275	277	NP	1	63.70	31.07	13.39	4.47	1.10	0.33	0.49	1.43
11JY1626	C6 RIL 276	278	NP	1	52.50	42.43	8.55	3.45	0.83	0.27	0.81	1.10
11JY1627	C6 RIL 282	279	NP	1	75.80	41.53	23.40	9.89	2.14	0.74	0.55	2.88
11JY1628	C6 RIL 283	280	NP	1	69.47	36.93	13.48	3.11	1.14	0.35	0.53	1.49
11JY1629	C6 RIL 284	281	NP	1	55.75	39.45	10.41	4.20	0.83	0.24	0.71	1.07
11JY1630	C6 RIL 286	282	NP	1	61.00	48.40	15.62	6.82	1.43	0.47	0.79	1.90
11JY1631	C6 RIL 287	283	NP	1	57.70	61.80	9.73	4.55	0.78	0.23	1.07	1.01
11JY1632	C6 RIL 288	284	NP	1	34.70	33.20	1.75	0.54	0.16	0.06	0.96	0.22
11JY1633	C6 RIL 289	285	NP	1	67.30	51.85	17.40	7.52	1.69	0.54	0.77	2.23
11JY1634	C6 RIL 291	286	NP	1	67.30	51.85	17.40	7.52	1.69	0.54	0.77	2.23
11JY1635	C6 RIL 292	287	NP	1	44.70	44.15	4.91	2.20	0.45	0.14	0.99	0.59
11JY1636	C6 RIL 293	288	NP	1	69.77	50.57	16.86	6.35	1.45	0.54	0.72	1.99
11JY1637	C6 RIL 295	289	NP	1	66.80	54.07	14.93	2.66	1.17	0.48	0.81	1.65
11JY1638	C6 RIL 297	290	NP	1	71.00	36.47	14.86	4.05	1.03	0.27	0.51	1.30
11JY1639	C6 RIL 298	291	NP	1	46.45	45.50	3.99	1.44	0.68	0.16	0.98	0.84
11JY1640	C6 RIL 300	292	NP	1	62.60	36.93	16.45	4.72	1.33	0.36	0.59	1.69
11JY1641	C6 RIL 303	293	NP	1	56.17	56.17	9.71	3.86	0.86	0.31	1.00	1.17
11JY1642	C6 RIL 310	294	NP	1	68.50	64.00	17.98	8.04	1.48	0.61	0.93	2.09
11JY1643	C6 RIL 314	295	NP	1	38.85	28.40	2.29	0.57	0.21	0.06	0.73	0.27
11JY1644	C6 RIL 315	296	NP	1	69.50	40.50	15.31	6.30	1.00	0.39	0.58	1.39
11JY1645	C6 RIL 316	297	NP	1	66.50	59.00	16.56	5.78	1.08	0.38	0.89	1.46
11JY1646	C6 RIL 317	298	NP	1	71.00	46.00	17.13	7.46	1.22	0.46	0.65	1.68
11JY1647	C6 RIL 318	299	NP	1	66.75	39.00	16.45	5.97	1.48	0.52	0.58	2.00
11JY1648	C6 RIL 319	300	NP	1	46.00	47.00	5.54	1.72	0.42	0.18	1.02	0.60
11JY1649	C6 RIL 320	301	NP	1	61.00	48.00	11.36	4.74	0.82	0.33	0.79	1.15
11JY1650	C6 RIL 321	302	NP	1	37.00	40.33	3.12	0.87	0.24	0.08	1.09	0.32
11JY1651	C6 RIL 322	303	NP	1	66.50	54.00	16.35	5.27	1.16	0.45	0.81	1.61
11JY1652	C6 RIL 324	304	NP	1	57.53	43.80	6.79	1.90	0.54	0.46	0.76	1.00
11JY1653	C6 RIL 327	305	NP	1	58.57	45.33	10.60	3.40	0.76	0.26	0.77	1.02
11JY1654	C6 RIL 328	306	NP	1	50.27	32.03	4.12	1.58	0.35	0.12	0.64	0.47
11JY1655	C6 RIL 330	307	NP	1	71.17	53.40	14.81	6.38	1.01	0.38	0.75	1.39
11JY1656	C6 RIL 332	308	NP	1	76.00	55.77	14.91	6.85	1.03	0.39	0.73	1.42
11JY1657	C6 RIL 333	309	NP	1	65.00	49.73	11.92	5.33	1.01	0.36	0.77	1.37
11JY1658	C6 RIL 335	310	NP	1	53.00	49.33	9.96	4.50	0.91	0.29	0.93	1.20
11JY1659	C6 RIL 336	311	NP	1	36.50	34.50	7.37	4.70	0.60	0.30	0.95	0.90

Seed ID	Acc	Gen	trt.	rep	SL	RL	SFW	RFW	SDW	RDW	RSR	TDM
11JY1660	C6 RIL 339	312	NP	1	46.50	42.33	7.67	3.35	0.57	0.20	0.91	0.77
11JY1661	C6 RIL 340	313	NP	1	53.75	44.50	10.30	4.07	0.96	0.34	0.83	1.30
11JY1662	C6 RIL 341	314	NP	1	28.67	34.67	3.34	1.35	0.28	0.10	1.21	0.38
11JY1663	C6 RIL 342	315	NP	1	55.75	48.50	10.31	3.70	0.85	0.29	0.87	1.14
11JY1664	C6 RIL 344	316	NP	1	28.17	33.67	3.20	1.60	0.27	0.13	1.20	0.40
11JY1665	C6 RIL 346	317	NP	1	54.23	34.83	5.62	2.05	0.47	0.17	0.64	0.64
11JY1666	C6 RIL 347	318	NP	1	47.25	49.00	6.80	2.77	0.57	0.22	1.04	0.79
11JY1667	C6 RIL 349	319	NP	1	51.67	48.33	9.86	3.41	0.89	0.29	0.94	1.18
11JY1668	C6 RIL 352	320	NP	1	40.67	38.00	3.29	1.08	0.29	0.14	0.93	0.43
11JY1669	C6 RIL 354	321	NP	1	19.00	31.00	1.13	0.62	0.13	0.11	1.63	0.24
11JY1670	C6 RIL 355	322	NP	1	44.00	52.00	7.92	3.14	0.73	0.31	1.18	1.04
11JY1671	C6 RIL 356	323	NP	1	54.67	43.33	10.84	5.14	1.01	0.41	0.79	1.42
11JY1672	C6 RIL 358	324	NP	1	49.70	48.00	8.90	3.27	0.71	0.21	0.97	0.92
11JY1673	C6 RIL 361	325	NP	1	35.67	40.67	4.68	1.52	0.46	0.13	1.14	0.59
11JY1674	C6 RIL 362	326	NP	1	68.83	54.67	19.14	5.55	1.31	0.41	0.79	1.72
11JY1675	C6 RIL 364	327	NP	1	72.00	55.33	20.36	5.67	1.34	0.38	0.77	1.72
11JY1676	C6 RIL 366	328	NP	1	46.67	34.30	4.76	2.87	0.40	0.18	0.73	0.58
11JY1677	C6 RIL 368	329	NP	1	64.00	46.00	9.81	3.06	0.75	0.25	0.72	1.00
11JY1678	C6 RIL 372	330	NP	1	64.00	54.33	28.27	7.39	2.02	0.52	0.85	2.54
11JY1680	C6 RIL 379	331	NP	1	48.83	33.67	5.69	1.63	0.44	0.13	0.69	0.57
11JY1682	C6 RIL 382	332	NP	1	71.33	39.67	20.12	6.46	1.21	0.43	0.56	1.64
11JY1683	C6 RIL 388	333	NP	1	65.83	34.33	14.14	3.73	1.12	0.37	0.52	1.49
11JY1684	C6 RIL 391	334	NP	1	47.50	39.00	8.65	3.21	0.65	0.22	0.82	0.87
11JY1686	C6 RIL 394	335	NP	1	68.50	58.33	16.28	6.00	1.22	0.49	0.85	1.71
11JY1687	C6 RIL 395	336	NP	1	65.67	38.00	16.31	5.11	1.06	0.32	0.58	1.38
11JY1688	C6 RIL 398	337	NP	1	68.33	43.67	17.19	5.01	1.26	0.43	0.64	1.69
11JY1689	C6 RIL 400	338	NP	1	69.50	44.00	11.73	5.06	0.86	0.43	0.63	1.29
11JY2045	(CUBA/GUAD C1 F27-4-3-3-B-1-Bx[KILIMA ST94A]-30/MSV-03-2-10-B-2-B-B)-160-1-B-3-B	339	NP	1	57.68	50.48	11.66	2.78	0.67	0.19	0.88	0.85
11JY2047	[[MSRXPOOL9]C1F2-176-4-7-X-1-B/CML206]-5-2-3-1-BBBBB-B-B-B	340	NP	1	70.02	61.15	20.77	4.52	1.34	0.34	0.87	1.68
11JY2051	[CML199/[EV7992#/EV8449-SR]C1F2-334-1(OSU8i)-6-3-Sn]-B-23-2-2-B*4-B-B-B	341	NP	1	43.16	48.03	10.51	3.65	0.85	0.17	1.11	1.02
11JY2052	[CML312/[TUXPSEQ]C1F2/P49-SR]F2-45-3-2-1-BB//INTA-F2-192-2-1-1-1-BBBB]-1-5-1-1-1-BBB-B-B-B	342	NP	1	61.85	36.15	13.92	3.19	0.84	0.21	0.58	1.06

Seed ID	Acc	Gen	trt.	rep	SL	RL	SFW	RFW	SDW	RDW	RSR	TDM
11JY2053	[CML312/CML445//[TUXPSEQ]C1F2/P49-SR]F2-45-3-2-1-BBB]-1-2-1-1-2-BBB-B-B-B	343	NP	1	48.79	35.97	6.97	2.36	0.48	0.08	0.74	0.57
11JY2054	[CML312/MAS[MSR/312]-109-3]-B-71-3-BBB-B-B-B	344	NP	1	59.02	46.82	13.20	3.76	0.82	0.26	0.79	1.07
11JY2055	[CML389/CML176]-B-29-2-2-B*5	345	NP	1	55.02	55.15	15.45	4.15	0.88	0.25	1.00	1.12
11JY2056	[CML395/CML440//[LPSC3H144-1-2-2-2-4-#-BB/SC/ZM605#b-19-2-X]-1-2-X-1-1-BB]-1-2-1-1-B]-3-2-1-1-BBB-B-B-B	346	NP	1	33.79	34.97	2.70	1.37	0.21	0.08	1.03	0.29
11JY2060	[CML444/ZSR923S4BULK-2-2-X-X-X-X-1-BB]-1-1-1-2/CML441]-1-1-1-2-BBB-B-B-B	347	NP	1	35.46	36.63	1.60	0.78	0.13	0.04	1.03	0.17
11JY2061	[DRB-F2-180-2/DRB-3-4-1]-X-6-1-3-BB-2-BBBBBB-B-B-B	348	NP	1	45.79	38.63	9.05	2.57	0.74	0.17	0.84	0.91
11JY2063	[DTPWC8F31-4-2-1-6-B2/CML395//[CML445/ZM621B]-2-1-2-3-1-BB]-3-2-1-1-1-2-B-B-B	349	NP	1	56.35	45.48	11.67	2.72	0.66	0.20	0.81	0.86
11JY2064	[Ent320:92SEW2-77/[DMRESR-W]EarlySel-#I-2-4-B/CML386]-B-11-3-B-2-#-B*4	350	NP	1	39.46	35.97	4.03	1.62	0.37	0.09	0.91	0.46
11JY2065	[LZ956441/LZ966205]-B-3-4-4-B-5-BBBBB-B-B-B	351	NP	1	50.13	48.30	7.59	3.12	0.68	0.19	0.96	0.87
11JY2066	[MSRXPOOL9]C1F2-205-1(OSU23i)-5-3-X-X-1-B//EV7992/EV8449...-3-2-2-1-BBBBB-B-B-B	352	NP	1	44.63	45.97	6.77	1.96	0.69	0.16	1.03	0.85
11JY2067	[SYN-USAB2/SYN-ELIB2]-12-1-1-1-B*4-B-B-B	353	NP	1	51.46	60.30	9.45	4.02	0.69	0.19	1.17	0.87
11JY2070	02SADVE2B-#-42-1-1-1-1-B-B-B	354	NP	1	43.13	53.97	4.03	1.55	0.26	0.09	1.25	0.35
11JY2071	02SADVL2B-#-16-2-1-B-B-B	355	NP	1	39.13	43.63	3.02	1.42	0.21	0.03	1.12	0.24
11JY2075	20V-18	356	NP	1	56.79	34.97	11.03	2.20	0.78	0.11	0.62	0.89
11JY2076	622016-ZCN-2	357	NP	1	58.79	41.63	10.45	3.10	0.76	0.19	0.71	0.95
11JY2078	761BB2 BCox751B-B-1-1-B-B-B-B-B-B	358	NP	1	50.46	39.63	8.95	2.74	0.85	0.24	0.79	1.08
11JY2079	BRAZ 2309	359	NP	1	50.79	46.97	4.15	1.52	0.36	0.09	0.92	0.45
11JY2081	CL-04934 (P49C2H12-5-4xP23C2-11-1)-2-2-2-B*10	360	NP	1	53.02	42.48	14.38	3.86	0.87	0.24	0.80	1.10
11JY2083	CML103	361	NP	1	62.13	58.30	9.92	3.57	0.78	0.21	0.94	0.99
11JY2085	CML114	362	NP	1	44.79	38.30	5.37	1.97	0.34	0.09	0.86	0.43
11JY2086	CML115	363	NP	1	48.46	38.97	4.80	1.85	0.32	0.06	0.80	0.38
11JY2087	CML116	364	NP	1	48.13	37.63	5.07	1.41	0.36	0.08	0.78	0.44
11JY2088	CML118	365	NP	1	41.46	43.30	3.78	2.18	0.34	0.06	1.04	0.40
11JY2090	CML127	366	NP	1	41.79	44.30	5.05	2.11	0.44	0.07	1.06	0.51
11JY2091	CML130	367	NP	1	52.79	53.30	6.24	2.09	0.51	0.08	1.01	0.58
11JY2092	CML133	368	NP	1	50.79	44.97	8.45	3.41	0.68	0.24	0.89	0.91

Seed ID	Acc	Gen	trt.	rep	SL	RL	SFW	RFW	SDW	RDW	RSR	TDM
11JY2093	CML134	369	NP	1	49.79	38.30	5.25	1.25	0.50	0.11	0.77	0.61
11JY2094	CML135	370	NP	1	53.46	35.63	11.69	3.87	0.92	0.32	0.67	1.24
11JY2102	CML169	371	NP	1	53.83	34.80	7.64	2.68	0.56	0.26	0.65	0.82
11JY2103	CML170	372	NP	1	50.49	35.47	8.85	3.71	0.74	0.23	0.70	0.98
11JY2107	CML192	373	NP	1	67.16	42.80	12.89	3.60	0.90	0.33	0.64	1.24
11JY2108	CML20	374	NP	1	48.16	34.13	5.79	2.23	0.49	0.16	0.71	0.65
11JY2109	CML202	375	NP	1	49.49	31.80	7.20	2.16	0.50	0.17	0.64	0.67
11JY2110	CML206	376	NP	1	53.49	40.80	9.49	3.07	0.67	0.21	0.76	0.88
11JY2112	CML226	377	NP	1	44.83	31.47	4.54	1.45	0.36	0.12	0.70	0.48
11JY2114	CML229	378	NP	1	57.16	28.80	11.81	5.70	0.82	0.31	0.50	1.13
11JY2117	CML283	379	NP	1	48.83	31.80	6.93	2.23	0.52	0.18	0.65	0.70
11JY2122	CML290	380	NP	1	52.33	40.30	7.63	3.29	0.54	0.23	0.77	0.76
11JY2125	CML304	381	NP	1	61.49	33.47	13.33	5.73	1.03	0.29	0.54	1.32
11JY2126	CML31	382	NP	1	57.83	42.30	7.70	2.89	0.60	0.19	0.73	0.79
11JY2127	CML311/MBR C3 BC F23-1-2-1-B-B-B	383	NP	1	53.83	33.80	7.61	2.64	0.49	0.15	0.63	0.64
11JY2128	CML311/MBR C3 BC F3-1-1-1-B-B-B	384	NP	1	66.83	49.47	15.11	5.84	1.07	0.41	0.74	1.48
11JY2129	CML311/MBR C3 BC F3-1-1-2-B-B	385	NP	1	53.33	41.30	6.73	2.53	0.51	0.17	0.77	0.68
11JY2132	CML311/MBR C3 BC F43-2-1-1-B-B-B	386	NP	1	60.83	43.80	12.89	4.99	0.92	0.27	0.72	1.19
11JY2133	CML311/MBR C3 BC F65-1-2-2-B-B-B	387	NP	1	45.16	33.80	6.09	2.41	0.50	0.14	0.75	0.64
11JY2134	CML311/MBR C3 BC F95-2-2-1-B-B-B	388	NP	1	58.83	39.13	10.72	4.13	0.74	0.25	0.67	0.99
11JY2136	CML312SR	389	NP	1	51.33	39.30	7.38	3.01	0.57	0.21	0.77	0.78
11JY2137	CML312SRQ=[[(CLQ-RCWQ83xCML312SR)xCML312SR]xCML312SR)]-15-1-BBB	390	NP	1	49.16	29.80	6.40	2.88	0.49	0.19	0.61	0.68
11JY2140	CML322	391	NP	1	42.49	31.47	6.53	3.91	0.48	0.22	0.74	0.70
11JY2141	CML323	392	NP	1	52.83	47.47	8.05	2.98	0.71	0.22	0.90	0.93
11JY2142	CML325	393	NP	1	61.83	48.47	9.13	4.51	0.76	0.27	0.78	1.02
11JY2144	CML328	394	NP	1	49.16	35.13	8.08	2.09	0.64	0.19	0.71	0.84
11JY2146	CML338	395	NP	1	54.49	47.97	11.43	2.12	0.93	0.18	0.88	1.11
11JY2147	CML360	396	NP	1	45.49	36.97	8.01	2.26	0.62	0.14	0.81	0.76
11JY2148	CML361	397	NP	1	60.83	42.63	13.43	3.65	0.88	0.23	0.70	1.10
11JY2149	CML364	398	NP	1	50.74	41.98	9.49	3.04	0.57	0.18	0.83	0.75
11JY2150	CML380xMBR/MDR C3 BC F21-1-1-2-B-B-B-B-3-1-B-B-B	399	NP	1	48.41	47.32	7.00	2.50	0.50	0.14	0.98	0.64
11JY2151	CML384xMBR/MDR C3 BC F58-2-1-3-B-B-B-B-3-1-B-B-B	400	NP	1	62.41	32.98	12.68	2.51	0.98	0.20	0.53	1.17

Seed ID	Acc	Gen	trt.	rep	SL	RL	SFW	RFW	SDW	RDW	RSR	TDM
11JY2152	CML389	401	NP	1	62.08	38.65	12.59	3.48	0.95	0.21	0.62	1.16
11JY2153	CML389/CML144//CML159//POOL15QPMSR-B-6-B-B	402	NP	1	49.24	39.98	6.29	2.33	0.36	0.16	0.81	0.52
11JY2154	CML40	403	NP	1	54.41	44.65	8.56	3.03	0.60	0.24	0.82	0.84
11JY2155	CML402	404	NP	1	57.41	45.65	11.05	3.18	0.97	0.31	0.80	1.28
11JY2157	CML411	405	NP	1	61.74	50.32	13.08	5.35	0.69	0.33	0.81	1.02
11JY2160	CML423	406	NP	1	57.08	38.65	16.34	4.16	1.39	0.38	0.68	1.77
11JY2162	CML428	407	NP	1	43.74	33.98	4.57	1.68	0.38	0.11	0.78	0.48
11JY2163	CML430	408	NP	1	45.24	36.48	6.41	1.70	0.46	0.14	0.81	0.60
11JY2164	CML431	409	NP	1	52.74	31.65	7.78	1.95	0.53	0.10	0.60	0.63
11JY2165	CML432	410	NP	1	51.08	24.65	10.10	1.69	0.78	0.16	0.48	0.94
11JY2166	CML433	411	NP	1	37.74	23.98	4.56	1.76	0.30	0.13	0.64	0.43
11JY2168	CML445/CML144//CML159//POOL15QPMSR-B-55-B-B	412	NP	1	48.08	31.98	9.91	2.75	0.77	0.18	0.67	0.95
11JY2170	CML454	413	NP	1	49.24	34.48	7.59	3.39	0.81	0.27	0.70	1.07
11JY2172	CML468	414	NP	1	71.74	35.98	13.21	4.40	1.16	0.32	0.50	1.48
11JY2173	CML470	415	NP	1	57.41	34.65	10.76	4.24	0.80	0.29	0.60	1.08
11JY2176	CML479	416	NP	1	41.41	45.65	5.60	1.94	0.45	0.18	1.10	0.63
11JY2177	CML480	417	NP	1	53.08	28.65	9.78	3.21	0.75	0.26	0.54	1.01
11JY2180	CML496	418	NP	1	52.74	47.98	5.58	1.80	0.52	0.14	0.91	0.66
11JY2189	CML80	419	NP	1	66.08	45.32	14.00	3.82	1.16	0.29	0.69	1.45
11JY2192	CML94	420	NP	1	62.08	37.65	13.50	3.69	1.21	0.37	0.61	1.58
11JY2193	CML96	421	NP	1	39.74	29.32	2.93	0.28	0.27	0.09	0.74	0.36
11JY2194	CML99	422	NP	1	42.74	32.32	3.53	1.29	0.28	0.12	0.76	0.40
11JY2196	Cuba/GuadC3F125-2-2-1-B-B-B	423	NP	1	49.58	44.88	11.89	2.58	0.69	0.26	0.91	0.95
11JY2197	CY9169	424	NP	1	51.22	29.06	11.44	2.27	0.73	0.16	0.57	0.89
11JY2198	DTPW C9	425	NP	1	55.05	34.06	10.69	3.52	0.83	0.24	0.62	1.07
11JY2199	DTPWC9-F104-5-4-1-1-B-B-B	426	NP	1	70.88	51.06	17.01	2.97	0.95	0.33	0.72	1.28
11JY2200	DTPY C9	427	NP	1	41.55	37.06	7.99	3.10	0.59	0.19	0.89	0.78
11JY2201	DTPYC9-F46-1-2-1-2-B-B	428	NP	1	66.22	58.73	11.60	3.34	0.76	0.18	0.89	0.94
11JY2202	DTPYC9-F46-3-9-1-1-B-BTL-07B 6614-42	429	NP	1	42.55	34.06	7.30	2.07	0.45	0.12	0.80	0.57
11JY2204	Guad 6	430	NP	1	55.55	51.73	12.36	4.95	0.77	0.30	0.93	1.07
11JY2205	H-16	431	NP	1	64.22	52.06	12.69	4.43	0.92	0.29	0.81	1.21
11JY2207	La Posta Seq C7-F125-2-1-1-2-B-B-B	432	NP	1	50.55	39.73	10.64	4.26	0.71	0.25	0.79	0.97
11JY2211	La Posta Seq C7-F64-2-6-1-2-B-B-B	433	NP	1	53.55	45.06	11.97	4.14	0.99	0.29	0.84	1.28
11JY2216	La Posta Seq C7-F96-1-2-1-2-B-B	434	NP	1	46.22	46.06	9.60	3.41	0.66	0.23	1.00	0.89

Seed ID	Acc	Gen	trt.	rep	SL	RL	SFW	RFW	SDW	RDW	RSR	TDM
11JY2218	LPSC7	435	NP	1	62.55	58.06	11.05	3.40	0.72	0.18	0.93	0.90
11JY2220	MAS[206/312]-23-2-1-1-B*6-B-B-B	436	NP	1	51.22	31.39	9.99	2.36	0.63	0.16	0.61	0.80
11JY2223	MBR C6 BC F234-1-B-#-1-1-B-B-B-B-B	437	NP	1	52.55	38.06	9.83	3.12	0.64	0.19	0.72	0.84
11JY2225	P402c2F2-695-2-BB-2-B*4-1-B	438	NP	1	48.22	40.73	8.10	2.48	0.50	0.15	0.84	0.66
11JY2228	P591c4 F55-2-2-2-B-B-B	439	NP	1	50.55	37.73	8.86	2.68	0.57	0.17	0.75	0.74
11JY2229	P591c41y2GENF3-1-1-2-B-B-B	440	NP	1	46.55	44.06	7.90	2.11	0.49	0.13	0.95	0.62
11JY2230	PAZM 6053	441	NP	1	38.55	35.06	7.32	2.09	0.54	0.16	0.91	0.70
11JY2231	Pool 21 x Pool 22	442	NP	1	53.05	26.56	7.77	2.38	0.51	0.15	0.50	0.65
11JY2233	R15	443	NP	1	62.55	42.06	10.98	2.71	0.71	0.19	0.67	0.90
11JY2234	RDOM 330	444	NP	1	44.05	29.06	7.09	2.17	0.47	0.13	0.66	0.60
11JY2238	VL0512452	445	NP	1	52.22	32.06	10.60	2.86	0.83	0.22	0.61	1.05
11JY2239	VL0512464	446	NP	1	59.55	37.73	13.20	3.18	0.91	0.24	0.63	1.15
11JY2240	VL05128	447	NP	1	53.02	42.18	11.61	2.91	0.69	0.21	0.80	0.90
11JY2241	VL052	448	NP	1	57.35	34.18	13.13	2.90	0.84	0.21	0.60	1.05
11JY2243	VL05353	449	NP	1	60.35	39.85	16.27	5.39	1.09	0.40	0.66	1.50
11JY2244	VL054178	450	NP	1	47.35	40.18	9.80	2.28	0.53	0.15	0.85	0.68
11JY2246	VL054881	451	NP	1	53.02	38.85	10.69	2.67	0.67	0.16	0.73	0.83
11JY2248	VL0556	452	NP	1	49.35	47.18	11.72	3.15	0.73	0.26	0.96	0.99
11JY2249	VL05561	453	NP	1	41.68	37.18	8.88	2.32	0.46	0.17	0.89	0.63
11JY2250	VL0558	454	NP	1	50.68	45.52	11.94	2.70	0.61	0.19	0.90	0.80
11JY2252	VL05616	455	NP	1	55.83	39.13	11.13	2.82	0.63	0.20	0.70	0.83
11JY2255	VL056942	456	NP	1	59.83	57.80	13.84	3.18	0.82	0.25	0.97	1.08
11JY2259	VL062784	457	NP	1	51.83	36.80	10.10	2.27	0.52	0.17	0.71	0.69
11JY2260	VL062785	458	NP	1	63.16	42.80	13.07	2.88	0.76	0.21	0.68	0.97
11JY2262	VL06384	459	NP	1	49.99	32.97	10.24	2.73	0.74	0.12	0.66	0.85
11JY2263	ZM521B-66-4-1-1-BB-B-B-B	460	NP	1	55.74	45.38	11.95	2.81	0.61	0.24	0.81	0.85
11JY2264	川29♀	461	NP	1	56.24	44.55	12.32	2.56	0.66	0.20	0.79	0.86
11JY2265	慈溪白糯	462	NP	1	49.74	46.88	10.83	2.53	0.50	0.17	0.94	0.68
11JY2266	独紫	463	NP	1	63.58	54.55	14.39	3.46	0.76	0.26	0.86	1.02
11JY2268	交51	464	NP	1	58.58	43.22	13.02	2.77	0.69	0.23	0.74	0.92
11JY2269	双M9	465	NP	1	52.24	41.88	10.99	2.48	0.56	0.21	0.80	0.76
11JY2270	四川地方种质	466	NP	1	59.91	46.22	13.51	3.26	0.74	0.26	0.77	1.00
11JY2271	豫综BC15-2	467	NP	1	54.24	41.22	11.25	2.57	0.58	0.22	0.76	0.80
11JY2279	407	468	NP	1	49.74	38.38	10.84	2.30	0.51	0.19	0.77	0.71
11JY2280	412	469	NP	1	39.99	32.97	6.26	1.84	0.44	0.10	0.82	0.54
11JY2288	485	470	NP	1	56.49	44.97	15.42	6.04	1.10	0.32	0.80	1.41

Seed ID	Acc	Gen	trt.	rep	SL	RL	SFW	RFW	SDW	RDW	RSR	TDM
11JY2290	495	471	NP	1	52.99	36.97	9.12	2.86	0.66	0.16	0.70	0.81
11JY2311	8001	472	NP	1	58.49	42.13	13.17	3.05	0.75	0.20	0.72	0.95
11JY2317	[(CML395/CML444)-B-4-1-3-1-B/CML395//DTPWC8F31-1-1-2-2]-5-1-2-2-BB-B-B-B	473	NP	1	41.99	48.97	6.58	2.14	0.47	0.08	1.17	0.54
11JY2327	4F1	474	NP	1	52.83	43.63	11.22	3.98	0.73	0.19	0.83	0.92
11JY2356	B73	475	NP	1	78.88	51.30	13.20	3.41	0.79	0.24	0.65	1.03
11JY2391	E28	476	NP	1	54.46	38.97	9.22	1.85	0.67	0.12	0.72	0.80
11JY2394	ES40	477	NP	1	47.49	42.47	9.32	2.40	0.68	0.11	0.89	0.79
11JY2396	F42	478	NP	1	60.46	39.63	17.46	4.59	1.24	0.30	0.66	1.54
11JY2398	FR19	479	NP	1	46.13	45.97	5.92	2.22	0.38	0.06	1.00	0.45
11JY2426	MBNA	480	NP	1	55.35	39.18	11.97	3.45	0.67	0.22	0.71	0.89
11JY2434	NS701	481	NP	1	62.02	42.85	11.78	2.73	0.61	0.17	0.69	0.78
11JY2445	PHG83	482	NP	1	53.68	42.52	11.29	2.80	0.63	0.17	0.79	0.80
11JY2446	PHN47	483	NP	1	53.85	39.85	13.44	3.33	0.80	0.23	0.74	1.03
11JY2453	R09	484	NP	1	51.49	34.97	11.20	3.28	0.94	0.23	0.68	1.16
11JY2467	Va35	485	NP	1	54.83	43.80	12.29	3.18	0.62	0.22	0.80	0.84
11JY2470	W8304	486	NP	1	63.16	38.80	12.71	2.65	0.75	0.22	0.61	0.97
11JY2474	XZY364-1	487	NP	1	70.05	43.47	2.52	2.64	0.33	0.12	0.62	0.46
11JY2491	长3154	488	NP	1	51.83	53.80	10.83	2.88	0.60	0.22	1.04	0.82
11JY2498	丹3130	489	NP	1	50.83	40.13	9.36	2.55	0.50	0.21	0.79	0.71
11JY2500	丹340	490	NP	1	71.88	45.13	5.58	2.31	0.40	0.13	0.63	0.53
11JY2504	丹360	491	NP	1	60.83	45.80	11.99	2.74	0.70	0.20	0.75	0.91
11JY2522	辐746	492	NP	1	66.49	43.13	12.88	2.97	0.77	0.21	0.65	0.98
11JY2526	旱21	493	NP	1	65.83	48.47	14.21	3.38	0.85	0.27	0.74	1.12
11JY2547	吉419	494	NP	1	76.83	63.13	19.77	4.73	1.30	0.40	0.82	1.69
11JY2557	吉846	495	NP	1	58.68	45.85	14.07	3.89	0.87	0.25	0.78	1.12
11JY2563	冀研01-3-2-2-1-5-1	496	NP	1	51.35	43.85	11.15	2.70	0.55	0.14	0.85	0.69
11JY2570	金黄96C	497	NP	1	62.68	54.52	15.31	3.51	0.83	0.21	0.87	1.04
11JY2574	辽138	498	NP	1	53.68	43.18	13.15	3.70	0.67	0.19	0.80	0.86
11JY2587	辽孤001	499	NP	1	58.91	45.55	15.05	2.96	0.96	0.28	0.77	1.24
11JY2603	齐205	500	NP	1	59.24	40.55	12.29	2.68	0.75	0.23	0.68	0.98
11JY2606	齐310	501	NP	1	48.91	40.55	10.84	2.41	0.51	0.19	0.83	0.71
11JY2621	双105	502	NP	1	54.91	38.88	11.45	2.50	0.61	0.19	0.71	0.80
11JY2623	双741	503	NP	1	56.24	45.88	12.74	3.03	0.71	0.20	0.82	0.91

Seed ID	Acc	Gen	trt.	rep	SL	RL	SFW	RFW	SDW	RDW	RSR	TDM
11JY2667	郑22	504	NP	1	59.02	50.18	16.53	4.72	0.86	0.24	0.85	1.11
11JY2672	郑29	505	NP	1	53.68	44.18	16.21	3.46	0.90	0.25	0.82	1.15
11JY2675	郑30	506	NP	1	60.02	41.18	16.96	3.46	0.98	0.23	0.69	1.21
11JY2677	郑35	507	NP	1	57.02	50.85	15.02	2.85	0.91	0.20	0.89	1.11
11JY2697	综31	508	NP	1	66.35	34.52	14.98	2.70	0.84	0.15	0.52	0.99
12JY0001	C5 RIL P2	509	NP	1	77.22	39.30	11.39	4.61	0.88	0.31	0.51	1.19
12JY0002	C5 RIL P1	510	NP	1	74.55	34.63	6.08	2.28	0.29	0.15	0.46	0.45
12JY0015	[(SML*SMQPM)*(MTL*SMQPM)]F1S6-1-25-BB-1-B	511	NP	1	93.22	33.97	13.06	3.87	0.63	0.22	0.36	0.85
12JY0017	[CML159/[CML159/[MSRXPOOL9]C1F2-205-1(OSU23i)-5-3-X-X-1-BB]F2-3sx]-8-1-1-BB-1-B	512	NP	1	82.22	54.63	11.60	4.64	0.65	0.29	0.66	0.94
12JY0018	[CML198/LPSC3H144-1-2-2-2-#-BB]-1-4-1-1-4-B*4-B-B-B	513	NP	1	80.22	57.30	14.00	5.23	0.83	0.36	0.71	1.20
12JY0029	[DTPWC8F31-4-2-1-6/CML444//ZM521B-66-4-1-1-1-BB]-3-2-1-B-B-B	514	NP	1	70.55	51.63	6.53	2.37	0.50	0.14	0.73	0.64
12JY0040	18-599	515	NP	1	92.55	27.63	8.36	1.10	0.80	0.15	0.30	0.96
12JY0041	18-599(RED)	516	NP	1	67.34	36.80	7.93	1.34	0.59	0.08	0.55	0.68
12JY0108	CML330	517	NP	1	68.55	55.30	7.49	2.61	0.55	0.17	0.81	0.71
12JY0122	CML418	518	NP	1	74.55	37.97	6.40	1.20	0.73	0.11	0.51	0.84
12JY0146	CML504	519	NP	1	73.88	52.30	6.77	1.59	0.67	0.14	0.71	0.82
12JY0156	Cuba/Guad C3 F53-3-1-1-B-B-B	520	NP	1	80.88	53.30	8.24	2.59	0.48	0.13	0.66	0.62
12JY0164	ECA-MOROSR(BC1)F2-7-ECAVEE7/PL15QPMC7SRC1F2//POOL15QPMSR-B-4-B-B	521	NP	1	79.55	33.63	9.91	3.32	0.60	0.19	0.42	0.79
12JY0167	INTA-191-2-1-2-B*8-B-B-B	522	NP	1	71.55	32.97	4.72	1.34	0.38	0.11	0.46	0.49
12JY0173	La Posta Seq C7-F86-1-1-1-1-B-B-B	523	NP	1	66.05	40.63	4.55	2.25	0.39	0.13	0.62	0.53
12JY0185	P501SRc0-F2-47-3-1-1-B-B-B-B	524	NP	1	63.55	38.63	2.50	1.20	0.37	0.09	0.61	0.46
12JY0208	VL05610	525	NP	1	77.05	20.63	3.10	1.21	0.36	0.09	0.27	0.45
12JY0226	178	526	NP	1	60.55	18.30	2.48	1.15	0.29	0.07	0.30	0.36
12JY0228	273	527	NP	1	66.23	42.76	8.91	2.03	0.56	0.15	0.65	0.71
12JY0229	288	528	NP	1	76.88	33.63	6.54	1.74	0.39	0.08	0.44	0.47
12JY0240	764	529	NP	1	64.36	56.80	11.18	3.12	0.85	0.20	0.88	1.05
12JY0246	6103	530	NP	1	84.22	60.63	12.95	3.99	0.91	0.21	0.72	1.12
12JY0252	81565	531	NP	1	67.02	38.63	8.08	2.31	0.56	0.12	0.58	0.68
12JY0264	634-11511	532	NP	1	69.36	47.30	10.03	1.91	0.82	0.15	0.68	0.97

Seed ID	Acc	Gen	trt.	rep	SL	RL	SFW	RFW	SDW	RDW	RSR	TDM
12JY0265	698-1	533	NP	1	70.22	39.63	9.11	1.73	0.65	0.10	0.56	0.75
12JY0308	FAPW	534	NP	1	91.55	43.80	16.09	3.03	1.05	0.24	0.48	1.28
12JY0322	LH132	535	NP	1	81.22	53.13	14.13	2.72	0.73	0.22	0.65	0.95
12JY0323	LH51	536	NP	1	77.55	32.80	11.74	3.27	0.65	0.26	0.42	0.90
12JY0325	LX9801	537	NP	1	76.88	39.13	8.10	1.84	0.50	0.16	0.51	0.65
12JY0329	Mo17	538	NP	1	78.55	25.13	13.01	2.64	0.51	0.17	0.32	0.68
12JY0347	R08	539	NP	1	78.88	40.80	6.68	2.07	0.42	0.14	0.52	0.56
12JY0349	RP125	540	NP	1	59.69	45.47	11.33	2.88	0.77	0.16	0.76	0.93
12JY0365	Zhao835	541	NP	1	81.55	29.80	14.95	3.45	0.63	0.22	0.37	0.84
12JY0370	昌7-2	542	NP	1	76.38	44.63	10.31	2.37	0.56	0.17	0.58	0.73
12JY0383	丹598	543	NP	1	81.88	34.47	11.69	2.87	0.78	0.22	0.42	1.00
12JY0401	黄早四	544	NP	1	73.88	40.97	10.72	2.63	0.69	0.17	0.55	0.87
12JY0416	吉853	545	NP	1	82.88	36.80	14.11	3.37	0.64	0.20	0.44	0.83
12JY0443	南21-3	546	NP	1	89.88	53.47	16.03	4.34	1.09	0.32	0.59	1.41
12JY0450	齐319	547	NP	1	72.23	44.34	11.47	3.07	0.69	0.23	0.61	0.92
12JY0457	沈5003	548	NP	1	84.55	54.63	16.81	4.40	1.05	0.36	0.65	1.40
12JY0462	四287	549	NP	1	88.30	53.80	20.50	5.63	1.33	0.32	0.61	1.65
12JY0473	铁7922	550	NP	1	83.55	32.80	17.81	4.77	0.96	0.28	0.39	1.24
11JY1255	C5 RIL 2	1	NP	2	49.99	37.80	9.46	4.01	0.62	0.23	0.76	0.85
11JY1256	C5 RIL 3	2	NP	2	47.33	36.80	7.06	2.24	0.55	0.14	0.78	0.68
11JY1258	C5 RIL 5	3	NP	2	57.66	41.13	14.62	4.54	1.16	0.28	0.71	1.44
11JY1259	C5 RIL 6	4	NP	2	53.99	29.13	9.00	2.83	0.52	0.15	0.54	0.67
11JY1262	C5 RIL 10	5	NP	2	49.49	35.13	8.76	4.04	0.64	0.26	0.71	0.90
11JY1263	C5 RIL 11	6	NP	2	55.66	33.47	14.05	4.18	1.09	0.26	0.60	1.35
11JY1264	C5 RIL 12	7	NP	2	43.33	43.80	5.16	2.05	0.38	0.14	1.01	0.52
11JY1267	C5 RIL 17	8	NP	2	52.33	40.80	11.23	4.54	0.72	0.22	0.78	0.94
11JY1268	C5 RIL 18	9	NP	2	49.99	37.13	10.30	3.42	0.84	0.21	0.74	1.04
11JY1269	C5 RIL 19	10	NP	2	51.49	34.63	12.20	4.87	0.87	0.27	0.67	1.13
11JY1271	C5 RIL 21	11	NP	2	48.99	36.47	8.78	3.61	0.61	0.22	0.74	0.82
11JY1272	C5 RIL 24	12	NP	2	44.66	33.80	6.83	3.22	0.46	0.19	0.76	0.65
11JY1274	C5 RIL 26	13	NP	2	54.33	30.80	11.70	2.91	0.69	0.14	0.57	0.83
11JY1275	C5 RIL 27	14	NP	2	41.49	42.63	8.22	3.27	0.57	0.15	1.03	0.72
11JY1276	C5 RIL 28	15	NP	2	44.66	24.13	5.84	1.68	0.38	0.10	0.54	0.48
11JY1278	C5 RIL 30	16	NP	2	40.99	34.47	5.78	2.49	0.39	0.14	0.84	0.53
11JY1279	C5 RIL 31	17	NP	2	39.66	31.13	8.44	2.63	0.64	0.16	0.78	0.80
11JY1280	C5 RIL 32	18	NP	2	61.66	34.13	18.07	5.94	1.38	0.41	0.55	1.79

Seed ID	Acc	Gen	trt.	rep	SL	RL	SFW	RFW	SDW	RDW	RSR	TDM
11JY1281	C5 RIL 34	19	NP	2	49.33	31.13	9.34	3.61	0.63	0.20	0.63	0.83
11JY1282	C5 RIL 36	20	NP	2	53.33	28.13	10.65	4.49	0.53	0.23	0.53	0.75
11JY1283	C5 RIL 38	21	NP	2	37.33	31.47	4.39	2.80	0.35	0.13	0.84	0.48
11JY1284	C5 RIL 39	22	NP	2	43.33	31.80	5.00	1.96	0.46	0.16	0.73	0.62
11JY1285	C5 RIL 40	23	NP	2	55.99	38.80	9.44	4.09	0.71	0.29	0.69	1.00
11JY1286	C5 RIL 41	24	NP	2	47.66	31.80	7.58	3.12	0.59	0.18	0.67	0.77
11JY1287	C5 RIL 42	25	NP	2	51.33	33.13	8.77	3.24	0.66	0.18	0.65	0.84
11JY1288	C5 RIL 44	26	NP	2	48.33	34.63	5.15	2.92	0.41	0.12	0.72	0.53
11JY1289	C5 RIL 45	27	NP	2	54.33	36.80	11.72	3.72	0.92	0.30	0.68	1.22
11JY1290	C5 RIL 46	28	NP	2	38.99	33.13	5.81	3.35	0.36	0.15	0.85	0.51
11JY1292	C5 RIL 48	29	NP	2	52.33	34.13	9.27	2.48	0.65	0.18	0.65	0.83
11JY1293	C5 RIL 49	30	NP	2	58.66	33.80	10.12	3.84	0.87	0.26	0.58	1.13
11JY1299	C5 RIL 57	31	NP	2	49.33	30.63	6.83	3.26	0.56	0.16	0.62	0.72
11JY1301	C5 RIL 59	32	NP	2	53.66	44.13	11.16	4.76	0.96	0.30	0.82	1.26
11JY1305	C5 RIL 64	33	NP	2	59.33	41.63	11.07	3.65	0.89	0.26	0.70	1.14
11JY1310	C5 RIL 68	34	NP	2	46.83	39.13	7.16	2.79	0.43	0.14	0.84	0.57
11JY1312	C5 RIL 70	35	NP	2	58.66	37.80	12.77	3.55	0.89	0.29	0.64	1.18
11JY1315	C5 RIL 82	36	NP	2	45.16	36.30	7.32	2.11	0.51	0.17	0.80	0.68
11JY1316	C5 RIL 74	37	NP	2	51.66	33.13	9.83	2.71	0.90	0.22	0.64	1.12
11JY1318	C5 RIL 78	38	NP	2	55.66	36.47	10.07	3.09	0.81	0.24	0.66	1.06
11JY1319	C5 RIL 79	39	NP	2	54.16	35.30	9.68	2.53	0.68	0.19	0.65	0.87
11JY1320	C5 RIL 80	40	NP	2	49.33	40.47	6.88	2.44	0.61	0.19	0.82	0.80
11JY1323	C5 RIL 84	41	NP	2	51.66	44.80	10.21	3.13	0.77	0.23	0.87	1.00
11JY1324	C5 RIL 86	42	NP	2	46.66	37.13	9.01	2.96	0.63	0.21	0.80	0.83
11JY1326	C5 RIL 88	43	NP	2	52.99	40.47	9.48	2.62	0.65	0.22	0.76	0.86
11JY1327	C5 RIL 89	44	NP	2	56.66	36.80	12.10	3.81	0.84	0.28	0.65	1.12
11JY1328	C5 RIL 90	45	NP	2	48.33	37.47	11.88	3.65	0.79	0.24	0.78	1.03
11JY1329	C5 RIL 92	46	NP	2	47.66	36.30	11.23	2.98	0.65	0.23	0.76	0.88
11JY1333	C5 RIL 96	47	NP	2	63.99	42.80	15.15	4.42	1.12	0.34	0.67	1.46
11JY1334	C5 RIL 99	48	NP	2	56.99	45.47	12.33	4.53	0.99	0.34	0.80	1.33
11JY1336	C5 RIL 102	49	NP	2	46.99	32.47	7.47	2.00	0.68	0.19	0.69	0.88
11JY1337	C5 RIL 103	50	NP	2	44.66	33.80	7.68	2.55	0.64	0.17	0.76	0.82
11JY1339	C5 RIL 106	51	NP	2	60.33	33.13	15.23	4.88	1.20	0.35	0.55	1.55
11JY1341	C5 RIL 108	52	NP	2	42.83	36.80	5.07	2.82	0.38	0.19	0.86	0.57
11JY1349	C5 RIL 119	53	NP	2	53.33	44.63	7.77	3.44	0.70	0.24	0.84	0.93
11JY1350	C5 RIL 120	54	NP	2	49.99	39.30	8.47	3.76	0.61	0.18	0.79	0.79

Seed ID	Acc	Gen	trt.	rep	SL	RL	SFW	RFW	SDW	RDW	RSR	TDM
11JY1351	C5 RIL 121	55	NP	2	57.83	45.80	12.62	4.48	0.89	0.26	0.79	1.15
11JY1352	C5 RIL 122	56	NP	2	46.66	43.97	8.59	2.68	0.63	0.15	0.94	0.77
11JY1354	C5 RIL 124	57	NP	2	56.99	36.30	11.26	3.00	0.92	0.27	0.64	1.19
11JY1356	C5 RIL 126	58	NP	2	51.66	33.30	7.98	2.26	0.61	0.15	0.64	0.76
11JY1357	C5 RIL 128	59	NP	2	51.66	25.30	9.17	2.69	0.70	0.17	0.49	0.87
11JY1360	C5 RIL 131	60	NP	2	45.99	39.30	9.07	4.37	0.69	0.25	0.85	0.94
11JY1362	C5 RIL 133	61	NP	2	48.33	42.63	9.60	2.49	0.60	0.15	0.88	0.75
11JY1363	C5 RIL 134	62	NP	2	46.99	51.97	7.63	2.67	0.59	0.19	1.11	0.77
11JY1364	C5 RIL 135	63	NP	2	56.66	49.97	14.55	5.62	1.16	0.37	0.88	1.54
11JY1365	C5 RIL 137	64	NP	2	51.33	48.30	8.48	3.24	0.57	0.14	0.94	0.71
11JY1366	C5 RIL 138	65	NP	2	45.66	44.63	8.55	2.99	0.61	0.15	0.98	0.76
11JY1367	C5 RIL 139	66	NP	2	45.33	32.80	4.08	1.75	0.26	0.08	0.72	0.34
11JY1368	C5 RIL 140	67	NP	2	52.33	37.97	9.76	3.28	0.78	0.18	0.73	0.96
11JY1369	C5 RIL 141	68	NP	2	53.33	47.63	14.11	4.48	1.07	0.24	0.89	1.31
11JY1370	C5 RIL 142	69	NP	2	36.83	30.80	5.03	1.47	0.29	0.05	0.84	0.34
11JY1372	C5 RIL 144	70	NP	2	57.66	57.97	12.67	4.35	1.10	0.33	1.01	1.43
11JY1374	C5 RIL 146	71	NP	2	49.16	41.13	9.97	2.53	0.59	0.15	0.84	0.73
11JY1376	C5 RIL 150	72	NP	2	57.83	44.80	11.36	3.31	0.79	0.19	0.77	0.97
11JY1377	C5 RIL 151	73	NP	2	58.50	40.60	12.78	3.38	1.00	0.35	0.69	1.35
11JY1378	C5 RIL 152	74	NP	2	41.20	30.00	4.64	1.25	0.36	0.13	0.73	0.49
11JY1379	C5 RIL 153	75	NP	2	50.15	26.25	6.71	2.00	0.55	0.14	0.52	0.69
11JY1382	C5 RIL 156	76	NP	2	56.10	26.00	7.80	1.22	0.73	0.18	0.46	0.91
11JY1383	C5 RIL 157	77	NP	2	34.80	25.40	4.55	1.30	0.36	0.12	0.73	0.48
11JY1384	C5 RIL 158	78	NP	2	53.03	51.73	9.24	2.65	0.77	0.22	0.98	0.99
11JY1385	C5 RIL 159	79	NP	2	50.25	36.95	8.00	2.97	0.50	0.15	0.74	0.65
11JY1387	C5 RIL 161	80	NP	2	55.97	33.60	7.88	1.98	0.58	0.13	0.60	0.71
11JY1388	C5 RIL 162	81	NP	2	54.50	35.30	4.85	1.33	0.40	0.10	0.65	0.50
11JY1389	C5 RIL 163	82	NP	2	50.00	27.75	6.38	1.56	0.48	0.15	0.56	0.63
11JY1391	C5 RIL 165	83	NP	2	52.30	33.20	8.53	1.25	0.62	0.21	0.63	0.83
11JY1394	C5 RIL 169	84	NP	2	33.60	31.80	2.72	0.60	0.21	0.10	0.95	0.31
11JY1396	C5 RIL 171	85	NP	2	45.15	23.05	5.05	1.04	0.38	0.40	0.51	0.78
11JY1397	C5 RIL 172	86	NP	2	51.57	30.67	6.95	1.80	0.57	0.18	0.59	0.75
11JY1399	C5 RIL 174	87	NP	2	43.00	24.00	7.47	2.29	0.61	0.19	0.56	0.80
11JY1400	C5 RIL 176	88	NP	2	52.17	43.60	10.48	3.13	0.84	0.26	0.84	1.10
11JY1401	C5 RIL 177	89	NP	2	48.27	44.03	8.20	2.44	0.64	0.16	0.91	0.80
11JY1402	C5 RIL 178	90	NP	2	46.35	28.50	6.11	2.25	0.49	0.16	0.61	0.65

Seed ID	Acc	Gen	trt.	rep	SL	RL	SFW	RFW	SDW	RDW	RSR	TDM
11JY1404	C5 RIL 182	91	NP	2	39.30	28.50	6.55	2.45	0.55	0.20	0.73	0.75
11JY1405	C5 RIL 183	92	NP	2	46.07	30.20	8.56	2.38	0.74	0.21	0.66	0.95
11JY1406	C5 RIL 184	93	NP	2	41.00	40.00	6.72	2.82	0.51	0.22	0.98	0.73
11JY1409	C5 RIL 187	94	NP	2	42.60	32.90	5.77	2.30	0.46	0.17	0.77	0.63
11JY1410	C5 RIL 188	95	NP	2	36.20	33.50	5.98	1.54	0.39	0.14	0.93	0.53
11JY1412	C5 RIL 191	96	NP	2	51.00	47.10	11.01	3.12	0.98	0.30	0.92	1.28
11JY1413	C5 RIL 192	97	NP	2	41.85	36.95	5.58	1.96	0.49	0.19	0.88	0.68
11JY1414	C5 RIL 193	98	NP	2	32.00	52.40	4.38	1.51	0.35	0.20	1.64	0.55
11JY1416	C5 RIL 195	99	NP	2	44.20	28.30	6.58	1.02	0.66	0.25	0.64	0.91
11JY1418	C5 RIL 198	100	NP	2	41.90	42.90	6.41	2.03	0.55	0.25	1.02	0.80
11JY1420	C5 RIL 200	101	NP	2	34.00	27.20	8.75	2.46	0.70	0.30	0.80	1.00
11JY1421	C5 RIL 203	102	NP	2	32.00	29.40	5.50	1.11	0.37	0.17	0.92	0.54
11JY1422	C5 RIL 204	103	NP	2	32.00	34.40	5.50	1.11	0.47	0.16	1.08	0.63
11JY1423	C5 RIL 205	104	NP	2	42.70	34.30	6.08	1.10	0.49	0.15	0.80	0.64
11JY1424	C5 RIL 206	105	NP	2	53.23	38.80	9.46	3.26	0.76	0.27	0.73	1.03
11JY1425	C5 RIL 207	106	NP	2	34.30	31.80	4.04	2.15	0.29	0.14	0.93	0.43
11JY1426	C5 RIL 208	107	NP	2	33.40	32.40	9.18	4.96	0.54	0.29	0.97	0.83
11JY1428	C5 RIL 210	108	NP	2	43.95	39.00	11.45	4.83	0.85	0.30	0.89	1.15
11JY1429	C5 RIL 211	109	NP	2	40.33	32.80	2.57	1.69	0.49	0.11	0.81	0.60
11JY1429	C5 RIL 211	110	NP	2	24.50	27.65	11.51	1.23	0.18	0.11	1.13	0.29
11JY1431	C5 RIL 214	111	NP	2	36.70	36.70	7.90	3.84	0.59	0.22	1.00	0.81
11JY1433	C5 RIL 216	112	NP	2	23.60	27.10	3.62	1.99	0.31	0.15	1.15	0.46
11JY1434	C5 RIL 217	113	NP	2	26.50	19.50	2.98	1.01	0.23	0.10	0.74	0.33
11JY1435	C5 RIL 218	114	NP	2	35.55	30.90	5.81	2.34	0.45	0.16	0.87	0.61
11JY1437	C5 RIL 220	115	NP	2	54.80	32.70	10.88	3.82	0.78	0.22	0.60	1.00
11JY1440	C5 RIL 225	116	NP	2	30.60	31.40	3.63	1.53	0.22	0.10	1.03	0.32
11JY1442	C5 RIL 227	117	NP	2	35.70	29.23	8.25	3.96	0.56	0.25	0.82	0.81
11JY1444	C5 RIL 229	118	NP	2	39.50	43.80	11.81	3.83	0.72	0.27	1.11	0.99
11JY1454	C5 RIL 240	119	NP	2	35.25	34.40	8.13	2.58	0.56	0.18	0.98	0.74
11JY1455	C5 RIL 241	120	NP	2	39.55	28.00	5.47	1.61	0.39	0.18	0.71	0.57
11JY1456	C5 RIL 243	121	NP	2	50.45	40.45	13.09	3.70	0.86	0.26	0.80	1.12
11JY1459	C5 RIL P2	122	NP	2	40.50	37.20	8.28	3.47	0.66	0.27	0.92	0.93
11JY1460	C5 RIL P1	123	NP	2	35.70	32.00	3.91	3.00	0.53	0.21	0.90	0.74
11JY1461	C6 RIL 3	124	NP	2	41.90	24.30	3.84	0.89	0.31	0.14	0.58	0.45
11JY1462	C6 RIL 14	125	NP	2	54.43	45.70	6.62	3.48	0.49	0.14	0.84	0.63
11JY1463	C6 RIL 23	126	NP	2	66.67	53.33	12.89	5.05	1.06	0.35	0.80	1.41

Seed ID	Acc	Gen	trt.	rep	SL	RL	SFW	RFW	SDW	RDW	RSR	TDM
11JY1464	C6 RIL 24	127	NP	2	43.67	36.08	7.81	3.30	0.96	0.21	0.83	1.17
11JY1465	C6 RIL 25	128	NP	2	67.27	51.30	21.12	8.72	1.30	0.44	0.76	1.74
11JY1466	C6 RIL 29	129	NP	2	50.30	40.47	13.03	4.68	0.91	0.29	0.80	1.20
11JY1468	C6 RIL 144	130	NP	2	80.50	43.40	26.44	8.23	2.15	0.63	0.54	2.78
11JY1469	C6 RIL 203	131	NP	2	64.87	32.47	15.95	6.01	1.12	0.39	0.50	1.51
11JY1470	C6 RIL 212	132	NP	2	61.00	35.80	15.68	4.22	1.51	0.46	0.59	1.97
11JY1472	C6 RIL 219	133	NP	2	55.60	47.80	10.95	3.43	0.74	0.24	0.86	0.98
11JY1473	C6 RIL 239	134	NP	2	61.30	48.47	16.20	5.55	1.21	0.43	0.79	1.64
11JY1474	C6 RIL 243	135	NP	2	58.80	45.00	12.54	4.43	0.97	0.37	0.77	1.34
11JY1475	C6 RIL 272	136	NP	2	55.83	49.93	8.81	2.78	0.58	0.12	0.89	0.70
11JY1476	C6 RIL 273	137	NP	2	51.00	44.30	12.93	4.46	1.07	0.46	0.87	1.53
11JY1477	C6 RIL 283	138	NP	2	65.95	38.45	15.40	4.99	1.35	0.41	0.58	1.76
11JY1478	C6 RIL 293	139	NP	2	75.37	40.07	21.15	4.93	1.56	0.48	0.53	2.04
11JY1479	C6 RIL 298	140	NP	2	44.50	41.70	7.28	1.40	0.96	0.18	0.94	1.14
11JY1480	C6 RIL 303	141	NP	2	55.53	40.50	15.09	3.79	1.02	0.35	0.73	1.37
11JY1481	C6 RIL 327	142	NP	2	40.00	31.00	6.00	3.10	0.49	0.24	0.78	0.73
11JY1482	C6 RIL 341	143	NP	2	42.00	39.00	8.71	4.32	0.68	0.31	0.93	0.99
11JY1483	C6 RIL 348	144	NP	2	17.00	26.00	1.30	0.92	0.15	0.08	1.53	0.23
11JY1484	C6 RIL 402	145	NP	2	47.00	42.00	10.16	5.81	0.87	0.45	0.89	1.32
11JY1485	C6 RIL 403	146	NP	2	34.50	30.00	3.47	1.00	0.30	0.11	0.87	0.41
11JY1487	C6 RIL 5	147	NP	2	55.23	37.90	6.01	2.25	0.44	0.12	0.69	0.56
11JY1488	C6 RIL 7	148	NP	2	35.00	37.00	6.03	2.86	0.57	0.35	1.06	0.92
11JY1489	C6 RIL 10	149	NP	2	53.60	36.55	10.85	3.76	0.75	0.27	0.68	1.02
11JY1490	C6 RIL 15	150	NP	2	58.63	43.70	15.23	6.08	1.12	0.45	0.75	1.57
11JY1491	C6 RIL 19	151	NP	2	56.93	41.13	10.67	3.55	0.78	0.27	0.72	1.05
11JY1493	C6 RIL 21	152	NP	2	55.80	43.45	11.79	3.04	0.89	0.30	0.78	1.19
11JY1494	C6 RIL 23	153	NP	2	59.20	50.00	9.35	2.74	0.78	0.29	0.84	1.07
11JY1495	C6 RIL 24	154	NP	2	55.70	47.37	12.48	4.89	0.98	0.52	0.85	1.50
11JY1496	C6 RIL 25	155	NP	2	55.43	45.87	11.61	3.40	0.91	0.37	0.83	1.28
11JY1497	C6 RIL 28	156	NP	2	33.60	38.90	3.47	0.70	0.30	0.15	1.16	0.45
11JY1498	C6 RIL 29	157	NP	2	43.37	37.57	10.92	5.49	0.84	0.40	0.87	1.24
11JY1499	C6 RIL 30	158	NP	2	57.25	41.90	6.78	3.38	0.52	0.20	0.73	0.72
11JY1500	C6 RIL 31	159	NP	2	49.05	39.25	10.85	3.59	0.90	0.34	0.80	1.24
11JY1501	C6 RIL 32	160	NP	2	72.70	43.50	22.53	7.05	1.66	0.57	0.60	2.23
11JY1502	C6 RIL 33	161	NP	2	38.40	33.80	6.37	2.09	0.44	0.13	0.88	0.57
11JY1503	C6 RIL 36	162	NP	2	56.23	39.20	12.64	5.16	1.07	0.37	0.70	1.44

Seed ID	Acc	Gen	trt.	rep	SL	RL	SFW	RFW	SDW	RDW	RSR	TDM
11JY1504	C6 RIL 39	163	NP	2	56.97	44.87	15.14	5.76	0.94	0.35	0.79	1.29
11JY1505	C6 RIL 40	164	NP	2	55.63	49.33	12.88	3.20	0.86	0.29	0.89	1.15
11JY1506	C6 RIL 42	165	NP	2	59.93	36.63	15.54	3.76	1.44	0.43	0.61	1.87
11JY1508	C6 RIL 47	166	NP	2	73.00	57.45	22.63	5.77	1.89	0.64	0.79	2.53
11JY1509	C6 RIL 49	167	NP	2	41.50	29.70	13.52	1.45	0.27	0.12	0.72	0.39
11JY1510	C6 RIL 50	168	NP	2	57.93	53.97	16.19	4.16	1.27	0.44	0.93	1.71
11JY1511	C6 RIL 52	169	NP	2	51.50	36.00	6.82	3.64	0.48	0.18	0.70	0.66
11JY1512	C6 RIL 53	170	NP	2	56.57	49.70	13.85	2.69	0.93	0.37	0.88	1.30
11JY1513	C6 RIL 57	171	NP	2	42.30	36.20	6.52	1.25	0.45	0.16	0.86	0.61
11JY1514	C6 RIL 58	172	NP	2	57.20	38.50	11.49	2.30	0.72	0.21	0.67	0.93
11JY1515	C6 RIL 59	173	NP	2	43.25	35.65	6.02	0.99	0.41	0.13	0.82	0.54
11JY1516	C6 RIL 60	174	NP	2	45.95	39.20	7.99	2.95	0.59	0.26	0.85	0.85
11JY1517	C6 RIL 64	175	NP	2	58.87	32.20	8.10	1.93	0.67	0.16	0.55	0.83
11JY1518	C6 RIL 65	176	NP	2	66.13	51.87	15.18	3.97	0.98	0.25	0.78	1.23
11JY1519	C6 RIL 66	177	NP	2	57.70	51.00	13.13	4.68	0.90	0.26	0.88	1.16
11JY1520	C6 RIL 68	178	NP	2	52.30	41.80	8.25	2.00	0.60	0.17	0.80	0.77
11JY1521	C6 RIL 69	179	NP	2	47.30	51.90	9.83	3.33	0.65	0.22	1.10	0.87
11JY1522	C6 RIL 70	180	NP	2	50.61	48.40	6.91	1.76	0.40	0.12	0.96	0.52
11JY1523	C6 RIL 72	181	NP	2	65.70	55.60	18.04	6.07	1.11	0.38	0.85	1.49
11JY1524	C6 RIL 74	182	NP	2	50.87	47.10	12.26	4.24	0.70	0.22	0.93	0.92
11JY1525	C6 RIL 76	183	NP	2	46.70	44.40	10.11	3.49	0.60	0.25	0.95	0.85
11JY1527	C6 RIL 79	184	NP	2	40.93	33.20	6.62	1.70	0.54	0.21	0.81	0.75
11JY1528	C6 RIL 83	185	NP	2	65.20	35.17	16.59	5.08	1.33	0.37	0.54	1.70
11JY1529	C6 RIL 85	186	NP	2	56.80	46.70	12.22	2.72	0.86	0.34	0.82	1.20
11JY1530	C6 RIL 86	187	NP	2	60.45	38.25	9.97	1.42	0.65	0.18	0.63	0.83
11JY1531	C6 RIL 91	188	NP	2	61.53	37.37	18.02	4.65	1.42	0.42	0.61	1.84
11JY1532	C6 RIL 94	189	NP	2	44.63	39.13	6.18	1.44	0.44	0.13	0.88	0.57
11JY1533	C6 RIL 95	190	NP	2	54.30	51.63	9.93	3.97	0.72	0.26	0.95	0.98
11JY1534	C6 RIL 97	191	NP	2	50.23	49.03	6.74	2.58	0.42	0.13	0.98	0.55
11JY1535	C6 RIL 103	192	NP	2	29.60	43.30	4.10	2.46	0.26	0.11	1.46	0.37
11JY1536	C6 RIL 104	193	NP	2	43.73	33.93	4.23	1.41	0.28	0.10	0.78	0.38
11JY1537	C6 RIL 107	194	NP	2	34.50	24.70	3.03	0.61	0.21	0.07	0.72	0.28
11JY1538	C6 RIL 108	195	NP	2	56.17	38.65	11.07	3.02	0.72	0.22	0.69	0.94
11JY1539	C6 RIL 109	196	NP	2	42.50	38.07	3.68	1.49	0.23	0.09	0.90	0.32
11JY1540	C6 RIL 112	197	NP	2	53.53	39.13	8.83	3.03	0.55	0.16	0.73	0.71
11JY1541	C6 RIL 113	198	NP	2	54.83	41.00	13.25	3.09	0.98	0.30	0.75	1.28

Seed ID	Acc	Gen	trt.	rep	SL	RL	SFW	RFW	SDW	RDW	RSR	TDM
11JY1542	C6 RIL 115	199	NP	2	67.10	35.60	13.19	1.89	1.11	0.24	0.53	1.35
11JY1543	C6 RIL 116	200	NP	2	44.60	40.65	4.10	0.42	0.32	0.11	0.91	0.43
11JY1544	C6 RIL 117	201	NP	2	32.66	43.00	10.41	1.43	0.75	0.27	1.32	1.02
11JY1545	C6 RIL 120	202	NP	2	62.77	49.30	9.00	3.10	0.62	0.17	0.79	0.79
11JY1546	C6 RIL 121	203	NP	2	61.00	33.15	14.21	2.05	1.15	0.35	0.54	1.50
11JY1547	C6 RIL 123	204	NP	2	67.80	34.60	15.90	2.71	1.35	0.43	0.51	1.78
11JY1548	C6 RIL 124	205	NP	2	50.83	40.05	9.84	1.30	0.76	0.25	0.79	1.01
11JY1549	C6 RIL 127	206	NP	2	47.80	32.13	7.77	1.99	0.60	0.16	0.67	0.76
11JY1550	C6 RIL 131	207	NP	2	63.85	31.85	13.18	3.11	1.07	0.22	0.50	1.29
11JY1551	C6 RIL 134	208	NP	2	62.45	32.70	11.33	5.35	1.67	0.34	0.52	2.01
11JY1552	C6 RIL 135	209	NP	2	61.97	39.70	14.72	4.42	1.11	0.29	0.64	1.40
11JY1553	C6 RIL 138	210	NP	2	63.20	42.77	13.85	4.37	1.05	0.33	0.68	1.38
11JY1554	C6 RIL 139	211	NP	2	36.70	28.90	2.50	0.60	0.20	0.08	0.79	0.28
11JY1555	C6 RIL 140	212	NP	2	59.10	37.90	9.65	1.58	1.19	0.20	0.64	1.39
11JY1556	C6 RIL 141	213	NP	2	46.93	35.03	5.03	1.34	0.35	0.09	0.75	0.44
11JY1557	C6 RIL 142	214	NP	2	79.55	39.60	25.80	6.96	1.82	0.61	0.50	2.43
11JY1558	C6 RIL 143	215	NP	2	62.35	44.55	14.41	3.57	1.05	0.31	0.71	1.36
11JY1560	C6 RIL 146	216	NP	2	53.15	43.70	8.52	1.79	0.61	0.16	0.82	0.77
11JY1561	C6 RIL 147	217	NP	2	28.60	27.50	2.30	0.36	0.19	0.10	0.96	0.29
11JY1562	C6 RIL 148	218	NP	2	44.77	42.05	4.88	0.87	0.41	0.16	0.94	0.57
11JY1563	C6 RIL 150	219	NP	2	44.40	41.20	5.56	0.84	0.49	0.14	0.93	0.63
11JY1565	C6 RIL 153	220	NP	2	43.50	42.70	9.78	1.92	0.73	0.28	0.98	1.01
11JY1566	C6 RIL 156	221	NP	2	39.90	38.80	3.47	0.49	0.46	0.17	0.97	0.63
11JY1567	C6 RIL 160	222	NP	2	63.67	37.40	11.28	3.81	0.87	0.25	0.59	1.12
11JY1568	C6 RIL 161	223	NP	2	53.55	36.15	8.38	2.95	0.59	0.17	0.68	0.76
11JY1569	C6 RIL 162	224	NP	2	62.00	35.55	9.73	2.57	0.74	0.16	0.57	0.90
11JY1570	C6 RIL 163	225	NP	2	48.20	32.93	7.85	2.28	0.53	0.14	0.68	0.67
11JY1571	C6 RIL 164	226	NP	2	59.15	37.40	9.12	2.62	0.43	0.17	0.63	0.60
11JY1572	C6 RIL 169	227	NP	2	58.30	28.37	7.78	1.21	0.61	0.15	0.49	0.76
11JY1573	C6 RIL 171	228	NP	2	58.43	31.40	8.59	1.44	0.69	0.17	0.54	0.86
11JY1574	C6 RIL 172	229	NP	2	69.25	52.10	15.82	4.97	1.14	0.42	0.75	1.56
11JY1576	C6 RIL 174	230	NP	2	37.70	32.40	1.87	0.48	0.16	0.08	0.86	0.24
11JY1577	C6 RIL 175	231	NP	2	28.10	26.30	1.80	0.65	0.17	0.10	0.94	0.27
11JY1578	C6 RIL 176	232	NP	2	46.93	31.13	5.91	1.37	0.55	0.18	0.66	0.73
11JY1579	C6 RIL 179	233	NP	2	31.80	31.60	2.56	0.23	0.26	0.08	0.99	0.34
11JY1580	C6 RIL 181	234	NP	2	38.80	30.40	3.85	0.54	0.40	0.16	0.78	0.56

Seed ID	Acc	Gen	trt.	rep	SL	RL	SFW	RFW	SDW	RDW	RSR	TDM
11JY1581	C6 RIL 184	235	NP	2	54.60	32.80	5.13	0.94	0.49	0.21	0.60	0.70
11JY1582	C6 RIL 186	236	NP	2	57.47	38.07	10.62	5.34	0.85	0.26	0.66	1.11
11JY1583	C6 RIL 187	237	NP	2	40.80	33.80	3.45	0.39	0.33	0.13	0.83	0.46
11JY1584	C6 RIL 188	238	NP	2	52.07	53.53	7.13	1.23	0.61	0.18	1.03	0.79
11JY1585	C6 RIL 189	239	NP	2	53.40	37.60	9.51	1.86	0.75	0.25	0.70	1.00
11JY1586	C6 RIL 190	240	NP	2	60.47	35.73	8.03	1.48	0.57	0.13	0.59	0.70
11JY1587	C6 RIL 192	241	NP	2	50.00	43.60	7.10	2.57	0.25	0.15	0.87	0.40
11JY1588	C6 RIL 193	242	NP	2	63.90	51.10	7.94	2.54	0.53	0.15	0.80	0.68
11JY1589	C6 RIL 203	243	NP	2	61.23	46.43	8.28	1.49	0.56	0.12	0.76	0.68
11JY1590	C6 RIL 204	244	NP	2	50.00	31.95	7.09	1.88	0.45	0.15	0.64	0.60
11JY1591	C6 RIL 205	245	NP	2	52.15	44.05	5.06	1.35	0.31	0.11	0.84	0.42
11JY1592	C6 RIL 206	246	NP	2	47.80	50.50	3.39	0.85	0.30	0.10	1.06	0.40
11JY1593	C6 RIL 207	247	NP	2	54.10	30.30	9.09	3.08	0.70	0.21	0.56	0.91
11JY1594	C6 RIL 211	248	NP	2	51.00	20.35	4.44	0.45	0.33	0.10	0.40	0.43
11JY1595	C6 RIL 212	249	NP	2	58.13	39.97	9.24	1.60	0.63	0.16	0.69	0.79
11JY1596	C6 RIL 217	250	NP	2	40.60	28.90	3.59	0.35	0.30	0.11	0.71	0.41
11JY1597	C6 RIL 218	251	NP	2	54.00	36.80	8.23	1.76	0.59	0.16	0.68	0.75
11JY1598	C6 RIL 219	252	NP	2	67.35	42.75	11.19	2.43	0.81	0.18	0.63	0.99
11JY1599	C6 RIL 220	253	NP	2	46.30	28.30	4.47	0.56	0.45	0.19	0.61	0.64
11JY1600	C6 RIL 225	254	NP	2	59.70	31.57	11.55	3.01	0.75	0.24	0.53	0.99
11JY1601	C6 RIL 230	255	NP	2	52.75	56.05	7.83	2.97	0.57	0.21	1.06	0.78
11JY1602	C6 RIL 231	256	NP	2	52.75	51.05	7.83	2.97	0.79	0.30	0.97	1.09
11JY1603	C6 RIL 232	257	NP	2	57.20	47.60	11.57	5.02	0.83	0.32	0.83	1.15
11JY1604	C6 RIL 234	258	NP	2	71.53	48.67	12.34	3.43	0.80	0.22	0.68	1.02
11JY1605	C6 RIL 235	259	NP	2	64.37	44.50	13.60	5.12	0.93	0.26	0.69	1.19
11JY1606	C6 RIL 236	260	NP	2	56.07	35.77	11.10	3.38	0.71	0.20	0.64	0.91
11JY1607	C6 RIL 238	261	NP	2	61.97	41.30	13.78	4.62	0.88	0.26	0.67	1.14
11JY1608	C6 RIL 239	262	NP	2	26.40	25.70	1.00	0.34	0.45	0.11	0.97	0.56
11JY1609	C6 RIL 241	263	NP	2	61.43	43.00	10.73	4.39	0.85	0.21	0.70	1.06
11JY1610	C6 RIL 243	264	NP	2	62.50	55.85	9.85	3.20	0.70	0.18	0.89	0.88
11JY1611	C6 RIL 245	265	NP	2	62.50	55.85	9.85	3.20	0.70	0.18	0.89	0.88
11JY1612	C6 RIL 249	266	NP	2	43.37	35.73	3.08	2.06	0.42	0.15	0.82	0.57
11JY1613	C6 RIL 253	267	NP	2	65.47	49.57	15.24	3.78	1.01	0.24	0.76	1.25
11JY1614	C6 RIL 258	268	NP	2	59.00	41.10	8.83	3.01	0.70	0.20	0.70	0.90
11JY1616	C6 RIL 261	269	NP	2	34.20	39.80	2.34	0.59	0.19	0.09	1.16	0.28
11JY1617	C6 RIL 262	270	NP	2	46.90	29.85	4.72	1.31	0.32	0.13	0.64	0.45

Seed ID	Acc	Gen	trt.	rep	SL	RL	SFW	RFW	SDW	RDW	RSR	TDM
11JY1619	C6 RIL 265	271	NP	2	61.77	51.17	9.28	2.64	0.69	0.15	0.83	0.84
11JY1620	C6 RIL 267	272	NP	2	58.00	51.03	9.49	2.58	0.60	0.16	0.88	0.76
11JY1621	C6 RIL 268	273	NP	2	47.77	47.73	0.75	3.69	0.30	0.09	1.00	0.39
11JY1622	C6 RIL 270	274	NP	2	59.60	42.33	10.23	3.91	0.71	0.23	0.71	0.94
11JY1623	C6 RIL 272	275	NP	2	61.80	44.57	9.72	4.61	0.74	0.26	0.72	1.00
11JY1624	C6 RIL 273	276	NP	2	48.83	37.27	4.38	1.84	0.46	0.13	0.76	0.59
11JY1625	C6 RIL 275	277	NP	2	53.57	30.70	9.05	3.65	0.73	0.21	0.57	0.94
11JY1626	C6 RIL 276	278	NP	2	48.17	35.90	6.64	2.82	0.48	0.18	0.75	0.66
11JY1627	C6 RIL 282	279	NP	2	66.67	39.97	17.68	6.14	1.71	0.50	0.60	2.21
11JY1628	C6 RIL 283	280	NP	2	72.37	39.17	15.19	7.91	1.35	0.56	0.54	1.91
11JY1629	C6 RIL 284	281	NP	2	65.75	36.70	14.86	7.23	1.13	0.41	0.56	1.54
11JY1630	C6 RIL 286	282	NP	2	67.40	50.15	18.46	8.26	1.54	0.53	0.74	2.07
11JY1631	C6 RIL 287	283	NP	2	61.80	53.60	14.20	5.25	1.33	0.36	0.87	1.69
11JY1632	C6 RIL 288	284	NP	2	34.70	33.20	1.75	0.54	0.16	0.06	0.96	0.22
11JY1633	C6 RIL 289	285	NP	2	68.00	33.60	11.65	4.87	1.02	0.29	0.49	1.31
11JY1634	C6 RIL 291	286	NP	2	67.60	58.90	16.24	7.32	1.39	0.46	0.87	1.85
11JY1635	C6 RIL 292	287	NP	2	41.27	44.93	5.98	2.40	0.53	0.20	1.09	0.73
11JY1636	C6 RIL 293	288	NP	2	72.53	50.03	16.77	8.12	1.36	0.51	0.69	1.87
11JY1637	C6 RIL 295	289	NP	2	66.03	57.75	14.72	4.93	1.26	0.40	0.87	1.66
11JY1638	C6 RIL 297	290	NP	2	64.47	34.63	14.28	3.78	1.18	0.28	0.54	1.46
11JY1639	C6 RIL 298	291	NP	2	55.60	44.07	8.93	3.05	0.79	0.18	0.79	0.97
11JY1640	C6 RIL 300	292	NP	2	58.37	34.30	15.17	4.55	1.15	0.38	0.59	1.53
11JY1641	C6 RIL 303	293	NP	2	60.33	45.67	10.70	3.54	1.01	0.29	0.76	1.30
11JY1642	C6 RIL 310	294	NP	2	67.00	58.00	12.55	6.57	1.07	0.46	0.87	1.53
11JY1643	C6 RIL 314	295	NP	2	37.60	25.80	1.88	0.48	0.19	0.07	0.69	0.26
11JY1644	C6 RIL 315	296	NP	2	74.17	36.67	17.16	5.74	1.13	0.34	0.49	1.47
11JY1645	C6 RIL 316	297	NP	2	66.50	59.00	16.56	5.78	1.08	0.38	0.89	1.46
11JY1646	C6 RIL 317	298	NP	2	66.00	47.00	16.41	6.58	1.22	0.44	0.71	1.66
11JY1647	C6 RIL 318	299	NP	2	52.50	44.50	8.42	3.03	0.67	0.26	0.85	0.93
11JY1648	C6 RIL 319	300	NP	2	46.00	47.00	5.54	1.72	0.42	0.18	1.02	0.60
11JY1649	C6 RIL 320	301	NP	2	64.07	40.33	13.53	5.70	1.00	0.35	0.63	1.35
11JY1650	C6 RIL 321	302	NP	2	37.75	36.50	4.26	0.98	0.28	0.11	0.97	0.39
11JY1651	C6 RIL 322	303	NP	2	63.33	50.17	16.41	4.96	1.21	0.44	0.79	1.65
11JY1652	C6 RIL 324	304	NP	2	63.70	52.53	11.10	4.99	0.92	0.40	0.82	1.32
11JY1653	C6 RIL 327	305	NP	2	72.90	48.37	15.74	4.56	1.22	0.38	0.66	1.60
11JY1654	C6 RIL 328	306	NP	2	59.50	29.10	5.28	1.77	0.31	0.12	0.49	0.43

Seed ID	Acc	Gen	trt.	rep	SL	RL	SFW	RFW	SDW	RDW	RSR	TDM
11JY1655	C6 RIL 330	307	NP	2	73.53	48.50	15.58	6.81	0.94	0.35	0.66	1.29
11JY1656	C6 RIL 332	308	NP	2	79.97	58.63	16.90	5.69	1.30	0.41	0.73	1.71
11JY1657	C6 RIL 333	309	NP	2	68.63	62.43	13.80	6.26	1.13	0.43	0.91	1.56
11JY1658	C6 RIL 335	310	NP	2	46.00	46.50	8.61	4.26	0.80	0.26	1.01	1.06
11JY1659	C6 RIL 336	311	NP	2	32.00	33.50	5.33	3.12	0.45	0.23	1.05	0.68
11JY1660	C6 RIL 339	312	NP	2	39.00	36.67	5.81	2.76	0.45	0.20	0.94	0.65
11JY1661	C6 RIL 340	313	NP	2	46.73	41.33	6.59	3.26	0.57	0.22	0.88	0.79
11JY1662	C6 RIL 341	314	NP	2	45.67	48.00	8.09	4.19	0.60	0.26	1.05	0.86
11JY1663	C6 RIL 342	315	NP	2	49.50	51.67	9.86	3.63	0.78	0.28	1.04	1.06
11JY1664	C6 RIL 344	316	NP	2	43.50	35.67	6.65	2.54	0.56	0.20	0.82	0.76
11JY1665	C6 RIL 346	317	NP	2	53.97	30.43	5.60	2.04	0.48	0.16	0.56	0.64
11JY1666	C6 RIL 347	318	NP	2	59.33	55.33	10.29	3.49	1.07	0.33	0.93	1.40
11JY1667	C6 RIL 349	319	NP	2	61.50	53.50	11.09	4.35	0.91	0.34	0.87	1.25
11JY1668	C6 RIL 352	320	NP	2	46.67	50.00	7.10	2.00	0.61	0.18	1.07	0.79
11JY1669	C6 RIL 354	321	NP	2	19.00	31.00	1.13	0.62	0.13	0.11	1.63	0.24
11JY1670	C6 RIL 355	322	NP	2	37.00	51.33	7.20	2.09	0.66	0.32	1.39	0.98
11JY1671	C6 RIL 356	323	NP	2	52.67	39.67	12.50	5.30	0.97	0.34	0.75	1.31
11JY1672	C6 RIL 358	324	NP	2	51.67	53.67	10.66	3.82	0.87	0.28	1.04	1.15
11JY1673	C6 RIL 361	325	NP	2	41.50	45.00	5.97	2.31	0.53	0.18	1.08	0.71
11JY1674	C6 RIL 362	326	NP	2	55.67	45.67	13.96	4.39	0.83	0.24	0.82	1.07
11JY1675	C6 RIL 364	327	NP	2	68.33	67.67	22.97	8.53	1.44	0.51	0.99	1.95
11JY1676	C6 RIL 366	328	NP	2	51.95	38.45	4.97	2.34	0.46	0.16	0.74	0.62
11JY1677	C6 RIL 368	329	NP	2	52.67	41.67	6.49	1.88	0.48	0.16	0.79	0.64
11JY1678	C6 RIL 372	330	NP	2	64.33	58.33	24.70	6.63	2.05	0.53	0.91	2.58
11JY1680	C6 RIL 379	331	NP	2	37.00	30.00	5.81	1.01	0.22	0.09	0.81	0.31
11JY1682	C6 RIL 382	332	NP	2	55.50	38.00	13.56	4.24	1.25	0.43	0.68	1.68
11JY1683	C6 RIL 388	333	NP	2	63.17	42.00	10.34	5.74	1.20	0.49	0.66	1.69
11JY1684	C6 RIL 391	334	NP	2	64.67	40.33	17.00	7.25	1.27	0.49	0.62	1.76
11JY1686	C6 RIL 394	335	NP	2	66.67	51.67	17.41	5.76	1.23	0.46	0.78	1.69
11JY1687	C6 RIL 395	336	NP	2	65.67	50.33	19.50	6.22	1.22	0.36	0.77	1.58
11JY1688	C6 RIL 398	337	NP	2	71.00	43.00	16.84	7.37	1.22	0.44	0.61	1.66
11JY1689	C6 RIL 400	338	NP	2	72.00	42.67	14.01	6.33	1.16	0.45	0.59	1.61
11JY2045	(CUBA/GUAD C1 F27-4-3-3-B-1-Bx[KILIMA ST94A]-30/MSV-03-2-10-B-2-B-B)-160-1-B-3-B	339	NP	2	61.46	52.15	11.33	2.60	0.67	0.20	0.85	0.87
11JY2047	[[MSRXPOOL9]C1F2-176-4-7-X-1-B/CML206]-5-2-3-1-BBBBB-B-B-B	340	NP	2	65.96	49.48	18.42	4.45	1.17	0.33	0.75	1.50

Seed ID	Acc	Gen	trt.	rep	SL	RL	SFW	RFW	SDW	RDW	RSR	TDM
11JY2051	[CML199/[EV7992#/EV8449-SR]C1F2-334-1(OSU8i)-6-3-Sn]-B-23-2-2-B*4-B-B-B	341	NP	2	47.93	56.13	10.17	3.86	0.80	0.15	1.17	0.95
11JY2052	[CML312/[TUXPSEQ]C1F2/P49-SR]F2-45-3-2-1-BB//INTA-F2-192-2-1-1-1-BBBB]-1-5-1-1-1-BBB-B-B-B	342	NP	2	60.96	37.15	15.69	4.23	0.97	0.29	0.61	1.26
11JY2053	[CML312/CML445//[TUXPSEQ]C1F2/P49-SR]F2-45-3-2-1-BBB]-1-2-1-1-2-BBB-B-B-B	343	NP	2	47.79	36.30	7.94	2.10	0.46	0.09	0.76	0.54
11JY2054	[CML312/MAS[MSR/312]-109-3]-B-71-3-BBB-B-B-B	344	NP	2	60.63	43.82	14.23	3.43	0.92	0.26	0.72	1.18
11JY2055	[CML389/CML176]-B-29-2-2-B*5	345	NP	2	58.63	57.15	14.96	4.09	0.89	0.27	0.97	1.16
11JY2056	[CML395/CML440//[LPSC3H144-1-2-2-2-4-#-BB/SC/ZM605#b-19-2-X]-1-2-X-1-1-BB]-1-2-1-1-B]-3-2-1-1-BBB-B-B-B	346	NP	2	41.46	42.30	4.80	2.04	0.37	0.18	1.02	0.54
11JY2060	[CML444/ZSR923S4BULK-2-2-X-X-X-X-1-BB]-1-1-1-2/CML441]-1-1-1-2-BBB-B-B-B	347	NP	2	38.13	41.97	2.44	0.99	0.18	0.03	1.10	0.20
11JY2061	[DRB-F2-180-2/DRB-3-4-1]-X-6-1-3-BB-2-BBBBBB-B-B-B	348	NP	2	42.79	36.30	6.43	1.87	0.57	0.15	0.85	0.72
11JY2063	[DTPWC8F31-4-2-1-6-B2/CML395//[CML445/ZM621B]-2-1-2-3-1-BB]-3-2-1-1-1-2-B-B-B	349	NP	2	51.46	49.65	11.58	2.54	0.65	0.18	0.96	0.83
11JY2064	[Ent320:92SEW2-77/[DMRESR-W]EarlySel-#I-2-4-B/CML386]-B-11-3-B-2-#-B*4	350	NP	2	35.79	37.30	3.14	1.49	0.35	0.08	1.04	0.43
11JY2065	[LZ956441/LZ966205]-B-3-4-4-B-5-BBBBB-B-B-B	351	NP	2	49.46	53.30	7.79	3.01	0.74	0.21	1.08	0.94
11JY2066	[MSRXPOOL9]C1F2-205-1(OSU23i)-5-3-X-X-1-B//EV7992/EV8449...-3-2-2-1-BBBBB-B-B-B	352	NP	2	48.13	61.97	7.20	2.48	0.75	0.18	1.29	0.93
11JY2067	[SYN-USAB2/SYN-ELIB2]-12-1-1-1-B*4-B-B-B	353	NP	2	53.79	68.63	11.89	5.18	0.97	0.26	1.28	1.22
11JY2070	02SADVE2B-#-42-1-1-1-1-B-B-B	354	NP	2	43.46	50.63	5.55	1.81	0.41	0.09	1.17	0.50
11JY2071	02SADVL2B-#-16-2-1-B-B-B	355	NP	2	44.79	47.63	4.40	1.92	0.30	0.06	1.06	0.36
11JY2075	20V-18	356	NP	2	52.79	38.63	10.59	2.72	0.80	0.11	0.73	0.91
11JY2076	622016-ZCN-2	357	NP	2	56.79	38.97	9.04	2.43	0.54	0.08	0.69	0.62
11JY2078	761BB2 BCox751B-B-1-1-B-B-B-B-B-B	358	NP	2	52.46	45.30	9.57	4.45	0.90	0.27	0.86	1.18
11JY2079	BRAZ 2309	359	NP	2	47.13	45.63	2.47	1.01	0.20	0.05	0.97	0.25
11JY2081	CL-04934 (P49C2H12-5-4xP23C2-11-1)-2-2-2-B*10	360	NP	2	56.96	43.65	13.36	4.21	0.90	0.25	0.77	1.15
11JY2083	CML103	361	NP	2	62.79	48.30	10.14	3.30	0.81	0.19	0.77	1.01
11JY2085	CML114	362	NP	2	43.13	34.97	4.44	1.71	0.32	0.08	0.81	0.41
11JY2086	CML115	363	NP	2	50.79	38.97	6.22	2.37	0.42	0.13	0.77	0.56

Seed ID	Acc	Gen	trt.	rep	SL	RL	SFW	RFW	SDW	RDW	RSR	TDM
11JY2087	CML116	364	NP	2	46.13	40.97	4.59	1.45	0.29	0.05	0.89	0.34
11JY2088	CML118	365	NP	2	44.13	53.30	4.49	2.39	0.36	0.07	1.21	0.43
11JY2090	CML127	366	NP	2	48.13	54.97	6.01	2.68	0.52	0.10	1.14	0.63
11JY2091	CML130	367	NP	2	56.13	42.30	8.38	3.02	0.45	0.31	0.75	0.76
11JY2092	CML133	368	NP	2	47.13	46.30	7.34	3.47	0.52	0.19	0.98	0.71
11JY2093	CML134	369	NP	2	46.79	36.30	4.59	0.93	0.40	0.06	0.78	0.46
11JY2094	CML135	370	NP	2	56.13	40.97	12.73	4.54	0.90	0.28	0.73	1.18
11JY2102	CML169	371	NP	2	54.33	31.80	7.27	2.57	0.53	0.19	0.59	0.72
11JY2103	CML170	372	NP	2	42.83	30.80	6.28	2.90	0.50	0.21	0.72	0.71
11JY2107	CML192	373	NP	2	59.16	38.47	8.23	1.85	0.55	0.17	0.65	0.72
11JY2108	CML20	374	NP	2	49.49	33.13	6.32	2.29	0.51	0.17	0.67	0.68
11JY2109	CML202	375	NP	2	51.49	30.80	7.97	2.63	0.57	0.17	0.60	0.74
11JY2110	CML206	376	NP	2	55.16	45.80	10.44	3.34	0.73	0.22	0.83	0.95
11JY2112	CML226	377	NP	2	47.83	30.13	4.81	1.37	0.34	0.14	0.63	0.47
11JY2114	CML229	378	NP	2	56.49	30.47	11.87	4.94	0.87	0.31	0.54	1.18
11JY2117	CML283	379	NP	2	47.83	31.80	7.74	2.67	0.60	0.20	0.66	0.80
11JY2122	CML290	380	NP	2	53.16	41.13	7.96	3.52	0.57	0.24	0.77	0.81
11JY2125	CML304	381	NP	2	59.49	38.13	13.28	4.73	1.01	0.34	0.64	1.35
11JY2126	CML31	382	NP	2	47.16	34.80	5.85	1.81	0.41	0.15	0.74	0.56
11JY2127	CML311/MBR C3 BC F23-1-2-1-B-B-B	383	NP	2	51.16	38.80	7.48	2.68	0.48	0.17	0.76	0.65
11JY2128	CML311/MBR C3 BC F3-1-1-1-B-B-B	384	NP	2	60.49	40.13	10.57	3.66	0.82	0.30	0.66	1.12
11JY2129	CML311/MBR C3 BC F3-1-1-2-B-B	385	NP	2	53.33	43.80	6.61	2.33	0.49	0.15	0.82	0.64
11JY2132	CML311/MBR C3 BC F43-2-1-1-B-B-B	386	NP	2	62.49	41.47	13.51	4.69	0.98	0.33	0.66	1.31
11JY2133	CML311/MBR C3 BC F65-1-2-2-B-B-B	387	NP	2	50.16	38.80	6.55	2.10	0.47	0.17	0.77	0.64
11JY2134	CML311/MBR C3 BC F95-2-2-1-B-B-B	388	NP	2	54.16	40.80	9.64	3.25	0.75	0.25	0.75	1.00
11JY2136	CML312SR	389	NP	2	41.49	31.47	4.71	1.58	0.37	0.14	0.76	0.52
11JY2137	CML312SRQ=[[(CLQ-RCWQ83xCML312SR)xCML312SR]xCML312SR)]-15-1-BBB	390	NP	2	53.16	29.47	7.53	2.77	0.66	0.25	0.55	0.91
11JY2140	CML322	391	NP	2	52.33	35.30	9.09	4.00	0.70	0.29	0.67	0.99
11JY2141	CML323	392	NP	2	54.16	41.13	8.51	3.02	0.75	0.23	0.76	0.99
11JY2142	CML325	393	NP	2	57.83	47.80	8.69	3.59	0.71	0.26	0.83	0.97
11JY2144	CML328	394	NP	2	45.16	33.13	7.76	2.46	0.61	0.21	0.73	0.81
11JY2146	CML338	395	NP	2	55.16	44.30	9.34	2.10	0.75	0.13	0.80	0.88
11JY2147	CML360	396	NP	2	46.99	30.97	7.75	2.35	0.66	0.13	0.66	0.79
11JY2148	CML361	397	NP	2	56.83	43.30	11.90	3.81	0.86	0.22	0.76	1.08

Seed ID	Acc	Gen	trt.	rep	SL	RL	SFW	RFW	SDW	RDW	RSR	TDM
11JY2149	CML364	398	NP	2	46.08	34.65	6.64	2.12	0.37	0.16	0.75	0.53
11JY2150	CML380xMBR/MDR C3 BC F21-1-1-2-B-B-B-B-3-1-B-B-B	399	NP	2	35.74	44.32	3.39	1.64	0.24	0.11	1.24	0.34
11JY2151	CML384xMBR/MDR C3 BC F58-2-1-3-B-B-B-B-3-1-B-B-B	400	NP	2	52.74	27.65	7.92	2.07	0.61	0.16	0.52	0.77
11JY2152	CML389	401	NP	2	59.08	34.32	11.60	3.64	0.93	0.29	0.58	1.22
11JY2153	CML389/CML144//CML159//POOL15QPMSR-B-6-B-B	402	NP	2	51.74	37.98	9.65	1.93	0.71	0.28	0.73	0.99
11JY2154	CML40	403	NP	2	49.08	40.32	7.91	2.63	0.63	0.27	0.82	0.89
11JY2155	CML402	404	NP	2	47.74	23.65	4.43	1.45	0.34	0.14	0.50	0.48
11JY2157	CML411	405	NP	2	56.74	42.98	8.01	3.09	0.57	0.19	0.76	0.75
11JY2160	CML423	406	NP	2	58.41	45.98	14.19	4.09	0.77	0.26	0.79	1.03
11JY2162	CML428	407	NP	2	41.08	30.32	4.49	1.45	0.41	0.12	0.74	0.53
11JY2163	CML430	408	NP	2	45.74	34.98	3.76	1.69	0.39	0.13	0.76	0.51
11JY2164	CML431	409	NP	2	52.24	29.48	5.44	1.70	0.35	0.12	0.56	0.47
11JY2165	CML432	410	NP	2	54.08	23.65	7.15	1.31	0.47	0.10	0.44	0.57
11JY2166	CML433	411	NP	2	33.08	22.32	2.39	0.83	0.22	0.13	0.67	0.35
11JY2168	CML445/CML144//CML159//POOL15QPMSR-B-55-B-B	412	NP	2	43.74	29.32	9.12	2.19	0.74	0.20	0.67	0.93
11JY2170	CML454	413	NP	2	49.74	40.98	7.21	2.87	0.75	0.30	0.82	1.04
11JY2172	CML468	414	NP	2	67.74	33.98	10.21	2.95	0.90	0.21	0.50	1.11
11JY2173	CML470	415	NP	2	63.24	35.48	13.98	5.51	0.93	0.32	0.56	1.25
11JY2176	CML479	416	NP	2	42.74	29.98	5.62	1.51	0.58	0.16	0.70	0.75
11JY2177	CML480	417	NP	2	51.74	37.48	8.17	3.43	0.62	0.30	0.72	0.92
11JY2180	CML496	418	NP	2	51.41	57.98	3.93	1.50	0.33	0.11	1.13	0.44
11JY2189	CML80	419	NP	2	56.08	46.98	9.39	2.67	0.78	0.20	0.84	0.97
11JY2192	CML94	420	NP	2	60.74	49.98	8.43	3.09	0.60	0.20	0.82	0.80
11JY2193	CML96	421	NP	2	38.74	33.65	2.46	0.26	0.23	0.06	0.87	0.30
11JY2194	CML99	422	NP	2	40.74	25.98	4.81	1.75	0.38	0.15	0.64	0.53
11JY2196	Cuba/GuadC3F125-2-2-1-B-B-B	423	NP	2	52.91	47.22	11.84	2.59	0.63	0.23	0.89	0.86
11JY2197	CY9169	424	NP	2	55.22	27.06	10.20	2.33	0.69	0.16	0.49	0.85
11JY2198	DTPW C9	425	NP	2	52.55	34.06	9.21	2.75	0.72	0.19	0.65	0.91
11JY2199	DTPWC9-F104-5-4-1-1-B-B-B	426	NP	2	66.05	52.56	13.98	3.50	1.08	0.24	0.80	1.32
11JY2200	DTPY C9	427	NP	2	42.55	36.56	8.63	2.70	0.67	0.22	0.86	0.88
11JY2201	DTPYC9-F46-1-2-1-2-B-B	428	NP	2	59.22	41.73	9.79	2.66	0.68	0.19	0.70	0.88
11JY2202	DTPYC9-F46-3-9-1-1-B-BTL-07B 6614-42	429	NP	2	48.05	35.06	6.90	2.10	0.44	0.11	0.73	0.55

Seed ID	Acc	Gen	trt.	rep	SL	RL	SFW	RFW	SDW	RDW	RSR	TDM
11JY2204	Guad 6	430	NP	2	56.88	43.73	11.55	3.68	0.76	0.22	0.77	0.98
11JY2205	H-16	431	NP	2	57.55	55.73	9.96	4.62	0.91	0.30	0.97	1.21
11JY2207	La Posta Seq C7-F125-2-1-1-2-B-B-B	432	NP	2	48.55	43.73	10.34	3.51	0.72	0.22	0.90	0.94
11JY2211	La Posta Seq C7-F64-2-6-1-2-B-B-B	433	NP	2	58.05	47.56	12.12	4.48	0.84	0.29	0.82	1.13
11JY2216	La Posta Seq C7-F96-1-2-1-2-B-B	434	NP	2	47.22	53.39	9.50	3.63	0.62	0.22	1.13	0.85
11JY2218	LPSC7	435	NP	2	58.55	58.73	11.20	3.16	0.72	0.20	1.00	0.92
11JY2220	MAS[206/312]-23-2-1-1-B*6-B-B-B	436	NP	2	56.55	35.73	11.54	2.77	0.78	0.22	0.63	1.00
11JY2223	MBR C6 BC F234-1-B-#-1-1-B-B-B-B-B	437	NP	2	51.88	44.06	10.24	2.60	0.73	0.18	0.85	0.91
11JY2225	P402c2F2-695-2-BB-2-B*4-1-B	438	NP	2	48.05	48.56	10.54	2.59	0.66	0.21	1.01	0.87
11JY2228	P591c4 F55-2-2-2-B-B-B	439	NP	2	71.05	39.56	9.00	5.21	0.76	0.17	0.56	0.93
11JY2229	P591c41y2GENF3-1-1-2-B-B-B	440	NP	2	45.55	40.06	7.43	2.14	0.44	0.15	0.88	0.59
11JY2230	PAZM 6053	441	NP	2	42.05	38.06	7.86	2.08	0.54	0.15	0.91	0.69
11JY2231	Pool 21 x Pool 22	442	NP	2	47.22	30.73	7.35	2.29	0.48	0.14	0.65	0.62
11JY2233	R15	443	NP	2	55.55	51.06	10.26	2.68	0.69	0.23	0.92	0.92
11JY2234	RDOM 330	444	NP	2	45.05	30.06	8.00	2.30	0.54	0.13	0.67	0.67
11JY2238	VL0512452	445	NP	2	52.88	35.06	10.30	2.86	0.73	0.18	0.66	0.91
11JY2239	VL0512464	446	NP	2	58.22	41.39	11.09	2.29	0.81	0.17	0.71	0.98
11JY2240	VL05128	447	NP	2	57.54	45.85	11.04	2.53	0.56	0.16	0.80	0.71
11JY2241	VL052	448	NP	2	68.54	37.85	14.72	2.79	0.89	0.19	0.55	1.08
11JY2243	VL05353	449	NP	2	71.71	48.35	18.58	4.31	1.13	0.26	0.67	1.40
11JY2244	VL054178	450	NP	2	49.54	39.18	10.49	2.48	0.61	0.14	0.79	0.76
11JY2246	VL054881	451	NP	2	61.88	49.85	11.96	3.15	0.71	0.19	0.81	0.90
11JY2248	VL0556	452	NP	2	49.21	52.52	9.99	2.60	0.55	0.17	1.07	0.72
11JY2249	VL05561	453	NP	2	56.54	49.18	11.60	3.52	0.66	0.22	0.87	0.88
11JY2250	VL0558	454	NP	2	45.88	44.52	11.18	2.74	0.61	0.20	0.97	0.81
11JY2252	VL05616	455	NP	2	51.83	42.80	10.41	2.47	0.57	0.18	0.83	0.75
11JY2255	VL056942	456	NP	2	60.83	58.30	13.85	3.58	0.86	0.28	0.96	1.14
11JY2259	VL062784	457	NP	2	54.33	38.80	9.94	2.19	0.53	0.14	0.71	0.67
11JY2260	VL062785	458	NP	2	66.33	39.80	14.47	3.24	0.89	0.24	0.60	1.13
11JY2262	VL06384	459	NP	2	47.99	31.97	10.65	2.40	0.65	0.10	0.67	0.75
11JY2263	ZM521B-66-4-1-1-BB-B-B-B	460	NP	2	59.74	46.88	12.46	3.11	0.65	0.24	0.78	0.89
11JY2264	川29♀	461	NP	2	60.24	45.55	12.79	2.70	0.68	0.21	0.76	0.89
11JY2265	慈溪白糯	462	NP	2	48.74	44.88	10.50	2.59	0.51	0.21	0.92	0.72
11JY2266	独紫	463	NP	2	69.24	55.88	13.57	3.38	0.71	0.26	0.81	0.97
11JY2268	交51	464	NP	2	56.91	43.22	12.45	2.84	0.72	0.24	0.76	0.95
11JY2269	双M9	465	NP	2	47.91	40.55	10.91	2.48	0.55	0.22	0.85	0.77

Seed ID	Acc	Gen	trt.	rep	SL	RL	SFW	RFW	SDW	RDW	RSR	TDM
11JY2270	四川地方种质	466	NP	2	69.24	50.88	15.98	3.74	0.97	0.35	0.73	1.32
11JY2271	豫综BC15-2	467	NP	2	55.24	39.55	11.27	2.70	0.59	0.21	0.72	0.79
11JY2279	407	468	NP	2	50.58	36.88	11.00	2.35	0.53	0.21	0.73	0.74
11JY2280	412	469	NP	2	36.99	36.97	5.72	1.54	0.35	0.09	1.00	0.44
11JY2288	485	470	NP	2	50.49	44.97	12.83	3.66	0.79	0.19	0.89	0.98
11JY2290	495	471	NP	2	51.99	39.47	9.49	3.10	0.62	0.20	0.76	0.82
11JY2311	8001	472	NP	2	57.83	39.80	11.86	2.66	0.66	0.18	0.69	0.84
11JY2317	[(CML395/CML444)-B-4-1-3-1-B/CML395//DTPWC8F31-1-1-2-2]-5-1-2-2-BB-B-B-B	473	NP	2	41.99	62.47	6.06	2.02	0.46	0.08	1.49	0.54
11JY2327	4F1	474	NP	2	47.49	40.63	8.86	3.04	0.61	0.17	0.86	0.77
11JY2356	B73	475	NP	2	54.99	34.63	11.89	2.29	0.88	0.21	0.63	1.09
11JY2391	E28	476	NP	2	50.13	35.63	10.15	2.40	0.80	0.17	0.71	0.97
11JY2394	ES40	477	NP	2	42.49	31.47	8.61	1.99	0.59	0.09	0.74	0.68
11JY2396	F42	478	NP	2	61.79	46.97	20.02	8.32	1.22	0.38	0.76	1.60
11JY2398	FR19	479	NP	2	44.79	55.97	6.38	2.70	0.45	0.10	1.25	0.55
11JY2426	MBNA	480	NP	2	60.21	48.18	13.13	3.58	0.76	0.23	0.80	0.99
11JY2434	NS701	481	NP	2	56.21	43.85	11.04	2.67	0.58	0.16	0.78	0.74
11JY2445	PHG83	482	NP	2	57.21	46.18	12.13	3.10	0.65	0.22	0.81	0.87
11JY2446	PHN47	483	NP	2	54.21	42.35	13.69	3.76	0.79	0.20	0.78	1.00
11JY2453	R09	484	NP	2	54.49	26.97	11.69	3.27	0.98	0.23	0.49	1.21
11JY2467	Va35	485	NP	2	52.83	42.13	11.70	2.78	0.57	0.18	0.80	0.75
11JY2470	W8304	486	NP	2	62.16	41.80	13.36	2.82	0.81	0.23	0.67	1.04
11JY2474	XZY364-1	487	NP	2	43.16	47.97	2.15	1.81	0.35	0.09	1.11	0.45
11JY2491	长3154	488	NP	2	55.83	51.80	12.82	3.30	0.74	0.29	0.93	1.03
11JY2498	丹3130	489	NP	2	52.33	47.30	10.23	3.19	0.52	0.24	0.90	0.76
11JY2500	丹340	490	NP	2	45.66	34.80	5.68	2.35	0.40	0.14	0.76	0.54
11JY2504	丹360	491	NP	2	56.83	44.13	11.19	2.54	0.62	0.17	0.78	0.79
11JY2522	辐746	492	NP	2	63.83	43.47	13.00	2.91	0.78	0.22	0.68	1.00
11JY2526	旱21	493	NP	2	58.83	44.80	10.88	2.71	0.57	0.23	0.76	0.80
11JY2547	吉419	494	NP	2	75.83	67.47	17.94	4.58	1.11	0.32	0.89	1.43
11JY2557	吉846	495	NP	2	68.88	46.52	13.90	3.17	0.84	0.21	0.68	1.05
11JY2563	冀研01-3-2-2-1-5-1	496	NP	2	53.88	47.85	11.84	2.88	0.59	0.14	0.89	0.73
11JY2570	金黄96C	497	NP	2	62.71	46.35	13.35	3.50	0.67	0.18	0.74	0.86
11JY2574	辽138	498	NP	2	55.54	48.18	13.52	3.27	0.73	0.19	0.87	0.92

Seed ID	Acc	Gen	trt.	rep	SL	RL	SFW	RFW	SDW	RDW	RSR	TDM
11JY2587	辽孤001	499	NP	2	56.58	45.55	13.73	2.72	0.64	0.23	0.81	0.86
11JY2603	齐205	500	NP	2	57.58	41.88	12.34	2.71	0.72	0.23	0.73	0.96
11JY2606	齐310	501	NP	2	49.58	39.22	11.06	2.46	0.50	0.20	0.79	0.70
11JY2621	双105	502	NP	2	58.24	43.22	12.04	2.60	0.64	0.18	0.74	0.81
11JY2623	双741	503	NP	2	55.91	45.55	11.88	2.55	0.62	0.21	0.81	0.83
11JY2667	郑22	504	NP	2	68.88	51.52	20.51	4.74	1.15	0.27	0.75	1.42
11JY2672	郑29	505	NP	2	69.71	57.85	20.07	3.70	1.15	0.26	0.83	1.41
11JY2675	郑30	506	NP	2	61.54	52.85	14.24	3.61	0.78	0.15	0.86	0.94
11JY2677	郑35	507	NP	2	59.54	56.52	15.35	3.14	0.86	0.20	0.95	1.06
11JY2697	综31	508	NP	2	64.21	36.85	14.74	3.10	0.83	0.20	0.57	1.03
12JY0001	C5 RIL P2	509	NP	2	53.83	43.30	10.64	4.15	0.71	0.25	0.80	0.96
12JY0002	C5 RIL P1	510	NP	2	52.33	44.13	8.47	3.11	0.53	0.14	0.84	0.68
12JY0015	[(SML*SMQPM)*(MTL*SMQPM)]F1S6-1-25-BB-1-B	511	NP	2	68.49	32.97	12.65	3.64	0.58	0.21	0.48	0.79
12JY0017	[CML159/[CML159/[MSRXPOOL9]C1F2-205-1(OSU23i)-5-3-X-X-1-BB]F2-3sx]-8-1-1-BB-1-B	512	NP	2	53.16	40.30	12.14	3.46	0.63	0.22	0.76	0.85
12JY0018	[CML198/LPSC3H144-1-2-2-2-2-#-BB]-1-4-1-1-4-B*4-B-B-B	513	NP	2	51.83	50.97	10.22	3.62	0.68	0.28	0.98	0.95
12JY0029	[DTPWC8F31-4-2-1-6/CML444//ZM521B-66-4-1-1-1-BB]-3-2-1-B-B-B	514	NP	2	40.33	42.13	4.90	2.14	0.42	0.13	1.04	0.56
12JY0040	18-599	515	NP	2	60.16	25.97	6.19	1.21	0.55	0.13	0.43	0.68
12JY0041	18-599(RED)	516	NP	2	46.64	37.55	6.27	1.07	0.48	0.07	0.81	0.55
12JY0108	CML330	517	NP	2	45.49	65.97	7.59	2.24	0.69	0.19	1.45	0.88
12JY0122	CML418	518	NP	2	46.83	43.30	6.89	1.47	0.63	0.12	0.92	0.75
12JY0146	CML504	519	NP	2	45.16	45.97	5.01	1.11	0.46	0.11	1.02	0.57
12JY0156	Cuba/Guad C3 F53-3-1-1-B-B-B	520	NP	2	55.16	37.30	7.86	2.44	0.81	0.13	0.68	0.94
12JY0164	ECA-MOROSR(BC1)F2-7-ECAVEE7/PL15QPMC7SRC1F2//POOL15QPMSR-B-4-B-B	521	NP	2	46.16	31.97	8.21	2.86	0.49	0.14	0.69	0.63
12JY0167	INTA-191-2-1-2-B*8-B-B-B	522	NP	2	47.49	42.97	6.43	1.88	0.47	0.08	0.90	0.55
12JY0173	La Posta Seq C7-F86-1-1-1-1-B-B-B	523	NP	2	36.16	34.63	2.81	1.17	0.25	0.08	0.96	0.33
12JY0185	P501SRc0-F2-47-3-1-1-B-B-B-B	524	NP	2	37.16	45.30	2.05	1.00	0.31	0.06	1.22	0.37
12JY0208	VL05610	525	NP	2	55.83	29.13	4.40	1.75	0.55	0.13	0.52	0.68
12JY0226	178	526	NP	2	34.83	19.97	2.65	1.42	0.36	0.09	0.57	0.45
12JY0228	273	527	NP	2	58.12	45.84	10.97	2.64	0.62	0.18	0.79	0.79

Seed ID	Acc	Gen	trt.	rep	SL	RL	SFW	RFW	SDW	RDW	RSR	TDM
12JY0229	288	528	NP	2	50.83	31.63	7.16	2.23	0.58	0.13	0.62	0.71
12JY0240	764	529	NP	2	49.33	44.30	11.30	2.89	0.69	0.16	0.90	0.85
12JY0246	6103	530	NP	2	55.49	50.97	10.47	3.38	0.54	0.16	0.92	0.70
12JY0252	81565	531	NP	2	57.33	39.13	9.00	2.85	0.64	0.14	0.68	0.78
12JY0264	634-11511	532	NP	2	53.49	43.63	9.30	1.92	0.78	0.14	0.82	0.92
12JY0265	698-1	533	NP	2	45.49	42.63	7.48	1.68	0.53	0.09	0.94	0.62
12JY0308	FAPW	534	NP	2	66.49	42.80	16.98	3.66	1.14	0.30	0.64	1.44
12JY0322	LH132	535	NP	2	57.16	39.80	12.49	2.64	0.71	0.24	0.70	0.95
12JY0323	LH51	536	NP	2	48.66	30.63	9.92	2.60	0.48	0.17	0.63	0.65
12JY0325	LX9801	537	NP	2	54.66	35.13	7.65	1.94	0.52	0.15	0.64	0.66
12JY0329	Mo17	538	NP	2	54.16	27.47	14.19	2.63	0.54	0.19	0.51	0.72
12JY0347	R08	539	NP	2	59.16	33.13	8.15	2.26	0.51	0.16	0.56	0.67
12JY0349	RP125	540	NP	2	52.16	49.55	14.39	3.31	1.00	0.21	0.95	1.22
12JY0365	Zhao835	541	NP	2	51.49	31.47	15.36	4.21	0.72	0.32	0.61	1.03
12JY0370	昌7-2	542	NP	2	55.91	50.97	13.72	3.26	0.86	0.22	0.91	1.07
12JY0383	丹598	543	NP	2	54.99	29.72	10.77	2.83	0.84	0.24	0.54	1.08
12JY0401	黄早四	544	NP	2	46.41	43.97	9.81	2.49	0.64	0.17	0.95	0.81
12JY0416	吉853	545	NP	2	57.83	47.47	13.15	3.34	0.60	0.22	0.82	0.82
12JY0443	南21-3	546	NP	2	64.91	49.30	15.54	4.08	1.06	0.32	0.76	1.38
12JY0450	齐319	547	NP	2	54.20	41.51	9.74	2.55	0.54	0.19	0.77	0.73
12JY0457	沈5003	548	NP	2	46.16	49.13	6.82	2.10	0.39	0.16	1.06	0.55
12JY0462	四287	549	NP	2	54.91	43.55	12.13	3.21	0.74	0.18	0.79	0.92
12JY0473	铁7922	550	NP	2	54.49	32.47	12.58	2.61	0.67	0.19	0.60	0.85
